# C(sp^3^)−C(sp^3^) bond formation via nickel-catalyzed deoxygenative homo-coupling of aldehydes/ketones mediated by hydrazine

**DOI:** 10.1038/s41467-021-23971-7

**Published:** 2021-06-17

**Authors:** Dawei Cao, Chen-Chen Li, Huiying Zeng, Yong Peng, Chao-Jun Li

**Affiliations:** 1 Department of Chemistry and FRQNT Centre for Green Chemistry and Catalysis, McGill University, Montreal, QC Canada; 2 Key Laboratory of Magnetism and Magnetic Materials of the Ministry of Education, School of Physical Science and Technology and Electron Microscopy Centre, Lanzhou University, Lanzhou, PR China; 3 The State Key Laboratory of Applied Organic Chemistry, Lanzhou University, Lanzhou, PR China

**Keywords:** Homogeneous catalysis, Organic chemistry

## Abstract

Aldehydes and ketones are widely found in biomass resources and play important roles in organic synthesis. However, the direct deoxygenative coupling of aldehydes or ketones to construct C(sp^3^)−C(sp^3^) bond remains a scientific challenge. Here we report a nickel−catalyzed reductive homo-coupling of moisture- and air-stable hydrazones generated in-situ from naturally abundant aldehydes and ketones to construct challenging C(sp^3^)−C(sp^3^) bond. This transformation has great functional group compatibility and can suit a broad substrate scope with innocuous H_2_O, N_2_ and H_2_ as the by-products. Furthermore, the application in several biological molecules and the transformation of PEEK model demonstrate the generality, practicability, and applicability of this novel methodology.

## Abstract (web summary)

The direct deoxygenative coupling of aldehydes or ketones to construct C(sp^3^)−C(sp^3^) bond remains a scientific challenge. Here the authors use a nickel−catalyzed reductive homo-coupling of moisture- and air-stable hydrazones generated in-situ from naturally abundant aldehydes and ketones to construct challenging C(sp^3^)−C(sp^3^) bonds.

## Introduction

The prospect of diminishing fossil hydrocarbon resources and the effects of global climate change call for the development of sustainable supply chains for the chemical industry that rely on renewable feedstocks^1,2^. Biomass, as a renewable resource, has the potential to provide earth rich and readily available carbon-based raw materials for chemical industry^3^. Although both biomass and fossil oil are carbon-based natural resources, their compositions are very different: while fossil feedstocks consist primarily of carbon and hydrogen, the oxygen content in biomass resources is very high mostly in the form of carbonyl or hydroxyl groups, which lead to complications and great challenges in the direct substitution of fossil resources with bio-based ones^4–6^. Therefore, the efficient transformation of these oxygen-containing compounds, especially the direct deoxygenation of aldehydes or ketones, could potentially provide an enabling tool for future biorefinery concepts.

The formation of C(sp^3^)−C(sp^3^) bond is one of the most fundamental strategies in organic transformations^7–9^. Especially, dibenzyl derivatives containing C(sp^3^)−C(sp^3^) bond are essential structural motifs in natural products, pharmaceuticals and agrochemicals, as well as dyes and polymers^10–12^. Although the reductions of diarylalkenes and diarylalkynes provide means for the preparation of bibenzyl compounds^13,14^, homo-coupling strategies show superiority to access these compounds. For example, the homo-coupling of benzyl halides^15^, benzylmagnesium halides^16^, phenylacetic acids^17^, benzyl boronic acids^18^ and methylbenzene derivatives^19^ have been disclosed in recent years. However, these methods suffered from several drawbacks such as the tedious prefunctionalization steps, the use of moisture-sensitive organometallic reagents and the requirement of noble metals, which greatly limit their broad applicability. Therefore, the development of a novel approach for the direct formation of C(sp^3^)−C(sp^3^) bond from naturally abundant aldehydes/ketones would be highly desirable.

**Fig. 1:**
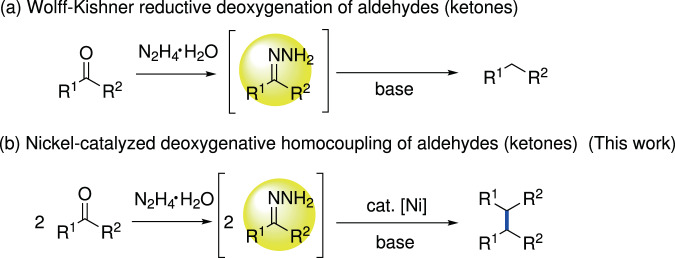
Strategies for deoxygenation of aldehydes and ketones with hydrazine. **a** Wolff–Kishner reductive deoxygenation of aldehydes (ketones). **b** Nickel-catalyzed deoxygenative homocoupling of aldehydes (ketones).

The Wolff–Kishner reduction is a classical direct deoxygenation strategy for converting aldehydes/ketones into alkane derivatives mediated by hydrazine (Fig. 1a)^20^. Inspired by this classical reaction, great progress toward C–C bond construction using simple hydrazones, readily generated in situ from aldehydes/ketones and hydrazine, as nucleophiles through deoxygenative cross-coupling strategies have been made by us^21–27^ and others^28–30^ over the past several years. Moreover, as a strong reductant, hydrazine itself was also successfully applied as a reductant in reductive coupling reactions with aryl halides^31^. Inspired by the transformation of Wolff–Kishner reduction and our previous work^21,31^, we turned to consider whether hydrazine can also serve as a reductant for the reductive coupling of carbonyls to realize C(sp^3^)−C(sp^3^) bond formation.

Here, we show a powerful and reliable strategy in which nickel-catalyzed the direct deoxygenative homo-coupling of aldehydes/ketones mediated by hydrazine to synthesize dibenzyl derivatives (Fig. 1b). Highlighted features of this protocol are: (a) H_2_O, N_2_ and H_2_ as innocuous side products; (b) naturally rich aldehydes/ketones as homo-coupling materials; (c) overcome the competing Wolff–Kishner reaction in the presence of base; (d) inexpensive and earth-abundant nickel as catalyst; (e) broad substrate scope and great functional group tolerance; and (f) synthesis of commercial drug molecule and transformation of PEEK model.

## Results

### Reaction optimization

To initiate the exploration, hydrazone **2a** generated from benzaldehyde (**1a**) was chosen as the model substrate to optimize the reaction conditions (Table 1). The deoxygenative homo-coupling product **3a** was obtained in 30% yield when NiCl_2_ (20 mol%) was used as catalyst in presence of trimethylphosphine (PMe_3_, 40 mol%), using 1,8-diazabicyclo[5.4.0]undec-7-ene (DBU) as base and THF as solvent under an argon atmosphere at 100 °C for 24 h (Table 1, entry 1). Other nickel catalysts, such as NiBr_2_, Ni(acac)_2_∙4H_2_O, Ni(OAc)_2_, NiCl_2_(PPh_3_)_2_ and Ni(cod)_2_, also gave the desired products, but showed relatively lower efficiency (Table 1, entries 2–6). Notably, in absence of any key component, including catalyst, ligand or base, no desired product **3a** was obtained (Table 1, entries 7–9). According to our previous work^26,27^, base plays a key role in the hydrazone conversion process, and the strong base is more favorable than the weak one. Therefore, other bases were examined: organic bases including DABCO and Et_3_N showed lower efficiency than DBU (Table 1, entries 10–11), which is consistent with the previous work; inorganic bases such as KOH and t-BuOK may have poor solubility in the catalytic system, making the reaction yield markedly reduced (Table 1, entries 12–13). In addition to the monodentate phosphine ligand, other phosphorus, nitrogen and carbene ligands were also tested, with N-heterocyclic carbene in situ generated from 1,3-bis(2,4,6-trimethylphenyl)imidazolium chloride (IMes∙HCl) giving the best result (Table 1, entries 14–23). To our satisfaction, the reaction yield reached 65% when using 1,4-dioxane instead of THF as the solvent (Table 1, entry 24). Based on this, when the amount of catalyst and ligand were increased to 30 mol%, 83% yield of **3a** could be obtained (Table 1, entry 25). Finally, the effect of temperature on the reaction was also investigated (Table 1, entries 26–27). When the temperature was decreased to 80 °C, the starting material hydrazone could not be completely converted, indicating that the reaction rate is insufficient at low temperature. Due to the detection of a large amount of Wolff–Kishner reduction by-product at 120 °C, the yield of homo-coupling product decreased significantly.

**Table 1:** Optimization of the reaction conditions^a^.


Entry	Catalyst	Ligand	Base	Solvent	3a yield(%)^b^
1	NiCl_2_	PMe_3_	DBU	THF	25
2	NiBr_2_	PMe_3_	DBU	THF	22
3	Ni(acac)_2_∙4H_2_O	PMe_3_	DBU	THF	7
4	Ni(OAc)_2_	PMe_3_	DBU	THF	18
5	NiCl_2_(PPh_3_)_2_	PMe_3_	DBU	THF	12
6	Ni(cod)_2_	PMe_3_	DBU	THF	20
7	--	PMe_3_	DBU	THF	n.p.
8	NiCl_2_	--	DBU	THF	n.p.
9	NiCl_2_	PMe_3_	--	THF	n.p.
10	NiCl_2_	PMe_3_	DABCO	THF	14
11	NiCl_2_	PMe_3_	Et_3_N	THF	3
12	NiCl_2_	PMe_3_	KOH	THF	n.p.
13	NiCl_2_	PMe_3_	t-BuOK	THF	10
14	NiCl_2_	dppb	DBU	THF	28
15	NiCl_2_	dppe	DBU	THF	32
16	NiCl_2_	dppp	DBU	THF	45
17	NiCl_2_	dppf	DBU	THF	21
18	NiCl_2_	2,2'-bipyridine	DBU	THF	40
19	NiCl_2_	1,10-phenanthroline	DBU	THF	42
20	NiCl_2_	IPr∙HCl	DBU	THF	55
21	NiCl_2_	SIPr∙HCl	DBU	THF	49
22	NiCl_2_	IMes∙HCl	DBU	THF	61
23	NiCl_2_	SIMes∙HCl	DBU	THF	54
24	NiCl_2_	IMes∙HCl	DBU	1,4-dioxane	65
25^c^	NiCl_2_	IMes∙HCl	DBU	1,4-dioxane	86 (83)
26^c, d^	NiCl_2_	IMes∙HCl	DBU	1,4-dioxane	55
27 ^c, e^	NiCl_2_	IMes∙HCl	DBU	1,4-dioxane	71


^a^General conditions: **2a** (2 × 0.1 mmol), catalyst (0.02 mmol, 20 mol%), ligand (monodentate phosphine ligand PMe_3_ 40 mol %, other ligands 20 mol %), base (0.22 mmol, 2.2 equiv.) and solvent (1.0 mL) at 100 °C for 24 h under an argon atmosphere.

^b^Yields were determined by ^1^H NMR with dibromomethane as internal standard; isolated yields in parentheses.

^c^NiCl_2_ (0.03 mmol, 30 mol%), IMes∙HCl (0.03 mmol, 30 mol%).

^d^80 °C.

^e^120 °C.

### Investigation of the substrate scope

**Fig. 2:**
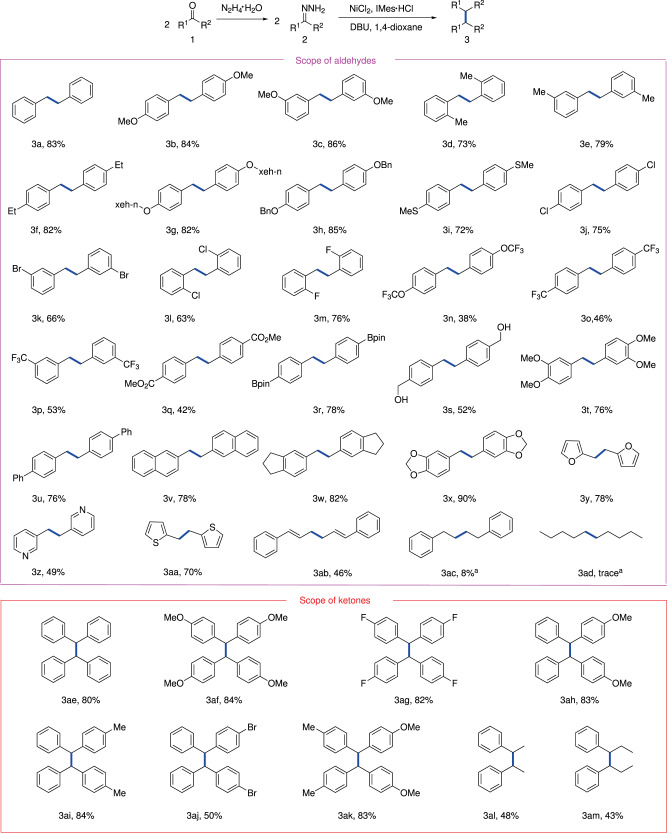
Substrate scope of the homo-coupling reaction. General conditions: **2** (2 × 0.1 mmol), NiCl_2_ (0.03 mmol, 30 mol%), IMes∙HCl (0.03 mmol, 30 mol%), DBU (0.22 mmol, 2.2 equiv) and 1,4-dioxane (1.0 mL) at 100 °C for 24 h under an argon atmosphere, isolated yields. ^**a**^ Detected by GC-MS.

To assess the generality of this method, the scope of the nickel-catalyzed deoxygenative homo-coupling process was examined, as shown in Fig. 2. In general, this reaction demonstrated good functional group tolerance with respect to the substitution patterns and electronic properties of hydrazones generated from aldehydes. A variety of aryl aldehydes bearing electron-donating groups (methoxy-, methyl-, ethyl-, alkoxy- and methylthio-) or electron-withdrawing groups (halogen-, trifluoromethoxy- trifluoromethyl- and ester-) at different positions (ortho-, meta- or para-) of the aryl ring were smoothly converted to the homo-coupling products in moderate to excellent yields (**3b-3q**). It was noteworthy that the hydrazone bearing a borate group was able to deliver the corresponding dibenzyl boronic esters **3r**, which provides a handle for downstream transformations. Furthermore, the substrate bearing an unprotected hydroxymethyl group gave the product **3s**. In addition, the multisubstituted substrate could be tolerated in this catalytic system, affording the desired product **3t**. To our gratification, various polycyclic (hetero-) aromatic structures such as biphenyl, naphthalene, indene, dioxole, furan, pyridine and thiophene on aldehydes could also be tolerated in the transformation with moderate to good yields (**3u**-**3aa**). Because 1,5-dienes are valuable building blocks in organic synthesis and medicinal chemistry^32^, the desired 1,5-diene product **3ab** was successfully synthesized through this nickel-catalyzed system using cinnamaldehyde-derived hydrazone as a substrate. Unfortunately, for valeraldehyde and phenylacetaldehyde substrates, only 8% or trace yield of the desired products (**3ac**-**3ad**) was detected by GC-MS, probably due to the competing azine formation under high temperature.

Subsequently, the scope of hydrazones generated from ketones was investigated (Fig. 2). Symmetric diaryl ketones bearing various substituents were suitable substrates, providing the corresponding products **3ae**-**3ag** in excellent yields. To our delight, the homo-coupling reaction also occurred smoothly with non-symmetrical diaryl ketones as substrates, afforded the dibenzyl products (**3ah**-**3aj**) in 46–84% yields. In particular, the non-symmetrical diphenyl ketone (4-methoxyphenyl)(*p*-tolyl)methanone, which contains different substituents, gave the homo-coupling product **3ak**. Moreover, aryl alkyl ketone, such as acetophenone and propiophenone, were all suitable substrates for this transformation, providing moderate yields of the desired products **3al**-**3am**.

### Applications

**Fig. 3:**
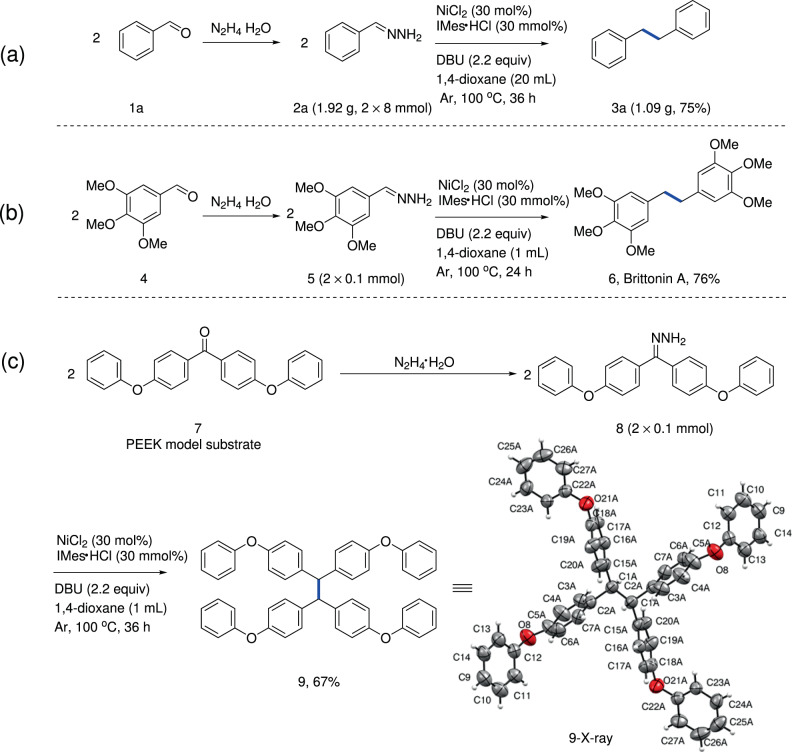
Applications of the deoxygenative aldehyde/ketone homo-coupling. **a** Gram-scale experiment. **b** Synthesis of Britonin A. **c** Conversion of polyetheretherketone (PEEK) model compound.

To further exploit the practicality of current protocol, a gram-scale experiment of **2a** generated from benzaldehyde (**1a**) was conducted, and the product **3a** was obtained in 75% yield (1.09 g) after 36 h (Fig. 3a). In addition, to evaluate its synthetic applications, the drug molecule Britonin A (**6**) was synthesized from naturally abundant **4** under standard reaction conditions (Fig. 3b). Polyetheretherketone (PEEK), being one of the most commonly used engineering plastics in industry, has caused waste accumulation problems due to its stability^33^. Therefore, the conversion of disused PEEK into other useful chemicals is a sustainability challenge. To our delight, when the PEEK model compound **7** (Fig. 3c) was tested with this nickel-catalyzed system, the desired homo-coupling product **9** was obtained in 67% yield, which was confirmed by X-ray analysis (see the Supplementary Information). These results indicated that the utility of our protocol for the late stage synthesis and modification of complex molecules.

### Mechanistic studies

**Fig. 4:**
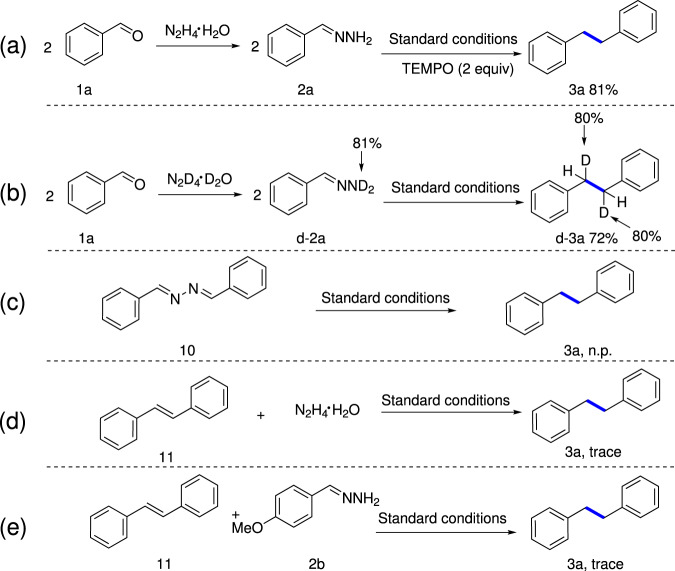
Mechanistic studies. **a** Free radical capture experiment. **b** Deuterium-labeling experiment. **c** Experiment to verify possible azine intermediate. **d** Experiment to verify possible intermediate (*E*)−1,2-diphenylethene reacting with hydrazine. **e** Experiment to verify possible intermediate (*E*)−1,2-diphenylethene reacting with hydrazone **2b**.

In order to gain mechanistic insights, some preliminary experiments were subsequently carried out. Firstly, the efficiency of the model reaction was almost unaffected in the presence of free radical initiator 2,2,6,6-tetramethyl-1-piperidinyloxy (TEMPO), demonstrating an unlikely radical pathway in this transformation (Fig. 4a). Secondly, deuterium-labeling experiment using deuterated hydrazone **d-2a** was performed under standard conditions, and the homo-coupling product **d-3a** was obtained in 72% yield with 80% deuteration at benzylic position, which attributed the origination of hydrogen at the benzylic position to the N–H group in hydrazone (Fig. 4b). Thirdly, dibenzalhydrazine (**10**), which appeared at a trace amount in the reaction, was prepared and examined under the standard conditions, and no desired product **3a** was detected, demonstrating the improbable process involving azine as a reaction intermediate (Fig. 4c). Finally, when (*E*)−1,2-diphenylethene (**11**) was tested, only a small amount of the corresponding product **3a** was obtained in the presence of hydrazine (Fig. 4d) or hydrazone **2b** (Fig. 4e), indicating that the (*E*)−1,2-diphenylethene is unlikely an intermediate in this deoxygenative homo-coupling process either.

**Fig. 5:**
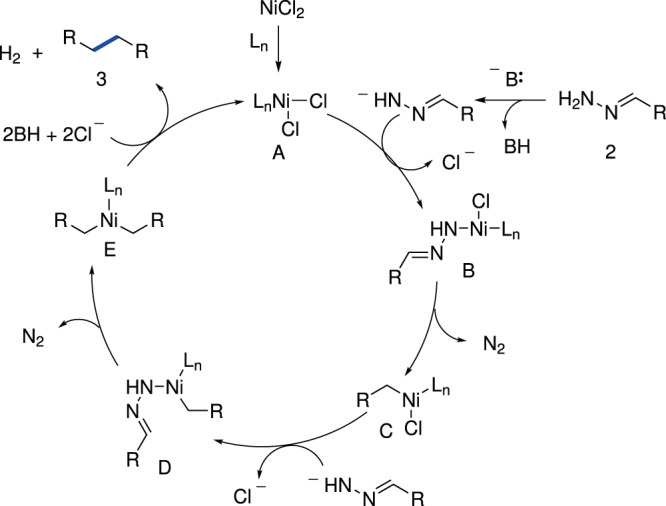
Proposed mechanism. Possible reaction mechanism of deoxygenative homo-coupling of aldehydes/ ketones. L_n_ means ligand.

Based on the above experimental results and our previously reported works^34,35^, we proposed a plausible reaction mechanism, as depicted in Fig. 5. Initially, the active nickel (II) species **A** potentially coordinates with hydrazone anion to form nickel (II) complex **B**, which rapidly releases N_2_ gas through Wolff−Kishner reaction-like process and an intramolecular benzyl migration to obtain intermediate **C**^35^. Next, intermediate **C** combines with another hydrazone anion to form the intermediate **E** via the nickel (II) complex **D**. Finally, reductive elimination and hydrogen generation provides the desired homo-coupling product **3** and regenerates the active catalyst. It is also possible to combine the active nickel species **A** with two molecules of hydrazone anions, followed by intramolecular migration, deprotonation and release of N_2_ to obtain the desired product. More explorations of mechanistic details of this protocol are undergoing in our laboratory.

## Discussion

In conclusion, a direct deoxygenative homo-coupling of hydrazones generated in situ from naturally abundant aldehydes or ketones to construct C(sp^3^)−C(sp^3^) bond has been established, catalyzed by earth-abundant nickel. This protocol is under mild reaction conditions with a broad substrate scope bearing a wide range of functional groups. Furthermore, this homo-coupling protocol can be applied to the rapid synthesis of some complex molecules such as Brittonin A and the transformation of PEEK model substrates. Most importantly, this hydrazine-mediated homogeneous catalytic process enables the preparation of high value-added bibenzyl derivatives from simple and readily available raw materials and provides a useful alternative for C(sp^3^)−C(sp^3^) bond formation.

## Methods

### General procedure for reactions in Table 1

A flame-dried V-shape reaction vial (10 cm^3^) equipped with a magnetic stir bar was transferred into the glovebox and was charged with catalyst, ligand, and base, hydrazones (2 × 0.1 mmol) and solvent (1 mL). Then, the reaction vessel was sealed, moved out of the glovebox, and placed in a preheated oil bath. The mixture was stirred under an argon atmosphere. After completion of the reaction, the reaction mixture was cooled to room temperature and concentrated, and then dibromomethane (7 µL, 0.1 mmol) was added into the mixture as standard. The crude mixture was diluted by CDCl_3_ to run the ^1^H NMR test to determine the ^1^H NMR yield.

### General procedure for reactions in Figs. 2, 3 and 4

A flame-dried V-shape reaction vial (10 cm^3^) equipped with a magnetic stir bar was charged with NiCl_2_ (3.9 mg, 30 mol%), IMes∙HCl (10.2 mg, 30 mol%) and hydrazones (2 × 0.1 mmol). The vial was transferred into the glovebox and charged with 1,4-dioxane (1 mL) and DBU (33 µL, 0.22 mmol) before being sealed with a rubber septum. The tube was placed in a preheated oil bath at 100 °C and the mixture was stirred under an argon atmosphere for 24–36 h. The reaction mixture was cooled to room temperature and concentrated, and then was purified by preparative TLC on silica gel eluting with hexane: EtOAc (50:1-2:1) to afford the products. (Note: For Figs. 4a, 2 equiv of TEMPO were added into the reaction; for Fig. 4b, replace hydrazone with deuterated hydrazine; for Fig. 4c, replace hydrazone with dibenzalhydrazine; for Fig. 4d, replace hydrazone with (*E*)−1,2-diphenylethene and added N_2_H∙H_2_O (0.2 mmol); for Fig. 4e, replace hydrazone with (*E*)−1,2-diphenylethene and added hydrazone **2b** (0.2 mmol)).

## Supplementary information

Supplementary Information

## Data Availability

The authors declare that the data supporting the findings of this study are available within the article and Supplementary Information file, or from the corresponding author upon reasonable request. The X-ray crystallographic coordinates for structures (compound **9**) reported in this study have been deposited at the Cambridge Crystallographic Data Centre (CCDC), under deposition numbers of CCDC 2062859. These data can be obtained free of charge from The Cambridge Crystallographic Data Centre via www.ccdc.cam.ac.uk/data_request/cif.
