# Supplementary material for: C(sp3)−C(sp3) bond formation via nickel-catalyzed deoxygenative homo-coupling of aldehydes/ketones mediated by hydrazine
Source: Nat Commun. 2021 Jun 17;12:3729. doi: 10.1038/s41467-021-23971-7 (PMC8211713; doi:10.1038/s41467-021-23971-7)
Supplement: Supplementary file 1 — Supplementary Information [file 41467_2021_23971_MOESM1_ESM.pdf]

Supplemental Information

Dawei Cao,[1, 2, 3] Chen-Chen Li,[1] Huiying Zeng,[3] Yong Peng[2] and Chao-Jun Li[1,*]

[1] Department of Chemistry and FQRNT Centre for Green Chemistry and Catalysis, McGill University, 801 Sherbrooke St. West, Montreal, QC H3A 0B8, Canada.

[2] Key Laboratory of Magnetism and Magnetic Materials of the Ministry of Education, Lanzhou University, Lanzhou 730000, P. R. China.

[3] The State Key Laboratory of Applied Organic Chemistry, Lanzhou University, Lanzhou 730000, P. R. China.

[*]Corresponding Authors: cj.li@mcgill.ca

Table of Contents

Ⅰ. **Supplementary Note 1** **S2**

Ⅱ. **Supplementary Methods** **S2**

Ⅲ. **Supplementary Note 2** **S3**

i. Characterization of Products **S3**

ii. Deuterium Experiments **S22**

Ⅳ. **Supplementary Figures** **S24**

Ⅴ. **Supplementary References** **S63**

Ⅰ. Supplementary Note 1

General Information: All reagents and solvents were purchased from commercial sources (Alfa, Acros, Aldrich, TCI and Combi-Blocks) and used without further purification unless otherwise stated. ^{1}H and ^{13}C NMR spectra were taken on Agilent 600, Bruker 400 or 500 MHz spectrometers. Chemical shifts of ^{1}H NMR spectra were reported using either residual solvent signal of $CDCl_3$ (δ = 7.26 ppm) or TMS (δ = 0.00 ppm) as internal standard. Chemical shifts of ^{13}C NMR spectra were reported using residual solvent signal of $CDCl_3$ (δ = 77.16 ppm) as internal standard. The peak patterns are indicated as follows: s, singlet; d, doublet; dd, doublet of doublet; t, triplet; q, quartet; m, multiplet. The coupling constants, *J*, are reported in Hertz (Hz). All reactions were monitored by thin-layer chromatography (TLC). Column chromatography was performed on silica gel (200-300 mesh) and visualized with ultraviolet light. EI-MS was obtained from the Agilent GC-MS system. All solvents were purified and dried by standard techniques.

Ⅱ. Supplementary Methods

General procedure for synthesizing aldehyde-derived hydrazones

A round-bottom flask, equipped with dropping funnel, was charged with magnetic stirring bar and hydrazine hydrate (6 mmol, 1.2 equiv, from Aldrich, N_2H_4 64–65 wt%) and then the solution of aldehyde (5 mmol) in methanol (25 mL) was added dropwise. The mixture was stirred at room temperature for 1 h. After the aldehyde was consumed completely, methanol and the extra hydrazine were removed under reduced pressure at room temperature (25 °C). Water (30 mL) was added and the mixture was extracted with dichloromethane (3×20 mL). The combined extracts were washed with brine and dried with anhydrous sodium sulfate. Solvent was removed by rotary evaporation at room temperature (25 °C) to provide the desired hydrazone (> 95%, as shown by ^{1}H NMR), which was used directly without further purification.

General procedure for synthesizing benzophenone-derived hydrazones

To a round-bottom flask charged with magnetic stirring bar, hydrazine hydrate (20 mmol, 10 equiv, from Aldrich, N_2H_4 64–65 wt%) and benzophenone (2 mmol, 1.0 equiv) in ethanol (20 mL) were added. Then HOAc (33 μL) was added and the mixture was heated at reflux for 12 h. After cooling to room temperature, the mixture was then evaporated under reduced pressure. Water (30 mL) was added and the mixture was extracted with dichloromethane (3×20 mL). The combined extracts were washed with brine and dried with anhydrous sodium sulfate. Solvent was removed by rotary evaporation at room temperature (25 °C) to provide the desired hydrazone (> 95%, as shown by ^{1}H NMR), which was used directly without further purification. If the purity of the hydrazone is not enough, the crude hydrazone was purified by a short silica gel flash column chromatography (0.1% Et_3N in hexane: EtOAc (50:1-2:1) to afford the pure products. (Note: For **2ag-2aj**, an inseparable mixture of two isomers of hydrazone was obtained, which was used in the next step without further purification.)

General procedure for synthesizing aryl alkyl ketone-derived hydrazones

To a round-bottom flask charged with magnetic stirring bar, hydrazine hydrate (6 mmol, 3 equiv, from Aldrich, N_2H_4 64–65 wt%) and alkyl ketone (2 mmol, 1 equiv) in ethanol (20 mL) were added, and the mixture was heated at reflux for 3 h. After cooling to room temperature, the mixture was then evaporated under reduced pressure. Water (30 mL) was added and the mixture was extracted with dichloromethane (3×20 mL). The combined extracts were washed with brine and dried with anhydrous

sodium sulfate. Solvent was removed by rotary evaporation at room temperature (25 °C) to provide the desired hydrazone (> 95%, as shown by ^{1}H NMR), which was used directly without further purification (Note: For **2ak-2al**, an inseparable mixture of two isomers of hydrazone was obtained, and used in the next step without further purification).

General experimental procedure for deoxygenation homo-coupling of aldehydes/ketones

A flame-dried V-shape reaction vial (10 cm^3) equipped with a magnetic stir bar was charged with $NiCl_2$ (3.9 mg, 30 mol%), IMes·HCl (10.2 mg, 30 mol%) and solid materials (2 × 0.1 mmol). The vial was transferred into the glovebox and charged with 1,4-dioxane (1 mL), liquid materials (2 × 0.1 mmol) and DBU (33 µl, 0.22 mmol) before being sealed with a rubber septum. The tube was placed in a preheated oil bath at 100 ºC and the mixture was stirred under an argon atmosphere for 24 h. The reaction mixture was cooled to room temperatures and concentrated, and then was purified by preparative TLC on silica gel eluting with hexane: EtOAc (50:1-2:1) to afford the products.

Ⅲ. Supplementary Note 2

i. Characterization of Products

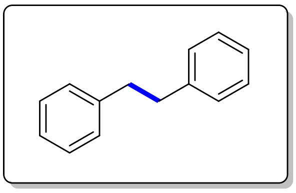

1,2-Diphenylethane (CAS: 103-29-7) [1]

Yield: 83% (15.1 mg);

Yield: 75% (1.09 g) gram-scale experiment.

^{1}H NMR ($CDCl_3$, 400 MHz) δ: 7.33 (t, *J* = 7.5 Hz, 4H), 7.24 (t, *J* = 7.2 Hz, 6H), 2.97 (s, 4H).

^{13}C NMR ($CDCl_3$, 101 MHz) δ: 141.8, 128.4, 128.3, 125.9, 37.9.

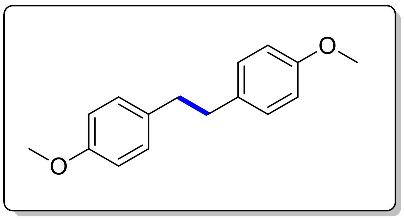

1,2-Bis(4-methoxyphenyl)ethane (CAS: 1657-55-2) [1]

Yield: 84% (20.4 mg)

^{1}H NMR ($CDCl_3$, 400 MHz) δ: 7.11 (d, *J* = 8.6 Hz, 4H), 6.85 (d, *J* = 8.7 Hz, 4H), 3.82 (s, 6H), 2.86 (s, 4H).

^{13}C NMR ($CDCl_3$, 101 MHz) δ: 157.8, 134.0, 129.3, 113.7, 55.2, 37.3.

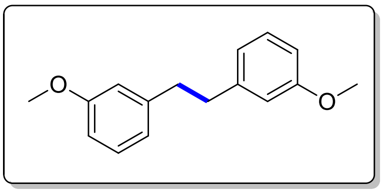

1,2-Bis(3-methoxyphenyl)ethane (CAS: 36707-27-4) [1]

Yield: 86% (20.8 mg)

^{1}H NMR ($CDCl_3$, 400 MHz) δ: 7.23 (t, *J* = 7.6 Hz, 2H), 6.83 – 6.75 (m, 6H), 3.81 (s, 6H), 2.92 (s, 4H).

^{13}C NMR ($CDCl_3$, 101 MHz) δ: 159.6, 143.4, 129.3, 120.8, 114.2, 111.3, 55.1, 37.8.

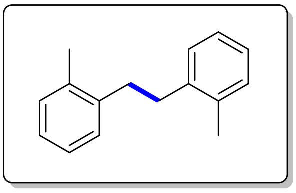

1,2-Di-*o*-tolylethane (CAS: 952-80-7) [1]

Yield: 73% (15.3 mg)

^{1}H NMR ($CDCl_3$, 400 MHz) δ: 7.21 – 7.17 (m, 8H), 2.90 (s, 4H), 2.37 (s, 6H).

^{13}C NMR ($CDCl_3$, 101 MHz) δ: 140.2, 135.9, 130.2, 128.8, 126.1, 126.0, 34.1, 19.3.

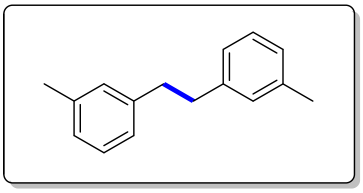

1,2-Di-*m*-tolylethane (CAS: 4662-96-8) [1]

Yield: 79% (16.6 mg)

^{1}H NMR ($CDCl_3$, 400 MHz) δ: 7.21 (t, *J* = 7.5 Hz, 2H), 7.08 – 7.01 (m, 6H), 2.90 (s, 4H), 2.36 (s, 6H).

^{13}C NMR ($CDCl_3$, 101 MHz) δ: 141.9, 137.0, 129.2, 128.2, 126.6, 125.4, 38.0, 21.4.

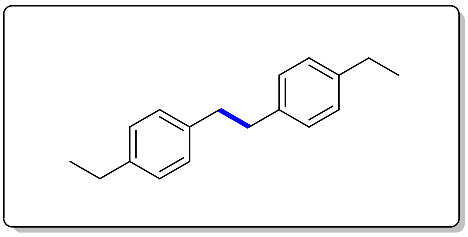

1,2-Bis(4-ethylphenyl)ethane (CAS: 51526-06-8) [2]

Yield: 82% (19.5 mg)

^{1}H NMR ($CDCl_3$, 400 MHz) δ: 7.18 (s, 8H), 2.92 (s, 4H), 2.68 (d, *J* = 7.6 Hz, 4H), 1.28 (dt, *J* = 7.6, 3.8 Hz, 6H).

^{13}C NMR ($CDCl_3$, 101 MHz) δ: 141.7, 139.2, 128.3, 127.8, 37.6, 28.5, 15.7.

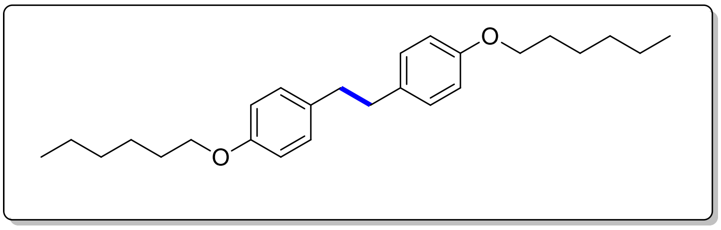

1,2-Bis(4-(hexyloxy)phenyl)ethane (CAS: 1043905-65-2) [, 3]

Yield: 82% (31.4 mg)

^{1}H NMR ($CDCl_3$, 400 MHz) δ: 7.10 (d, *J* = 8.3 Hz, 4H), 6.85 (d, *J* = 8.3 Hz, 4H), 3.96 (t, *J* = 6.5 Hz, 4H), 2.85 (s, 4H), 1.84 – 1.76 (m, 4H), 1.48 (dd, *J* = 13.8, 6.5 Hz, 4H), 1.42 – 1.35 (m, 8H), 0.95 (t, *J* = 6.4 Hz, 6H).

^{13}C NMR (CDCl$_3$, 101 MHz) δ: 157.3, 133.8, 129.3, 114.3, 68.0, 37.3, 31.6, 29.3, 25.8, 22.6, 14.0.

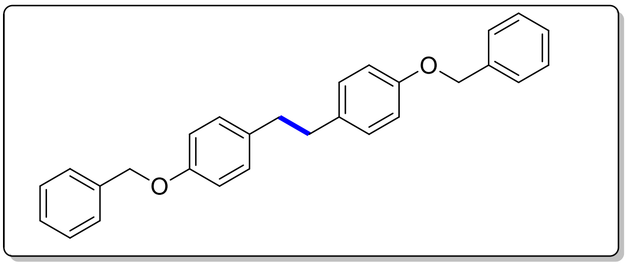

1,2-Bis(4-(benzyloxy)phenyl)ethane (CAS: 81625-34-5) [4]

Yield: 85% (33.6 mg)

^{1}H NMR (CDCl$_3$, 400 MHz) δ: 7.46 – 7.35 (m, 10H), 7.11 (d, *J* = 8.4 Hz, 4H), 6.92 (d, *J* = 8.4 Hz, 4H), 5.08 (s, 4H), 2.86 (s, 4H).

^{13}C NMR (CDCl$_3$, 101 MHz) δ: 157.0, 137.2, 134.3, 129.4, 128.5, 127.9, 127.5, 114.7, 70.1, 37.3.

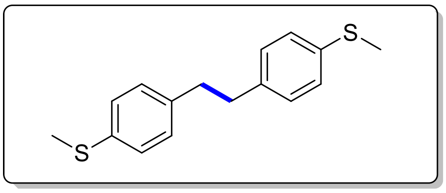

1,2-Bis(4-(methylthio)phenyl)ethane (CAS: 61812-41-7) [5]

Yield: 72% (19.7 mg)

^{1}H NMR (CDCl$_3$, 400 MHz) δ: 7.21 (d, *J* = 8.0 Hz, 4H), 7.11 (d, *J* = 8.0 Hz, 4H), 2.88 (s, 4H), 2.50 (s, 6H).

^{13}C NMR (CDCl$_3$, 101 MHz) δ: 138.7, 135.5, 129.0, 127.1, 37.3, 16.3.

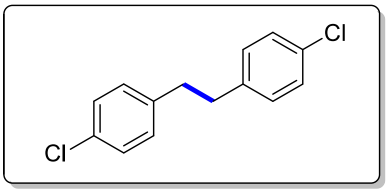

1,2-Bis(4-chlorophenyl)ethane (CAS: 5216-35-3) [5]

Yield: 75% (18.8 mg)

^{1}H NMR (CDCl$_3$, 400 MHz) δ: 7.26 (d, *J* = 8.2 Hz, 4H), 7.08 (d, *J* = 8.2 Hz, 4H), 2.89 (s, 4H).

^{13}C NMR (CDCl$_3$, 101 MHz) δ: 139.6, 131.8, 129.8, 128.4, 37.00.

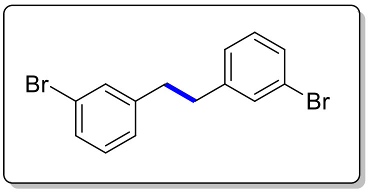

1,2-Bis(3-bromophenyl)ethane (CAS: 82732-03-4) [6]

Yield: 66% (22.4mg)

^{1}H NMR (CDCl$_3$, 400 MHz) δ: 7.31 (d, *J* = 8.6 Hz, 4H), 7.12 (t, *J* = 7.6 Hz, 2H), 7.03 (d, *J* = 7.6 Hz, 2H), 2.84 (s, 4H).

^{13}C NMR (CDCl$_3$, 101 MHz) δ: 143.4, 131.5, 129.9, 129.2, 127.1, 122.4, 37.2.

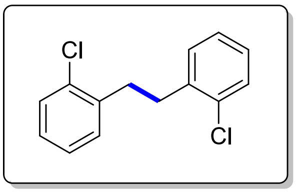

1,2-Bis(2-chlorophenyl)ethane (CAS: 6639-40-3) [7]

Yield: 63% (15.8 mg)

^{1}H NMR (CDCl$_3$, 400 MHz) δ: 7.41 – 7.37 (m, 2H), 7.21 – 7.17 (m, 6H), 3.07 (s, 4H).

^{13}C NMR (CDCl$_3$, 101 MHz) δ: 138.9, 134.0, 130.6, 129.4, 127.5, 126.7, 33.8.

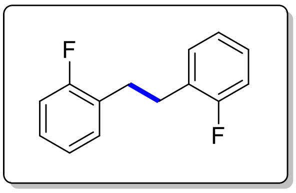

1,2-Bis(2-fluorophenyl)ethane (CAS: 349-38-2) [1]

Yield: 76% (16.6 mg)

^{1}H NMR (CDCl$_3$, 400 MHz) δ: 7.22 – 7.13 (m, 4H), 7.09 – 6.99 (m, 4H), 2.98 (s, 4H).

^{13}C NMR (CDCl$_3$, 101 MHz) δ: 161.17 (d, J^F = 244.8 Hz), 130.70 (d, J^F = 4.9 Hz), 128.23 (d, J^F = 15.5 Hz), 127.75 (d, J^F = 8.1 Hz), 123.86 (d, J^F = 3.3 Hz), 115.17 (d, J^F = 22.2 Hz), 29.71.

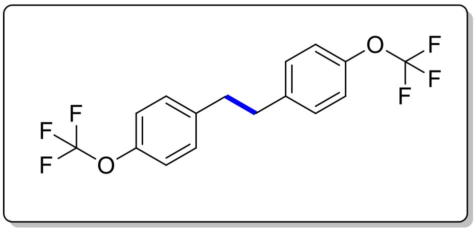

1,2-Bis(4-(trifluoromethoxy)phenyl)ethane (CAS: 1384576-14-0) [8]

Yield: 38% (13.3 mg)

^{1}H NMR (CDCl$_3$, 400 MHz) δ: 7.19 – 7.13 (m, 8H), 2.94 (s, 4H).

^{13}C NMR (CDCl$_3$, 101 MHz) δ: 147.6, 139.9, 129.7, 121.0, 120.5 (q, J^F= 207.1 Hz), 37.0.

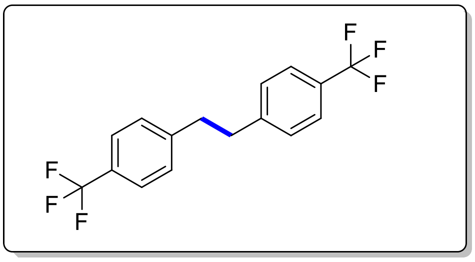

1,2-Bis(4-(trifluoromethyl)phenyl)ethane (CAS: 42134-71-4) [8]

Yield: 46% (14.6 mg)

^{1}H NMR (CDCl$_3$, 400 MHz) δ: 7.53 (d, J = 7.8 Hz, 4H), 7.25 (d, J = 7.4 Hz, 4H), 2.99 (s, 4H).

^{13}C NMR (CDCl$_3$, 101 MHz) δ: 145.0, 128.8, 128.5 (q, J^F= 31.5 Hz), 125.4 (q, J^F= 2.0 Hz), 124.3(q, J^F= 272.2 Hz), 37.2.

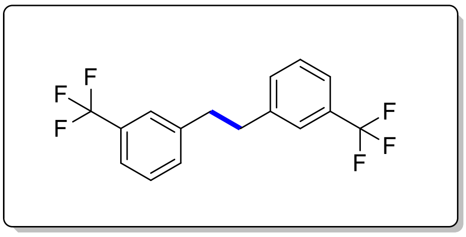

1,2-Bis(3-(trifluoromethyl)phenyl)ethane (CAS: 72390-22-8) [9]

Yield: 53% (16.9 mg)

^{1}H NMR ($CDCl_3$, 400 MHz) δ: 7.50 (d, J = 7.8 Hz, 2H), 7.42 (dd, J = 8.7, 6.3 Hz, 4H), 7.34 (d, J = 7.6 Hz, 2H), 3.02 (s, 4H).

^{13}C NMR ($CDCl_3$, 101 MHz) δ: 141.8, 131.9, 130.7 (q, J^F = 32.3 Hz), 128.8, 125.2 (q, J^F = 4.0 Hz), 124.2 (q, J^F = 283.8 Hz), 123.1 (q, J^F = 4.0 Hz), 37.4.

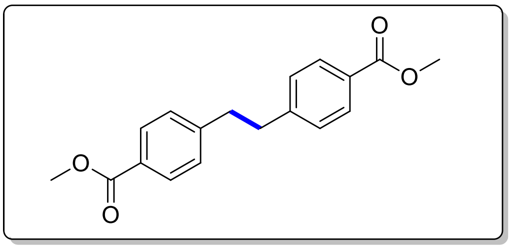

Dimethyl 4,4'-(ethane-1,2-diyl)dibenzoate (CAS: 797-21-7) [10]

Yield: 42% (12.5 mg)

^{1}H NMR ($CDCl_3$, 400 MHz) δ: 7.96 (d, J = 6.9 Hz, 4H), 7.22 (d, J = 7.0 Hz, 4H), 3.92 (s, 6H), 3.01 (s, 4H).

^{13}C NMR ($CDCl_3$, 101 MHz) δ: 167.0, 146.5, 129.7, 128.5, 128.1, 52.0, 37.4.

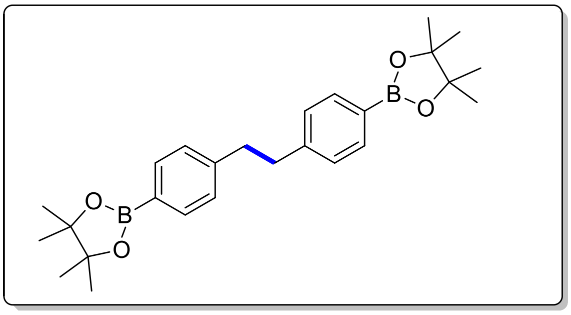

1,2-Bis(4-(4,4,5,5-tetramethyl-1,3,2-dioxaborolan-2-yl)phenyl)ethane (CAS: 1422172-96-0) [11]

Yield: 78% (33.9 mg)

^{1}H NMR ($CDCl_3$, 400 MHz) δ: 7.75 (d, J = 8.0 Hz, 4H), 7.21 (d, J = 8.0 Hz, 4H), 2.95 (s, 4H), 1.37 (s, 24H).

^{13}C NMR ($CDCl_3$, 101 MHz) δ: 145.0, 134.9, 127.9, 83.6, 38.0, 24.9.

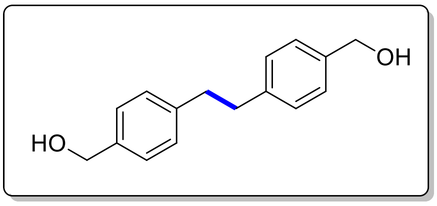

(Ethane-1,2-diylbis(4,1-phenylene))dimethanol (CAS: 88579-94-6) [12]

Yield: 52% (12.6 mg)

^{1}H NMR ($CDCl_3$, 400 MHz) δ: 7.31 (d, *J* = 8.0 Hz, 4H), 7.20 (d, *J* = 8.0 Hz, 4H), 4.69 (s, 4H), 2.94 (s, 4H), 1.61 (s, 2H).

^{13}C NMR ($CDCl_3$, 101 MHz) δ: 141.2, 138.5, 128.7, 127.2, 65.3, 37.6.

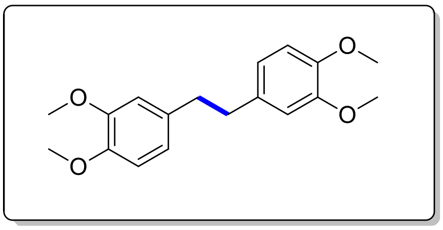

1,2-Bis(3,4-dimethoxyphenyl)ethane (CAS: 5963-51-9) [13]

Yield: 86% (26.0 mg)

^{1}H NMR ($CDCl_3$, 400 MHz) δ: 6.81 (d, *J* = 8.1 Hz, 2H), 6.73 (dd, *J* = 8.2, 1.5 Hz, 2H), 6.68 (d, *J* = 1.4 Hz, 2H), 3.88 (s, 6H), 3.86 (s, 6H), 2.87 (s, 4H).

^{13}C NMR ($CDCl_3$, 101 MHz) δ: 148.7, 147.2, 134.4, 120.3, 111.9, 111.1, 55.9, 55.8, 37.7.

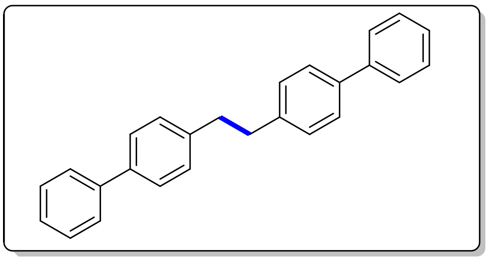

1,2-Di([1,1'-biphenyl]-4-yl)ethane (CAS: 1694-23-1) [14]

Yield: 76% (25.4 mg)

^{1}H NMR ($CDCl_3$, 400 MHz) δ: 7.62 (d, *J* = 7.4 Hz, 4H), 7.56 (d, *J* = 8.0 Hz, 4H), 7.46 (t, *J* = 7.6 Hz, 4H), 7.37 (t, *J* = 7.4 Hz, 2H), 7.33 (t, *J* = 6.4 Hz, 4H), 3.04 s, 4H).

^{13}C NMR ($CDCl_3$, 101 MHz) δ: 141.0, 140.8, 138.9, 128.9, 128.7, 127.1, 127.0 (2C), 37.5.

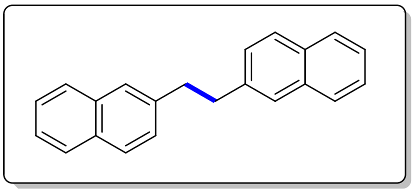

1,2-Di(naphthalen-2-yl)ethane (CAS: 21969-45-9) [10]

Yield: 78% (22.0 mg)

^{1}H NMR ($CDCl_3$, 400 MHz) δ: 7.81 (ddd, *J* = 9.1, 7.9, 5.2 Hz, 6H), 7.68 (s, 2H), 7.49 – 7.44 (m, 4H), 7.39 (dd, *J* = 8.4, 1.6 Hz, 2H), 3.21 (s, 4H).

^{13}C NMR ($CDCl_3$, 101 MHz) δ: 139.3, 133.6, 132.1, 127.9, 127.6, 127.5, 127.3, 126.5, 125.9, 125.2, 38.0.

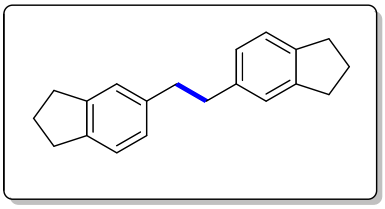

1,2-Bis(2,3-dihydro-1H-inden-5-yl)ethane (CAS: 87073-79-8) [15]

Yield: 82% (21.5 mg)

^{1}H NMR (CDCl$_3$, 400 MHz) δ: 7.19 (d, *J* = 7.6 Hz, 2H), 7.15 (s, 2H), 7.04 (d, *J* = 7.5 Hz, 2H), 2.95 – 2.90 (m, 12H), 2.11 (dd, *J* = 9.4, 5.4 Hz, 4H).

^{13}C NMR (CDCl$_3$, 101 MHz) δ: 144.4, 141.7, 140.0, 126.1, 124.4, 124.2, 38.3, 32.8, 32.5, 25.5.

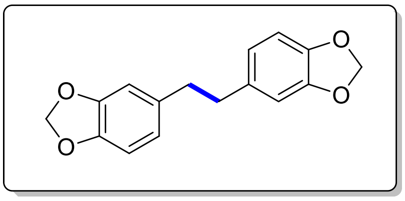

1,2-Bis(benzo[d][1,3]dioxol-5-yl)ethane (CAS: 80784-19-6) [1]

Yield: 90% (24.3 mg)

^{1}H NMR (CDCl$_3$, 400 MHz) δ: 6.74 (d, *J* = 7.9 Hz, 2H), 6.68 (d, *J* = 1.5 Hz, 2H), 6.63 (d, *J* = 1.5 Hz, 2H), 5.95 (s, 4H), 2.81 (s, 4H).

^{13}C NMR (CDCl$_3$, 101 MHz) δ: 147.5, 145.6, 135.5, 121.2, 108.9, 108.1, 100.7, 37.9.

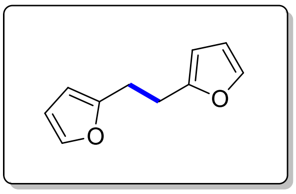

1,2-Di(furan-2-yl)ethane (CAS: 36707-31-0) [16]

Yield: 78% (12.6 mg)

^{1}H NMR (CDCl$_3$, 400 MHz) δ: 7.34 (d, *J* = 1.0 Hz, 2H), 6.30 (dd, *J* = 3.0, 1.9 Hz, 2H), 6.02 (d, *J* = 3.1 Hz, 2H), 3.00 (s, 4H).

^{13}C NMR (CDCl$_3$, 101 MHz) δ: 154.9, 141.0, 110.1, 105.2, 26.7.

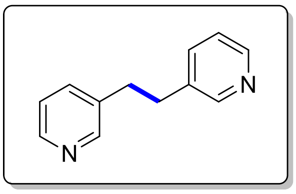

1,2-Di(pyridin-3-yl)ethane (CAS: 4916-58-9) [10]

Yield: 49% (9.0 mg)

^{1}H NMR (CDCl$_3$, 500 MHz) δ: 8.49 (dd, *J* = 4.8, 1.5 Hz, 2H), 8.45 (d, *J* = 1.9 Hz, 2H), 7.47 – 7.44 (m, 2H), 7.23 (dd, *J* = 7.5, 5.0 Hz, 2H), 2.97 (s, 4H).

^{13}C NMR (CDCl$_3$, 126 MHz) δ: 150.0, 147.8, 135.9, 123.3, 34.5.

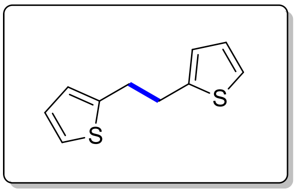

1,2-Di(thiophen-2-yl)ethane (CAS: 7326-80-9) [17]

Yield: 70% (13.4 mg)

^{1}H NMR (CDCl$_3$, 400 MHz) δ: 7.16 (dd, *J* = 5.1, 1.1 Hz, 2H), 6.95 (dd, *J* = 5.1, 3.4 Hz, 2H), 6.83 (d, *J* = 3.2 Hz, 2H), 3.23 (s, 4H).

^{13}**C NMR (CDCl$_3$, 101 MHz)** δ: 143.7, 126.7, 124.6, 123.3, 32.1.

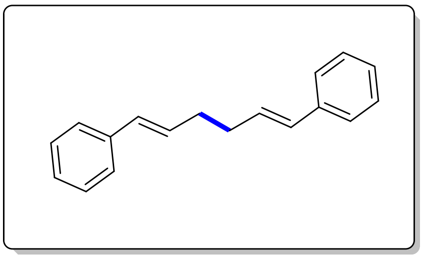

(1E,5E)-1,6-Diphenylhexa-1,5-diene (CAS: 4439-45-6) [18]

Yield: 46% (10.8 mg)

^{1}**H NMR (CDCl$_3$, 400 MHz)** δ: 7.40 (d, *J* = 7.8 Hz, 4H), 7.34 (t, *J* = 7.5 Hz, 4H), 7.25 (t, *J* = 7.2 Hz, 2H), 6.49 (d, *J* = 15.8 Hz, 2H), 6.32 (dt, *J* = 15.7, 6.3 Hz, 2H), 2.47 – 2.43 (m, 4H).

^{13}**C NMR (CDCl$_3$, 101 MHz)** δ: 137.7, 130.4, 130.0, 128.5, 126.9, 126.0, 32.9.

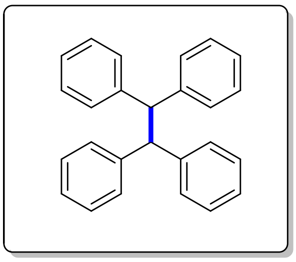

1,1,2,2-Tetraphenylethane (CAS: 632-50-8) [19]

Yield: 80% (26.7 mg)

^{1}**H NMR (CDCl$_3$, 400 MHz)** δ: 7.19 (d, *J* = 7.7 Hz, 8H), 7.13 (t, *J* = 7.6 Hz, 8H), 7.04 (t, *J* = 7.2 Hz, 4H), 4.80 (s, 2H).

^{13}**C NMR (CDCl$_3$, 101 MHz)** δ: 143.4, 128.5, 128.1, 125.8, 56.3.

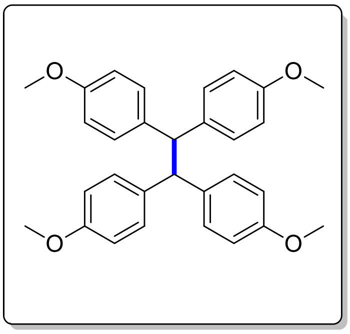

1,1,2,2-Tetrakis(4-methoxyphenyl)ethane (CAS: 51048-43-2) [19]

Yield: 84% (38.1 mg)

^{1}**H NMR (CDCl$_3$, 400 MHz)** δ: 7.05 (d, *J* = 8.6 Hz, 8H), 6.67 (d, *J* = 8.6 Hz, 8H), 4.60 (s, 2H), 3.71 (s, 12H).

^{13}**C NMR (CDCl$_3$, 101 MHz)** δ: 157.4, 136.3, 129.3, 113.5, 55.1, 54.9.

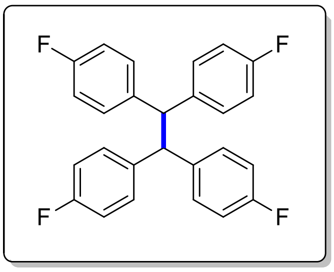

1,1,2,2-Tetrakis(4-fluorophenyl)ethane (CAS: 19422-37-8) [20]

Yield: 82% (33.3 mg)

^{1}H NMR (CDCl$_3$, 400 MHz) δ: 7.07 (dd, *J* = 8.5, 5.4 Hz, 8H), 6.85 (t, *J* = 8.6 Hz, 8H), 4.65 (s, 2H).

^{13}C NMR (CDCl$_3$, 101 MHz) δ: 161.1 (d, J^F = 245.2 Hz), 138.6 (d, J^F = 2.9 Hz), 129.7 (d, J^F = 7.9 Hz), 115.2 (d, J^F = 21.3 Hz), 55.09 (s).

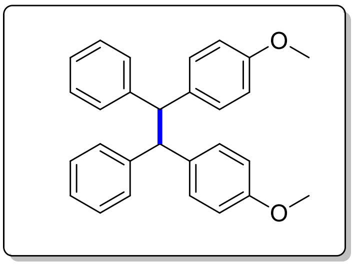

1,2-Bis(4-methoxyphenyl)-1,2-diphenylethane (CAS: 97075-57-5) [21]

Yield: 83% (32.7 mg)

^{1}H NMR (CDCl$_3$, 400 MHz) δ: 7.18 – 7.01 (m, 16H), 6.68 (dd, *J* = 12.1, 5.4 Hz, 4H), 4.70 (s, 2H), 3.71 (d, J = 6.2 Hz, 6H).

^{13}C NMR (CDCl$_3$, 101 MHz) δ: 157.5, 144.0, 143.9, 135.8, 129.4 (2C), 128.4 (2C), 128.1(2C), 125.7, 113.(2C), 55.6, 55.1, 55.0.

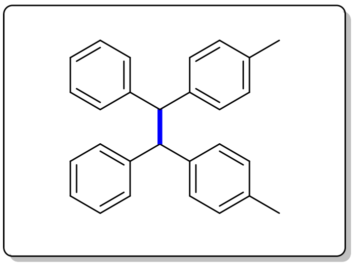

1,2-Diphenyl-1,2-di-p-tolylethane (CAS: 88689-63-8) [19]

Yield: 84% (30.4 mg)

^{1}H NMR (CDCl$_3$, 400 MHz) δ: 1H NMR (400 MHz, CDCl3) δ 7.20 – 6.98 (m, 18H), 4.79 (s, 2H), 2.25 (s, 6H).

^{13}C NMR (CDCl$_3$, 101 MHz) δ: 144.0, 140.5, 135.1, 128.9, 128.4, 128.3, 128.0, 125.6, 55.9, 20.9.

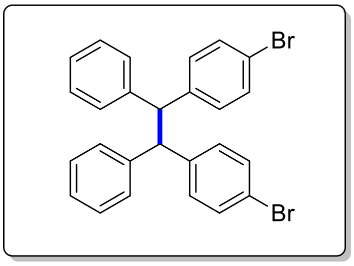

1,2-Bis(4-bromophenyl)-1,2-diphenylethane (CAS: 22524-18-1) [19]

Yield: 50% (24.6 mg)

^{1}H NMR ($CDCl_3$, 400 MHz) δ: 7.30 – 6.97 (m, 18H), 4.68 (d, *J* = 4.1 Hz, 2H).

^{13}C NMR ($CDCl_3$, 101 MHz) δ: 143.1 (2C), 141.5 (2C), 132.5, 132.2, 130.4(2C), 130.3, 128.7, 128.5, 128.2(2C), 127.6(2C), 126.8, 126.6, 55.2(2C).

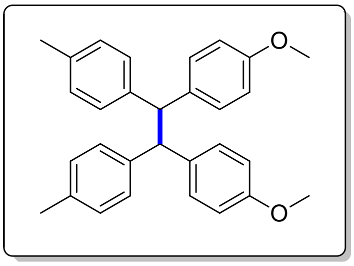

1,2-Bis(4-methoxyphenyl)-1,2-di-p-tolylethane (CAS: 210624-67-2) [22]

Yield: 83% (35.0 mg)

^{1}H NMR ($CDCl_3$, 400 MHz) δ: 7.08 (dd, *J* = 13.9, 5.3 Hz, 8H), 6.95 (d, *J* = 7.0 Hz, 4H), 6.67 (d, *J* = 7.5 Hz, 4H), 4.67 (s, 2H), 3.71 (s, 6H), 2.22 (s, 6H).

^{13}C NMR ($CDCl_3$, 101 MHz) δ: 157.3, 141.1, 141.0, 136.3 (2C), 134.9, 129.3, 129.2, 128.8, 128.2 (2C), 113.5, 55.1, 55.0, 20.9 (2C).

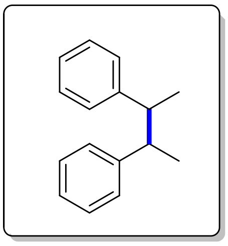

2,3-Diphenylbutane (CAS: 5789-35-5) [23]

Yield: 48% (10.1 mg)

^{1}H NMR ($CDCl_3$, 500 MHz) δ: 7.34 (t, *J* = 7.5 Hz, 4H), 7.25 (d, *J* = 7.5 Hz, 6H), 2.83 (dt, *J* = 6.4, 4.0 Hz, 2H), 1.05 (d, *J* = 6.4 Hz, 6H).

^{13}C NMR ($CDCl_3$, 126 MHz) δ: 146.5, 128.3, 127.6, 126.0, 47.3, 21.0.

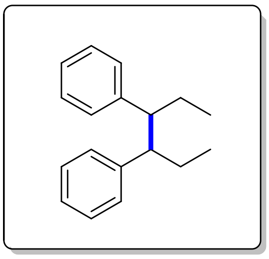

3,4-Diphenylhexane (CAS: 5789-31-1) [13]

Yield: 43% (10.2 mg)

^{1}H NMR ($CDCl_3$, 400 MHz) δ: 7.36 (t, *J* = 7.5 Hz, 4H), 7.33-7.11 (m, 16H), 6.91 (d, *J* = 7.7 Hz, 4H), 2.81 – 2.72 (m, 2H), 2.66 – 2.59 (m, 2H), 1.95 – 1.90 (m, 2H), 1.65 (dtd, J = 14.8, 7.4, 2.4 Hz, 2H), 1.47 – 1.34 (m, 4H), 0.78 (t, J = 7.3 Hz, 6H), 0.57 (t, J = 7.3 Hz, 6H).

^{13}C NMR ($CDCl_3$, 101 MHz) δ: 144.5, 143.0, 129.0, 128.4, 128.2, 127.4, 126.0, 125.6, 54.24, 53.25, 27.4, 25.8, 12.4, 12.2.

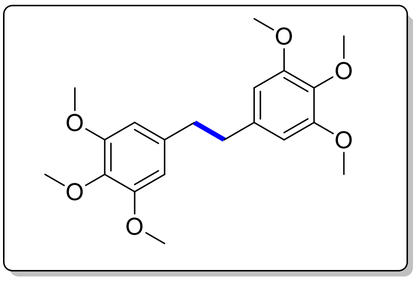

1,2-Bis(3,4,5-trimethoxyphenyl)ethane (CAS: 33284-74-1) [1]

Yield: 76% (27.6 mg)

^{1}H NMR ($CDCl_3$, 400 MHz) δ: 6.39 (s, 4H), 3.85 (s, 18H), 2.87 (s, 4H).

^{13}C NMR ($CDCl_3$, 101 MHz) δ: 153.0, 137.3, 136.2, 105.5, 60.9, 56.1, 38.4.

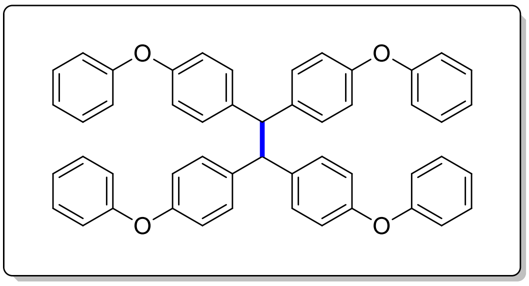

1,1,2,2-Tetrakis(4-phenoxyphenyl)ethane

Yield: 67% (47.0 mg)

^{1}H NMR ($CDCl_3$, 400 MHz) δ: 7.34 – 7.29 (m, 8H), 7.14 (d, *J* = 8.5 Hz, 8H), 7.08 (t, *J* = 7.4 Hz, 4H), 6.94 (d, *J* = 7.8 Hz, 8H), 6.84 (d, *J* = 8.5 Hz, 8H), 4.68 (s, 2H).

^{13}C NMR ($CDCl_3$, 101 MHz) δ: 157.3, 155.1, 138.3, 129.7, 129.6, 123.0, 118.8, 118.6, 55.5.

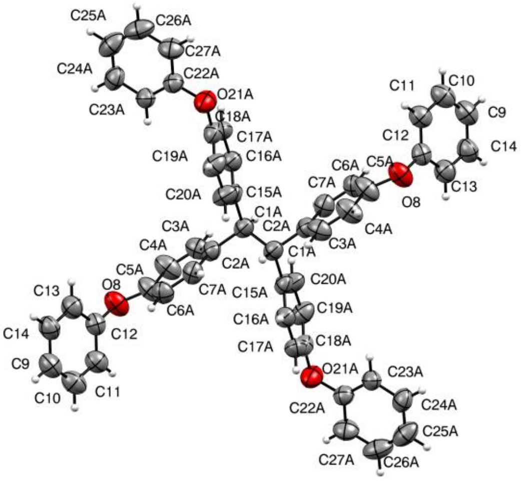

Supplementary Figure 1 X-ray structure of compound 9 (**9-X-ray**, CCDC 2062859)

Supplementary Table 1 Crystal data and structure refinement for 9-X-ray.

Identification code	9-X-ray
Empirical formula	$C_{50}H_{38}O_4$
Formula weight	702.80

Temperature/K	298(2)
Crystal system	monoclinic
Space group	$P2_1/c$
a/Å	18.6957(8)
b/Å	22.1201(8)
c/Å	9.2222(4)
α/°	90
β/°	91.203(3)
γ/°	90
Volume/Å^3	3813.0(3)
Z	4
ρ_{calc}g/cm^3	1.224
μ/mm^{-1}	0.602
F(000)	1480.0
Crystal size/mm^3	0.410 × 0.310 × 0.190
Radiation	CuKα (λ = 1.54178)
2Θ range for data collection/°	4.728 to 145.04
Index ranges	-22 ≤ h ≤ 23, -27 ≤ k ≤ 26, -8 ≤ l ≤ 11
Reflections collected	27891
Independent reflections	7506 [R_{int} = 0.1052, R_{sigma} = 0.0864]
Data/restraints/parameters	7506/710/561
Goodness-of-fit on F^2	0.963
Final R indexes [I>=2σ (I)]	R_1 = 0.0704, wR_2 = 0.1961
Final R indexes [all data]	R_1 = 0.1872, wR_2 = 0.2862
Largest diff. peak/hole / e Å^{-3}	0.16/-0.14

Supplementary Table 2 Fractional Atomic Coordinates (×10^4) and Equivalent Isotropic Displacement Parameters (Å^2×10^3) for 9-X-ray. U_{eq} is defined as 1/3 of of the trace of the orthogonalised U_{IJ} tensor.

**Atom**	***x***	***y***	***z***	**U(eq)**
O21A	-1615.4(18)	6355.5(17)	5301(4)	132.6(12)
O8B	8075(6)	4233(6)	6274(13)	141(4)
O21B	3376.8(17)	3662.7(17)	9552(4)	135.9(12)
C22A	-1351(3)	6560.3(18)	3989(5)	96.0(12)
C16B	4513(2)	4857.5(19)	8102(5)	99.7(13)
C15B	4692(2)	4617.8(17)	6795(4)	91.4(11)
C15A	-322(2)	5380.2(17)	8188(4)	93.3(12)
C1A	151(3)	5014.4(17)	9234(4)	96.6(12)
C22B	3622(3)	3457.0(19)	10883(5)	100.7(13)
C1B	5159(3)	4982.2(17)	5776(4)	93.1(12)
C23A	-641(3)	6606.7(18)	3742(5)	99.2(12)

C16A	-502(3)	5146.2(19)	6855(5)	102.8(13)
C3B	6156(3)	4253.3(18)	5111(5)	102.2(13)
C2B	5934(2)	4773.4(17)	5810(4)	88.8(11)
C2A	935(3)	5211.8(18)	9242(4)	94.6(12)
C18B	3847(2)	3974(2)	8661(5)	105.3(13)
C27B	4324(3)	3386.5(18)	11236(5)	103.8(13)
C17B	4089(3)	4537(2)	9053(5)	111.0(14)
C18A	-1158(2)	6038(2)	6245(6)	102.9(13)
C7A	1165(3)	5736.5(18)	9919(5)	106.1(14)
C4B	6864(3)	4074(2)	5168(5)	115.7(15)
C6A	1876(3)	5909(2)	9878(5)	113.7(15)
C20A	-569(3)	5948(2)	8509(5)	119.5(16)
C19A	-983(3)	6280(2)	7548(6)	122.7(16)
C20B	4441(3)	4049(2)	6451(5)	117.9(16)
C17A	-922(3)	5471(2)	5886(5)	110.0(14)
C7B	6447(3)	5093.6(19)	6569(5)	111.5(14)
C19B	4019(3)	3728(2)	7378(6)	119.2(16)
C27A	-1858(3)	6743(2)	2980(7)	127.3(17)
C5B	7354(3)	4397(2)	5948(6)	120.4(16)
C24A	-436(3)	6848(2)	2450(6)	122.2(16)
C3A	1433(3)	4888(2)	8489(5)	114.8(15)
C25B	4023(5)	2975(2)	13506(7)	151(2)
C23B	3111(3)	3301(2)	11876(7)	136.6(19)
C6B	7145(3)	4918(2)	6658(6)	128.3(18)
C26B	4515(3)	3144(2)	12541(6)	132.9(18)
C26A	-1629(4)	6987(3)	1688(7)	147(2)
C4A	2130(3)	5059(2)	8421(7)	133.4(19)
C5A	2348(3)	5577(2)	9119(7)	129.8(18)
C25A	-925(4)	7032(2)	1446(7)	141(2)
C24B	3317(4)	3054(3)	13192(8)	158(3)
C14B	8191(6)	3189(5)	6243(6)	107(3)
C9B	8321(4)	3739(3)	5569(11)	86(2)
C10B	8742(5)	3761(4)	4347(8)	104(3)
C12B	9034(5)	3232(6)	3798(9)	119(3)
C13B	8904(6)	2682(4)	4472(15)	124(3)
C11B	8482(7)	2661(2)	5694(12)	120(3)
O8C	8046(11)	4340(8)	5440(30)	142(7)
C9C	8391(12)	3834(5)	4910(30)	135(6)
C10C	8151(10)	3473(14)	6033(19)	134(6)
C11C	8388(9)	2880(13)	6164(18)	134(6)

C12C	8866(10)	2647(5)	5170(30)	123(6)
C13C	9106(10)	3009(10)	4050(16)	124(6)
C14C	8869(11)	3602(9)	3920(20)	130(6)
C1	3321(9)	6193(5)	9890(30)	116(4)
C2	3085(8)	6581(10)	8797(16)	120(5)
C3	3368(9)	7160(9)	8702(17)	123(5)
C4	3887(10)	7352(6)	9700(20)	110(4)
C5	4123(10)	6964(9)	10797(17)	116(3)
C6	3840(11)	6384(8)	10890(20)	122(5)
O7	3031(7)	5642(6)	9720(20)	121(4)
O8	3076(3)	5744(4)	8792(15)	128(3)
C9	3928(6)	7336(4)	10404(15)	133(4)
C10	4072(6)	6793(5)	11113(11)	137(3)
C11	3780(6)	6258(3)	10582(9)	116(3)
C12	3344(4)	6264(3)	9343(10)	99(2)
C13	3200(6)	6807(5)	8635(7)	120(3)
C14	3492(7)	7343(3)	9165(12)	127(3)

Supplementary Table 3 Anisotropic Displacement Parameters (Å^2×10^3) for 9-X-ray. The Anisotropic displacement factor exponent takes the form: -2π^2[h^{2}a*^{2}U$_{11}$+2hka*b*U$_{12}$+…].

**Atom**	**U_{11}**	**U_{22}**	**U_{33}**	**U_{23}**	**U_{13}**	**U_{12}**
O21A	102(2)	166(3)	129(3)	34(2)	4(2)	17(2)
O8B	111(4)	136(7)	176(8)	-70(6)	-15(5)	14(4)
O21B	102(2)	180(3)	126(3)	36(2)	10.7(19)	-19(2)
C22A	108(3)	82(3)	98(3)	4(2)	-5(2)	6(2)
C16B	124(4)	83(3)	92(3)	-4(2)	9(3)	6(2)
C15B	112(3)	76(2)	87(3)	6(2)	6(2)	9(2)
C15A	124(3)	72(2)	84(3)	9(2)	9(2)	-9(2)
C1A	134(3)	74(2)	82(3)	-4(2)	1(2)	-6(2)
C22B	111(3)	88(3)	104(3)	1(2)	25(3)	-7(3)
C1B	123(3)	70(2)	86(2)	4(2)	13(2)	7(2)
C23A	107(3)	89(3)	101(3)	7(2)	-7(2)	-10(2)
C16A	127(4)	84(3)	98(3)	-6(2)	1(3)	-4(2)
C3B	122(3)	79(3)	106(3)	-12(2)	-6(3)	6(2)
C2B	110(3)	73(2)	83(3)	2.1(19)	11(2)	3(2)
C2A	120(3)	73(2)	91(3)	3(2)	0(2)	-4(2)
C18B	100(3)	111(3)	105(3)	17(3)	12(3)	0(3)
C27B	121(3)	90(3)	101(3)	6(2)	28(3)	10(3)
C17B	123(4)	119(3)	91(3)	1(3)	13(3)	14(3)
C18A	102(3)	103(3)	104(3)	18(3)	12(3)	7(2)
C7A	132(4)	80(3)	107(3)	-11(2)	20(3)	0(2)

C4B	121(4)	97(3)	129(4)	-29(3)	2(3)	12(3)
C6A	130(4)	95(3)	117(4)	-25(3)	6(3)	-10(3)
C20A	183(5)	87(3)	88(3)	-2(2)	-11(3)	12(3)
C19A	166(5)	99(3)	103(3)	5(3)	11(3)	25(3)
C20B	165(5)	88(3)	102(3)	-6(2)	23(3)	-14(3)
C17A	122(4)	121(3)	86(3)	-3(3)	-4(3)	-13(3)
C7B	114(3)	83(3)	139(4)	-25(3)	16(3)	-3(2)
C19B	142(4)	96(3)	121(4)	-1(3)	13(3)	-20(3)
C27A	115(4)	128(4)	138(4)	2(3)	-20(3)	27(3)
C5B	97(3)	96(3)	168(5)	-31(3)	11(3)	-2(3)
C24A	153(5)	97(3)	118(4)	14(3)	16(3)	-12(3)
C3A	123(4)	84(3)	137(4)	-25(3)	-15(3)	9(3)
C25B	259(7)	96(3)	100(4)	10(3)	13(5)	-30(5)
C23B	131(4)	143(4)	138(4)	-15(3)	40(4)	-39(3)
C6B	106(3)	108(4)	171(5)	-51(3)	11(3)	-7(3)
C26B	175(5)	106(4)	118(4)	14(3)	7(4)	16(3)
C26A	206(6)	124(4)	110(4)	-5(3)	-38(4)	47(5)
C4A	112(4)	105(4)	183(5)	-48(3)	-7(4)	12(3)
C5A	101(4)	100(3)	188(5)	-38(3)	-8(3)	4(3)
C25A	220(6)	97(3)	106(4)	12(3)	12(4)	27(5)
C24B	226(7)	139(5)	111(4)	-11(3)	57(5)	-67(5)
C14B	138(7)	66(5)	117(6)	-5(4)	18(5)	9(5)
C9B	87(5)	82(4)	91(5)	-4(4)	15(4)	-9(3)
C10B	99(6)	111(6)	102(5)	-20(4)	17(4)	-8(5)
C12B	111(7)	122(8)	125(7)	-26(5)	23(5)	10(6)
C13B	120(7)	128(6)	125(8)	-12(6)	5(6)	22(6)
C11B	131(8)	97(5)	131(7)	-13(5)	12(6)	31(5)
O8C	110(8)	87(6)	230(20)	-21(10)	44(12)	-13(6)
C9C	129(13)	116(9)	159(15)	-31(8)	-10(10)	32(9)
C10C	135(13)	115(11)	153(13)	-22(9)	-20(9)	45(10)
C11C	123(11)	131(11)	149(13)	-6(10)	12(9)	54(10)
C12C	124(12)	109(9)	136(13)	0(7)	9(10)	49(8)
C13C	120(12)	94(9)	158(12)	5(8)	2(9)	23(8)
C14C	125(12)	98(9)	167(14)	-11(9)	2(10)	21(8)
C1	106(9)	108(7)	136(11)	-28(7)	28(8)	-9(6)
C2	114(9)	104(9)	145(10)	-14(7)	26(7)	-26(8)
C3	112(9)	119(9)	139(10)	-16(9)	6(7)	-45(8)
C4	95(10)	108(8)	128(11)	-8(6)	11(8)	-30(7)
C5	114(6)	109(5)	126(5)	-19(4)	5(4)	2(4)
C6	116(11)	93(7)	158(13)	-24(8)	5(9)	13(7)

O7	115(5)	111(5)	137(6)	-6(4)	-8(4)	2(4)
O8	110(4)	113(4)	162(8)	-54(5)	17(4)	-5(3)
C9	113(7)	129(6)	157(9)	-21(6)	5(7)	-15(6)
C10	124(6)	127(8)	159(8)	-33(5)	-6(6)	-5(6)
C11	114(6)	109(5)	126(5)	-19(4)	5(4)	2(4)
C12	94(5)	91(4)	114(6)	-6(4)	13(4)	1(4)
C13	142(8)	87(6)	130(6)	-9(4)	2(5)	-9(5)
C14	133(7)	104(5)	145(7)	-14(5)	2(6)	-26(5)

Supplementary Table 4 Bond Lengths for 9-X-ray.

Atom	Atom	Length/Å	Atom	Atom	Length/Å
O21A	C22A	1.393(5)	C27A	C26A	1.384(8)
O21A	C18A	1.397(5)	C5B	C6B	1.385(6)
O8B	C9B	1.357(10)	C5B	O8C	1.390(18)
O8B	C5B	1.422(11)	C24A	C25A	1.349(8)
O21B	C22B	1.379(6)	C3A	C4A	1.358(6)
O21B	C18B	1.398(5)	C25B	C26B	1.347(8)
C22A	C23A	1.355(6)	C25B	C24B	1.355(9)
C22A	C27A	1.376(6)	C23B	C24B	1.379(9)
C16B	C15B	1.365(5)	C26A	C25A	1.345(8)
C16B	C17B	1.389(6)	C4A	C5A	1.373(6)
C15B	C20B	1.377(6)	C5A	O7	1.387(14)
C15B	C1B	1.526(5)	C5A	O8	1.448(8)
C15A	C16A	1.369(6)	C14B	C9B	1.3900
C15A	C20A	1.373(6)	C14B	C11B	1.3900
C15A	C1A	1.527(6)	C9B	C10B	1.3900
C1A	C2A	1.530(6)	C10B	C12B	1.3900
C1A	C1A[1]	1.533(8)	C12B	C13B	1.3900
C22B	C27B	1.354(6)	C13B	C11B	1.3900
C22B	C23B	1.381(6)	O8C	C9C	1.39(2)
C1B	C2B	1.521(6)	C9C	C10C	1.3900
C1B	C1B[2]	1.540(8)	C9C	C14C	1.3900
C23A	C24A	1.368(6)	C10C	C11C	1.3900
C16A	C17A	1.378(6)	C11C	C12C	1.3900
C3B	C4B	1.382(6)	C12C	C13C	1.3900
C3B	C2B	1.387(5)	C13C	C14C	1.3900
C2B	C7B	1.372(6)	C1	O7	1.342(15)
C2A	C3A	1.375(6)	C1	C2	1.3900
C2A	C7A	1.382(6)	C1	C6	1.3900

C18B	C19B	1.348(6)	C2	C3	1.3900
C18B	C17B	1.370(6)	C3	C4	1.3900
C27B	C26B	1.359(6)	C4	C5	1.3900
C18A	C19A	1.350(6)	C5	C6	1.3900
C18A	C17A	1.372(6)	O8	C12	1.349(7)
C7A	C6A	1.385(6)	C9	C10	1.3900
C4B	C5B	1.356(6)	C9	C14	1.3900
C6A	C5A	1.354(6)	C10	C11	1.3900
C20A	C19A	1.376(6)	C11	C12	1.3900
C20B	C19B	1.375(6)	C12	C13	1.3900
C7B	C6B	1.363(6)	C13	C14	1.3900

[1]-X,1-Y,2-Z; [2]1-X,1-Y,1-Z

Supplementary Table 5 Bond Angles for 9-X-ray.

Atom	Atom	Atom	Angle/°	Atom	Atom	Atom	Angle/°
C22A	O21A	C18A	118.7(4)	C4B	C5B	O8B	127.3(6)
C9B	O8B	C5B	115.6(8)	C6B	C5B	O8B	112.8(6)
C22B	O21B	C18B	119.0(4)	C25A	C24A	C23A	121.2(6)
C23A	C22A	C27A	121.9(5)	C4A	C3A	C2A	122.6(4)
C23A	C22A	O21A	122.5(4)	C26B	C25B	C24B	119.9(7)
C27A	C22A	O21A	115.5(5)	C24B	C23B	C22B	119.8(6)
C15B	C16B	C17B	120.8(4)	C7B	C6B	C5B	119.2(5)
C16B	C15B	C20B	118.0(4)	C25B	C26B	C27B	121.6(6)
C16B	C15B	C1B	119.5(4)	C25A	C26A	C27A	119.6(6)
C20B	C15B	C1B	122.5(4)	C3A	C4A	C5A	119.2(5)
C16A	C15A	C20A	117.5(4)	C6A	C5A	C4A	120.3(5)
C16A	C15A	C1A	119.7(4)	C6A	C5A	O7	110.1(9)
C20A	C15A	C1A	122.8(4)	C4A	C5A	O7	122.3(7)
C15A	C1A	C2A	113.2(3)	C6A	C5A	O8	126.5(5)
C15A	C1A	C1A[1]	112.7(5)	C4A	C5A	O8	112.8(5)
C2A	C1A	C1A[1]	112.2(5)	C26A	C25A	C24A	121.0(6)
C27B	C22B	O21B	123.6(4)	C25B	C24B	C23B	119.5(6)
C27B	C22B	C23B	119.5(5)	C9B	C14B	C11B	120.0
O21B	C22B	C23B	116.8(5)	O8B	C9B	C10B	124.4(8)
C2B	C1B	C15B	112.6(3)	O8B	C9B	C14B	115.3(8)
C2B	C1B	C1B[2]	112.6(4)	C10B	C9B	C14B	120.0
C15B	C1B	C1B[2]	112.6(5)	C9B	C10B	C12B	120.0
C22A	C23A	C24A	118.0(5)	C13B	C12B	C10B	120.0
C15A	C16A	C17A	120.7(4)	C12B	C13B	C11B	120.0

C4B	C3B	C2B	121.1(4)	C13B	C11B	C14B	120.0
C7B	C2B	C3B	116.9(4)	C9C	O8C	C5B	129.4(18)
C7B	C2B	C1B	120.6(4)	O8C	C9C	C10C	92(2)
C3B	C2B	C1B	122.4(4)	O8C	C9C	C14C	147(2)
C3A	C2A	C7A	117.3(5)	C10C	C9C	C14C	120.0
C3A	C2A	C1A	120.6(4)	C11C	C10C	C9C	120.0
C7A	C2A	C1A	122.1(4)	C12C	C11C	C10C	120.0
C19B	C18B	C17B	121.1(4)	C11C	C12C	C13C	120.0
C19B	C18B	O21B	118.8(5)	C12C	C13C	C14C	120.0
C17B	C18B	O21B	120.0(5)	C13C	C14C	C9C	120.0
C22B	C27B	C26B	119.6(5)	O7	C1	C2	110.6(16)
C18B	C17B	C16B	119.1(4)	O7	C1	C6	129.1(16)
C19A	C18A	C17A	120.3(5)	C2	C1	C6	120.0
C19A	C18A	O21A	119.3(5)	C1	C2	C3	120.0
C17A	C18A	O21A	120.4(5)	C2	C3	C4	120.0
C2A	C7A	C6A	120.6(4)	C5	C4	C3	120.0
C5B	C4B	C3B	120.3(4)	C6	C5	C4	120.0
C5A	C6A	C7A	120.1(4)	C5	C6	C1	120.0
C15A	C20A	C19A	122.4(4)	C1	O7	C5A	120.5(12)
C18A	C19A	C20A	119.0(5)	C12	O8	C5A	118.8(5)
C19B	C20B	C15B	121.8(4)	C10	C9	C14	120.0
C18A	C17A	C16A	120.1(5)	C11	C10	C9	120.0
C6B	C7B	C2B	122.8(4)	C10	C11	C12	120.0
C18B	C19B	C20B	119.1(5)	O8	C12	C11	120.5(6)
C22A	C27A	C26A	118.4(6)	O8	C12	C13	119.5(6)
C4B	C5B	C6B	119.6(5)	C11	C12	C13	120.0
C4B	C5B	O8C	113.3(11)	C12	C13	C14	120.0
C6B	C5B	O8C	120.5(9)	C13	C14	C9	120.0

[1]-X,1-Y,2-Z; [2]1-X,1-Y,1-Z

Supplementary Table 6 Hydrogen Atom Coordinates (Å×10^4) and Isotropic Displacement Parameters (Å^2×10^3) for 9-X-ray.

**Atom**	***x***	***y***	***z***	**U(eq)**
H16B	4676.2	5240.69	8358.75	120
H1A	143.35	4598.56	8867.51	116
H1B	5162.29	5397.08	6148.93	112
H23A	-303.49	6478.35	4430.4	119
H16A	-340.36	4763.92	6600.85	123
H3B	5823.13	4021.32	4595.67	123
H27B	4672.62	3503.33	10588.14	125

H17B	3971.15	4701.29	9944.53	133
H7A	838.88	5975.83	10406.61	127
H4B	7005.71	3730.75	4667.58	139
H6A	2029.99	6253.28	10372.68	136
H20A	-452.75	6114.89	9409.21	143
H19A	-1138.76	6665.15	7791.84	147
H20B	4561.41	3878.04	5567.49	141
H17A	-1044.36	5306.05	4987.27	132
H7B	6312.51	5445.53	7042.86	134
H19B	3853.66	3344.63	7122.95	143
H27A	-2343.39	6704.75	3162.58	153
H24A	48.33	6886.37	2259.64	147
H3A	1288.07	4538.26	8006.67	138
H25B	4166.23	2804.39	14386.74	181
H23B	2628.14	3363.3	11658.13	164
H6B	7477.45	5144.61	7188.66	154
H26B	4998.24	3093.39	12773.31	160
H26A	-1960.72	7117.75	990.9	177
H4A	2454.06	4828.13	7907.99	160
H25A	-771.49	7192.82	573.52	169
H24B	2975.37	2942.21	13859.6	189
H14B	7908.43	3174.77	7060.87	128
H10B	8829.51	4128.82	3896.34	124
H12B	9316.41	3246.6	2980.62	143
H13B	9099.33	2328.46	4105.02	149
H11B	8395.34	2292.53	6145.14	143
H10C	7831.05	3628.26	6696.74	161
H11C	8227.07	2637.82	6915.09	161
H12C	9024.38	2250.4	5259.76	148
H13C	9425.67	2853.42	3386.06	149
H14C	9029.65	3843.86	3167.68	156
H2	2738.51	6452.79	8127.62	144
H3	3210.08	7420.12	7969.73	148
H4	4075.59	7739.63	9638.72	132
H5	4469.54	7091.81	11465.63	139
H6	3997.98	6124.47	11623.54	147
H9	4123.77	7694.52	10759.55	159
H10	4364.1	6788.97	11941.79	164
H11	3876.24	5894.54	11055.99	139
H13	2907.7	6811.2	7805.72	143

H14	3395.56	7705.63	8691.49	153

Supplementary Table 7 Atomic Occupancy for 9-X-ray .

**Atom**	***Occupancy***	**Atom**	***Occupancy***	**Atom**	***Occupancy***
O8B	0.646(16)	C14B	0.646(16)	H14B	0.646(16)
C9B	0.646(16)	C10B	0.646(16)	H10B	0.646(16)
C12B	0.646(16)	H12B	0.646(16)	C13B	0.646(16)
H13B	0.646(16)	C11B	0.646(16)	H11B	0.646(16)
O8C	0.354(16)	C9C	0.354(16)	C10C	0.354(16)
H10C	0.354(16)	C11C	0.354(16)	H11C	0.354(16)
C12C	0.354(16)	H12C	0.354(16)	C13C	0.354(16)
H13C	0.354(16)	C14C	0.354(16)	H14C	0.354(16)
C1	0.355(17)	C2	0.355(17)	H2	0.355(17)
C3	0.355(17)	H3	0.355(17)	C4	0.355(17)
H4	0.355(17)	C5	0.355(17)	H5	0.355(17)
C6	0.355(17)	H6	0.355(17)	O7	0.355(17)
O8	0.645(17)	C9	0.645(17)	H9	0.645(17)
C10	0.645(17)	H10	0.645(17)	C11	0.645(17)
H11	0.645(17)	C12	0.645(17)	C13	0.645(17)
H13	0.645(17)	C14	0.645(17)	H14	0.645(17)

ii. Deuterium experiments

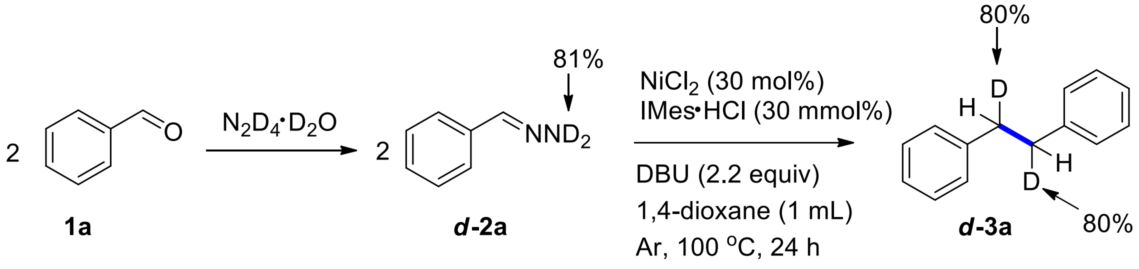

The deuterated hydrazone **d-2a** was synthesized according to the literature[24]. Hydrazone ***d*-2a** (81% D). ^{1}H NMR (400 MHz, $CDCl_3$) δ: 7.76 (s, 1H), 7.55 (d, *J* = 7.0 Hz, 2H), 7.33-7.26 (m, 3H), 5.53 (br, 0.38H). The other operations were following the general procedure. Product ***d*-3a**, 72% yield; ^{1}H NMR (400 MHz, $CDCl_3$) δ: 7.35 – 7.29 (m, 4H), 7.23 (t, *J* = 6.8 Hz, 6H), 2.96 (s, 2.39H). Deuterium incorporation was determined by ^{1}H NMR. (Note: the two benzylic hydrogen atoms were chemically equivalent. Percent deuterium (% D) incorporation is depicted as the amount of deuterium in place of the combined hydrogen atoms at that site).

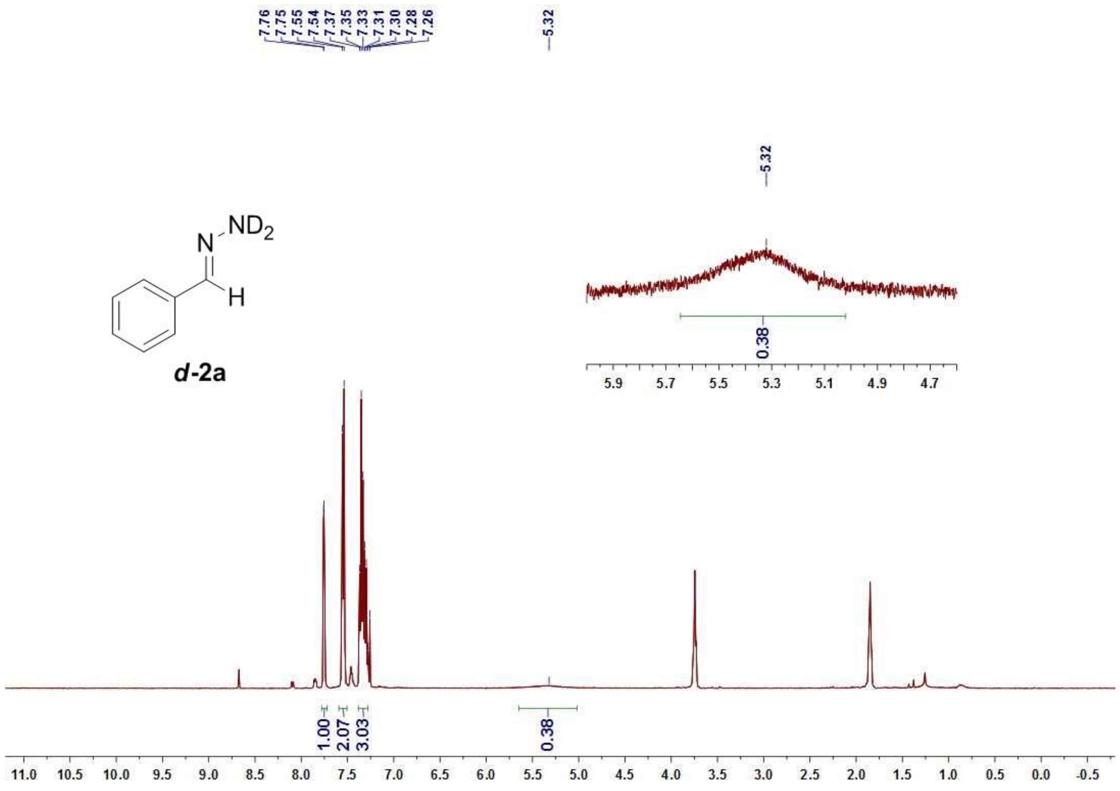

Supplementary Figure 2 ^{1}H NMR of **d-3a**

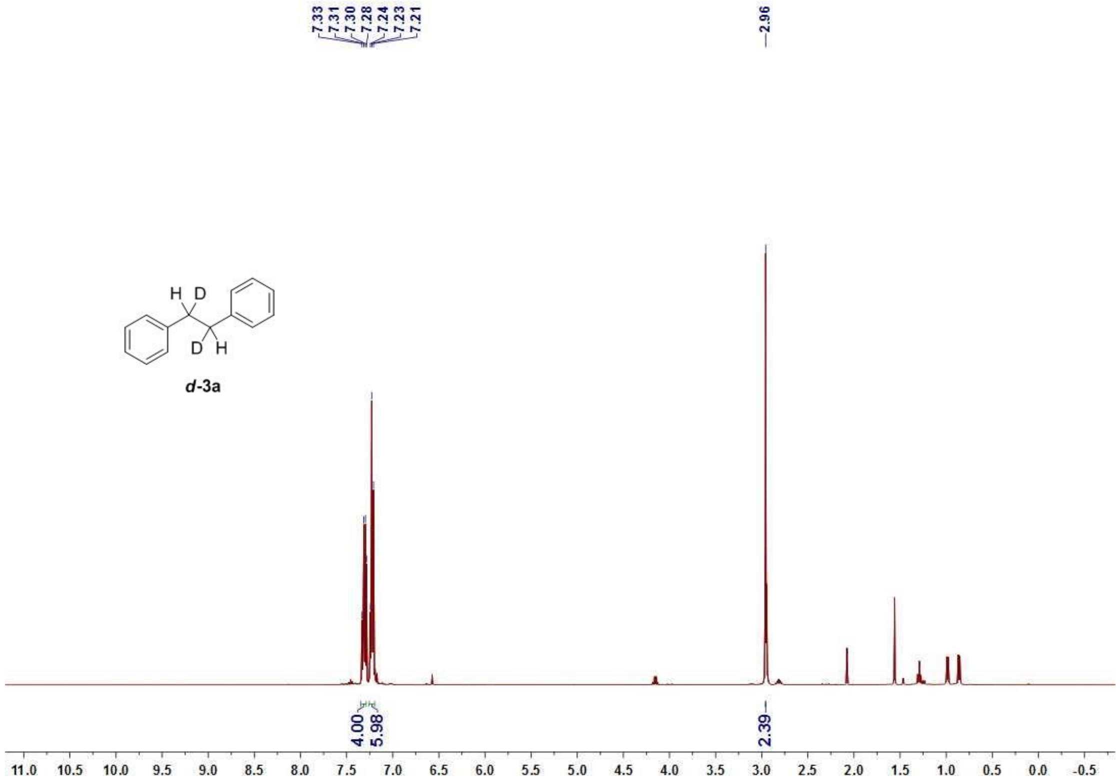

Supplementary Figure 3 ^{13}C NMR of **d-3a**

Ⅳ. Supplementary Figures

NMR spectra of products

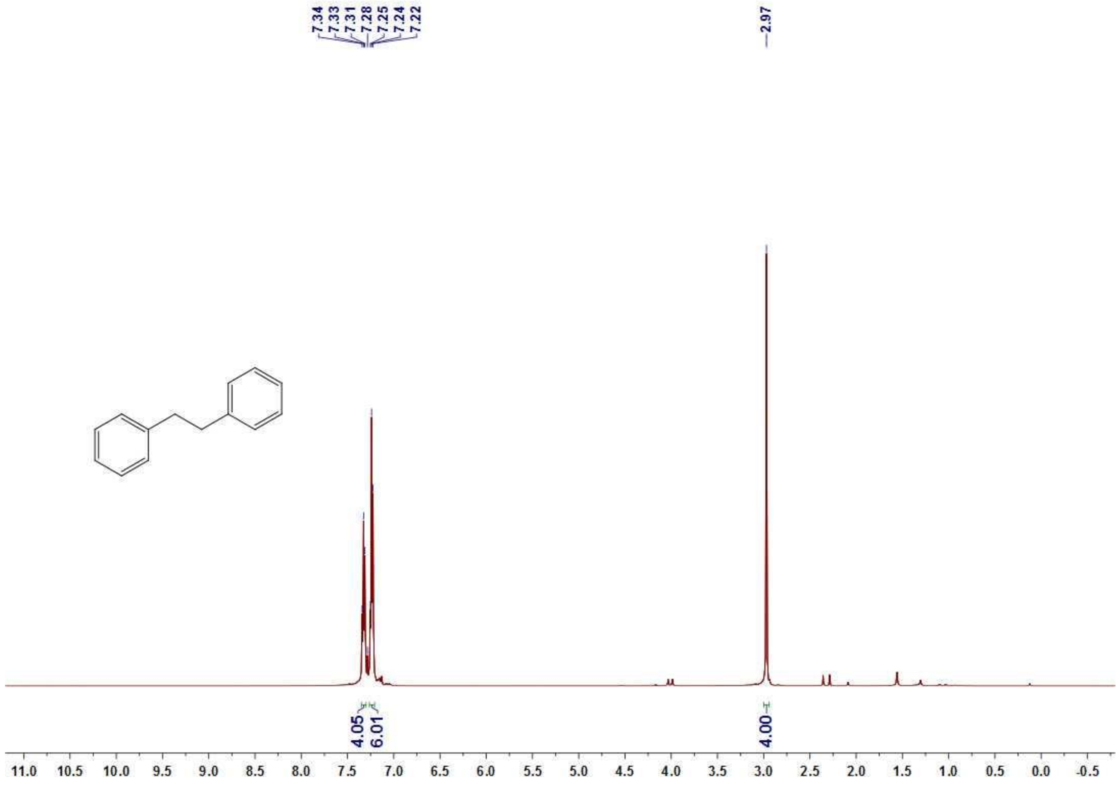

Supplementary Figure 4 ^{1}H NMR of **1,2-Diphenylethane**

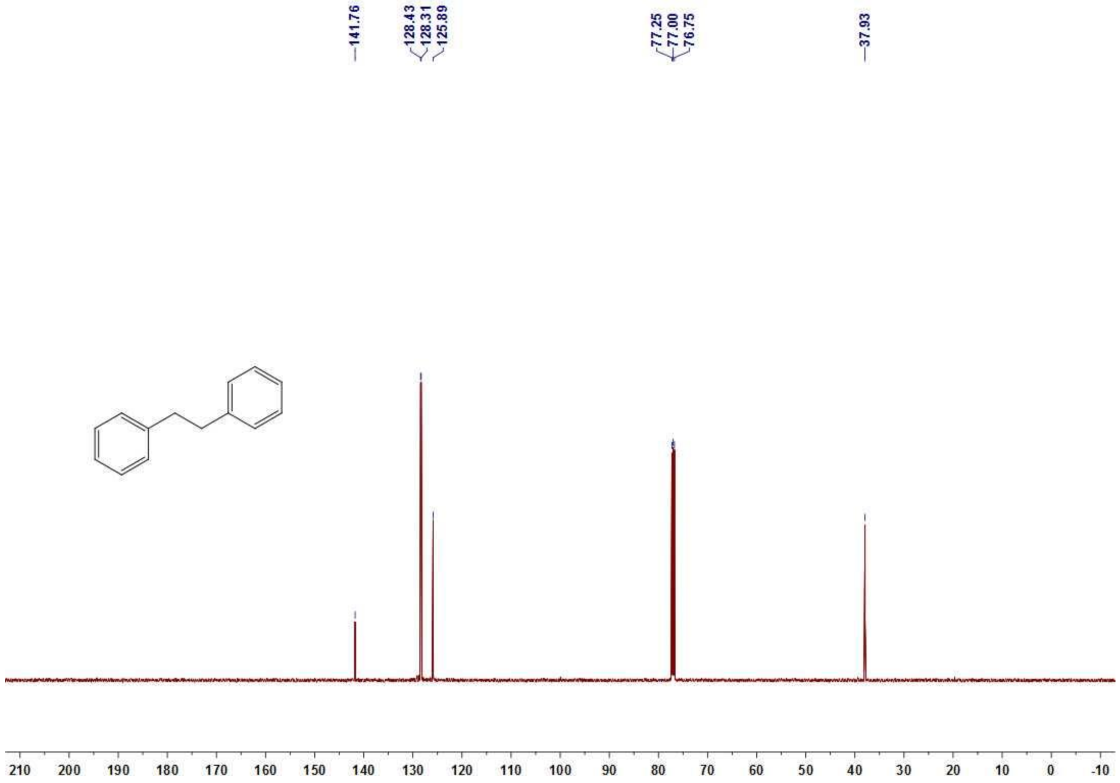

Supplementary Figure 5 ^{13}C NMR of **1,2-Diphenylethane**

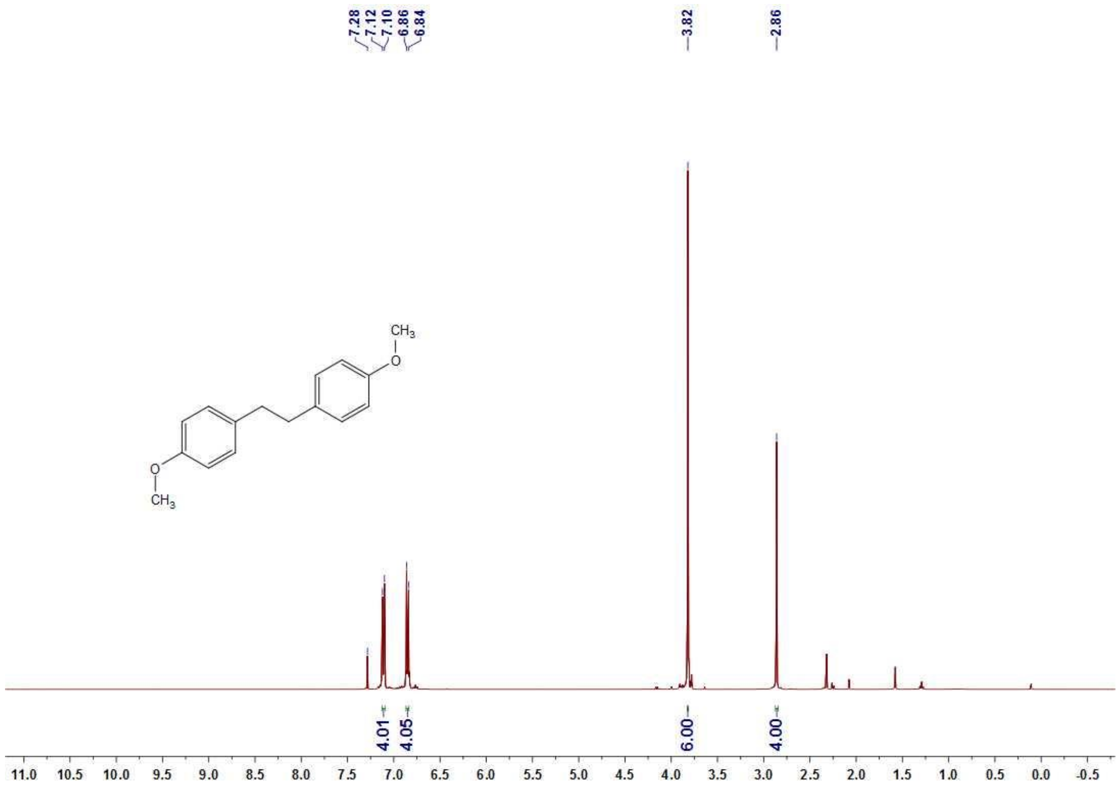

Supplementary Figure 6 ^{1}H NMR of **1,2-Bis(4-methoxyphenyl)ethane**

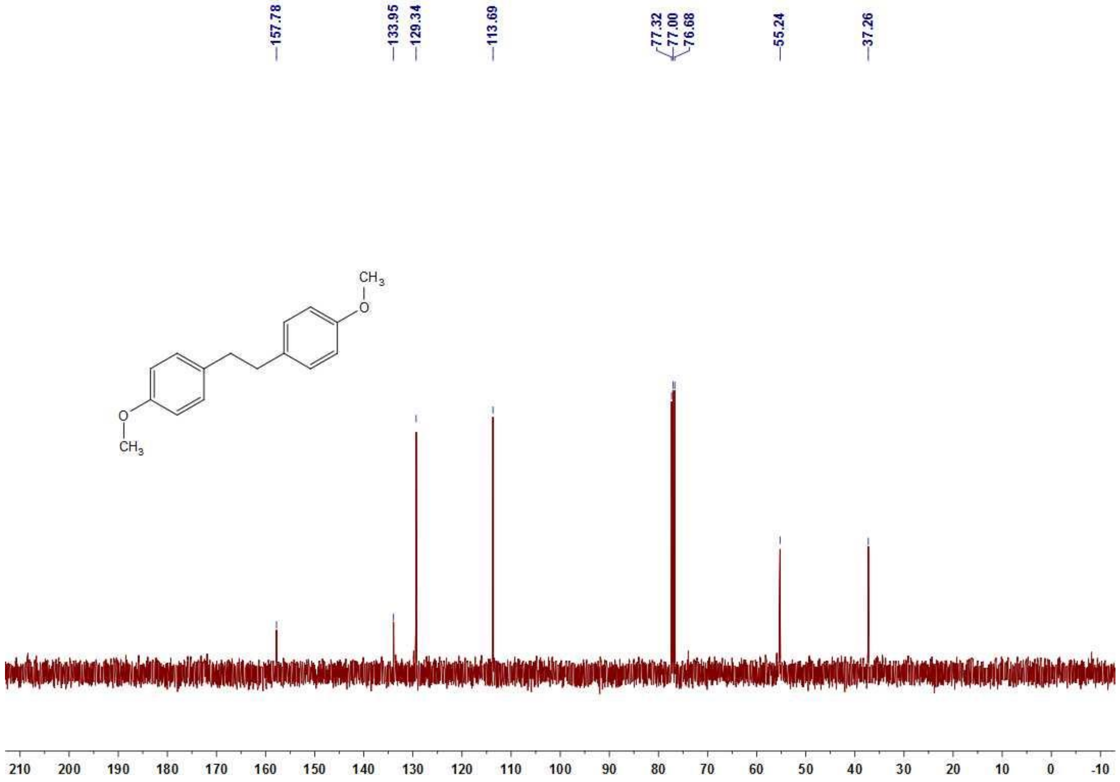

Supplementary Figure 7 ^{13}C NMR of **1,2-Bis(4-methoxyphenyl)ethane**

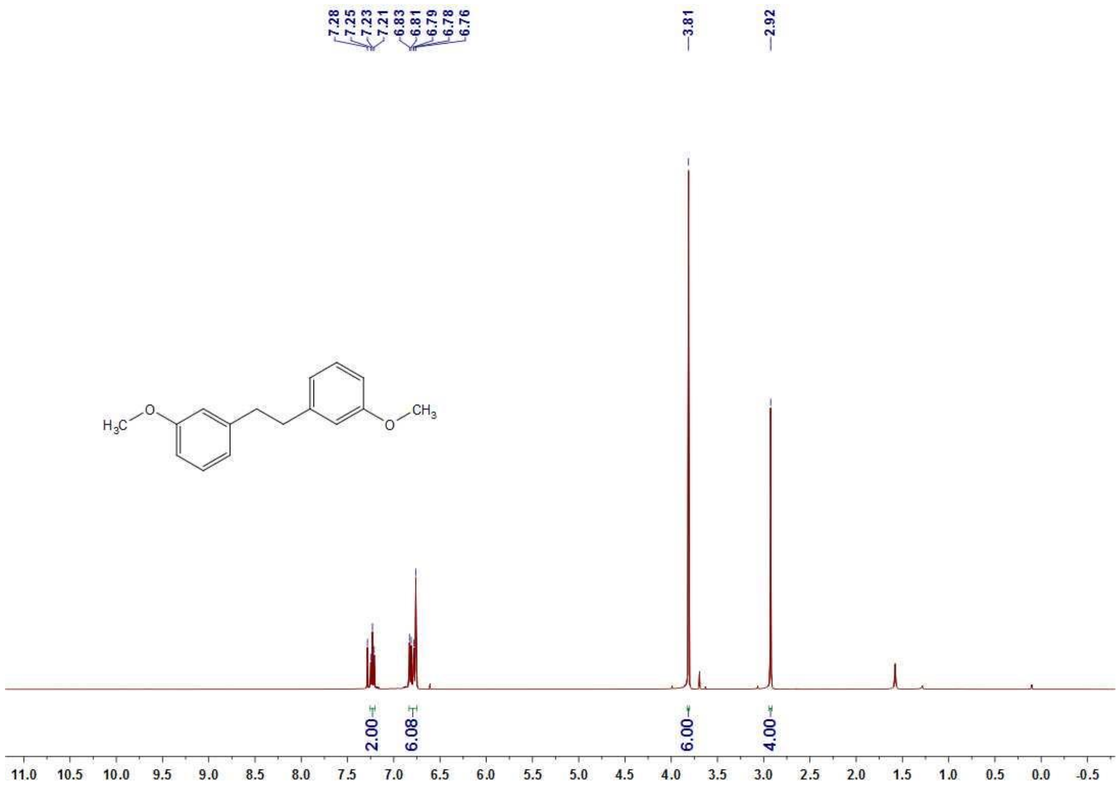

Supplementary Figure 8 ^{1}H NMR of **1,2-Bis(3-methoxyphenyl)ethane**

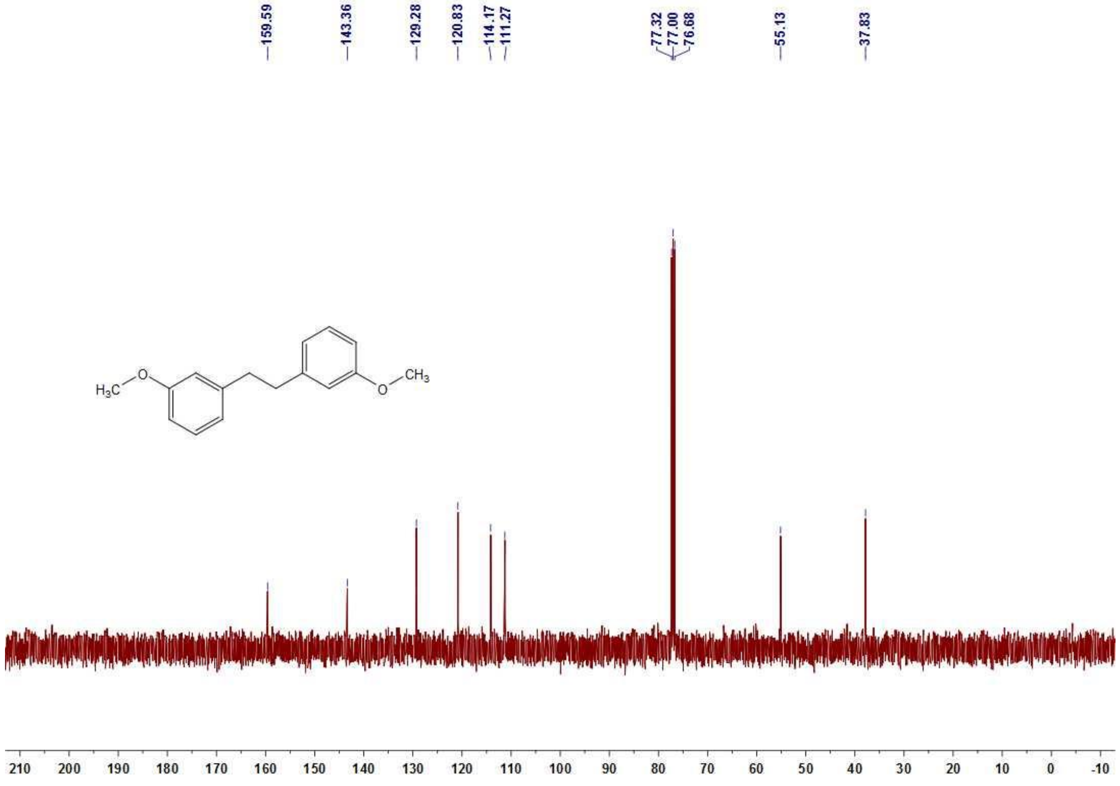

Supplementary Figure 9 ^{13}C NMR of **1,2-Bis(3-methoxyphenyl)ethane**

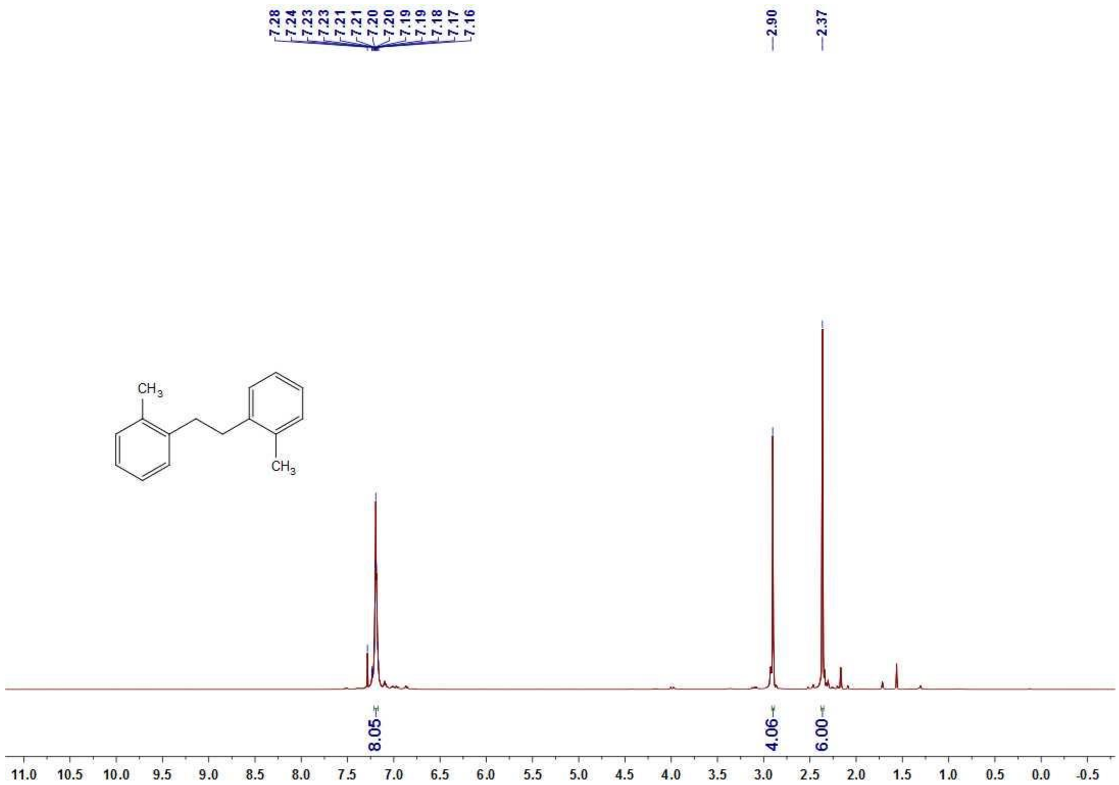

Supplementary Figure 10 ^{1}H NMR of **1,2-Di-o-tolylethane**

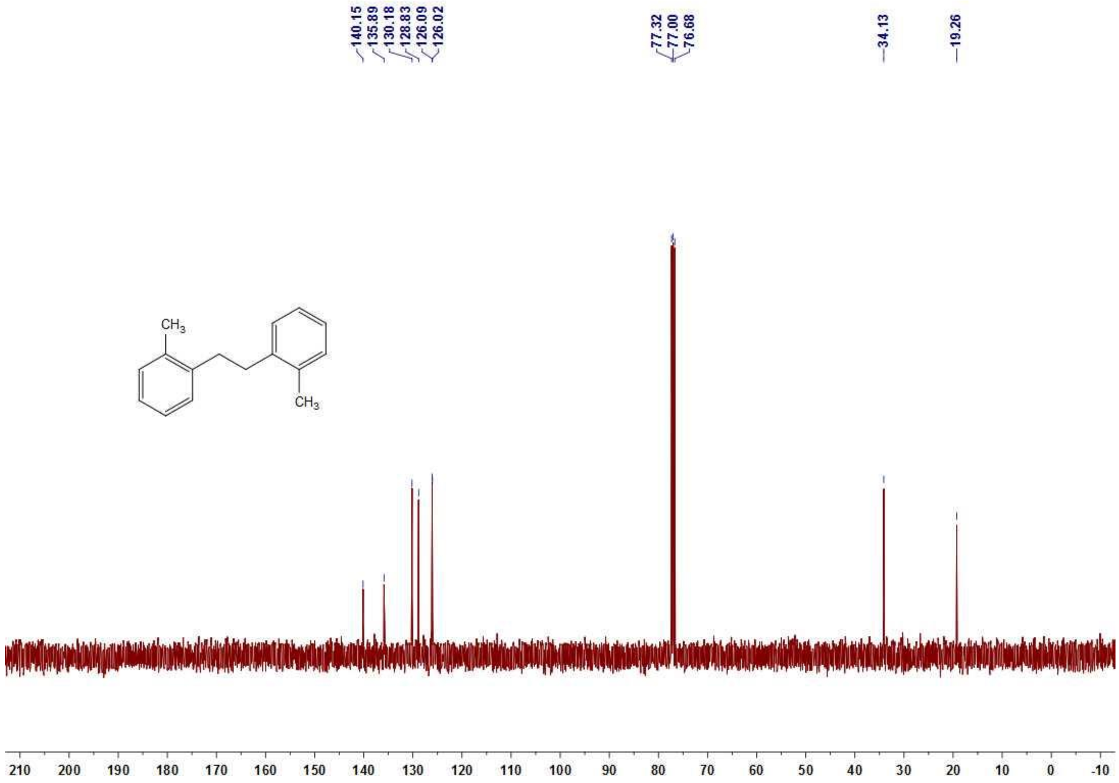

Supplementary Figure 11 ^{13}C NMR of **1,2-Di-o-tolylethane**

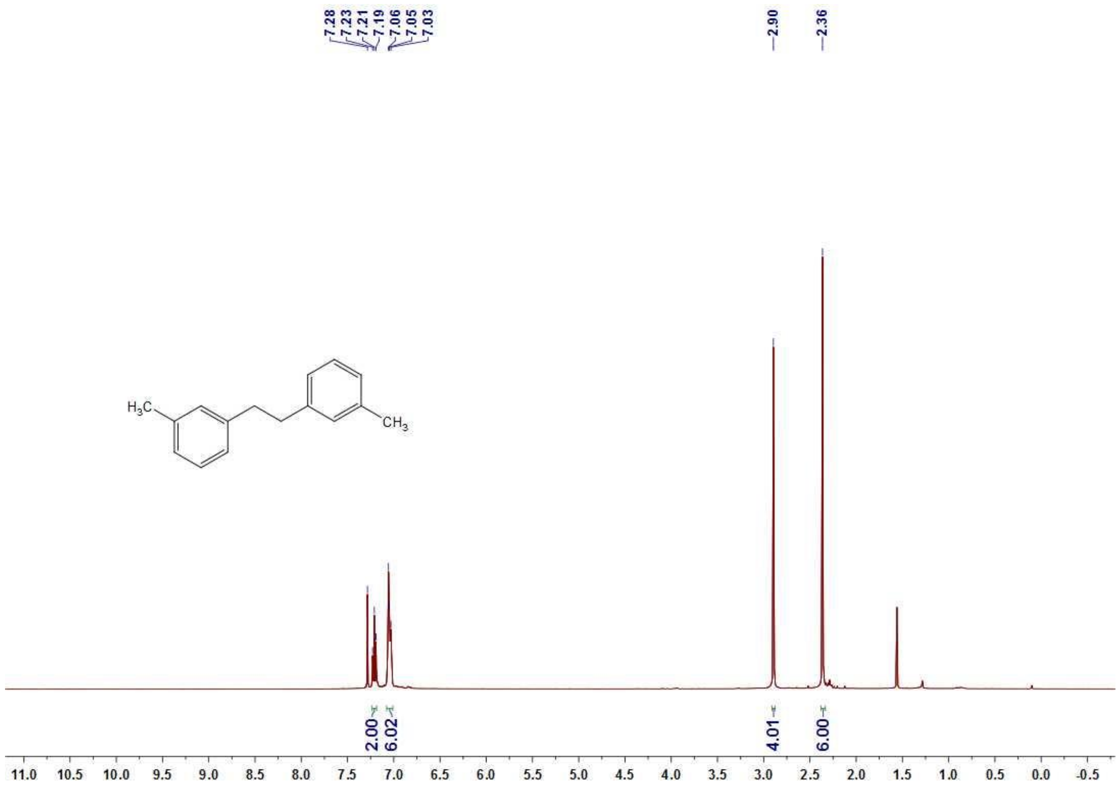

Supplementary Figure 12 ^{1}H NMR of **1,2-Di-m-tolylethane**

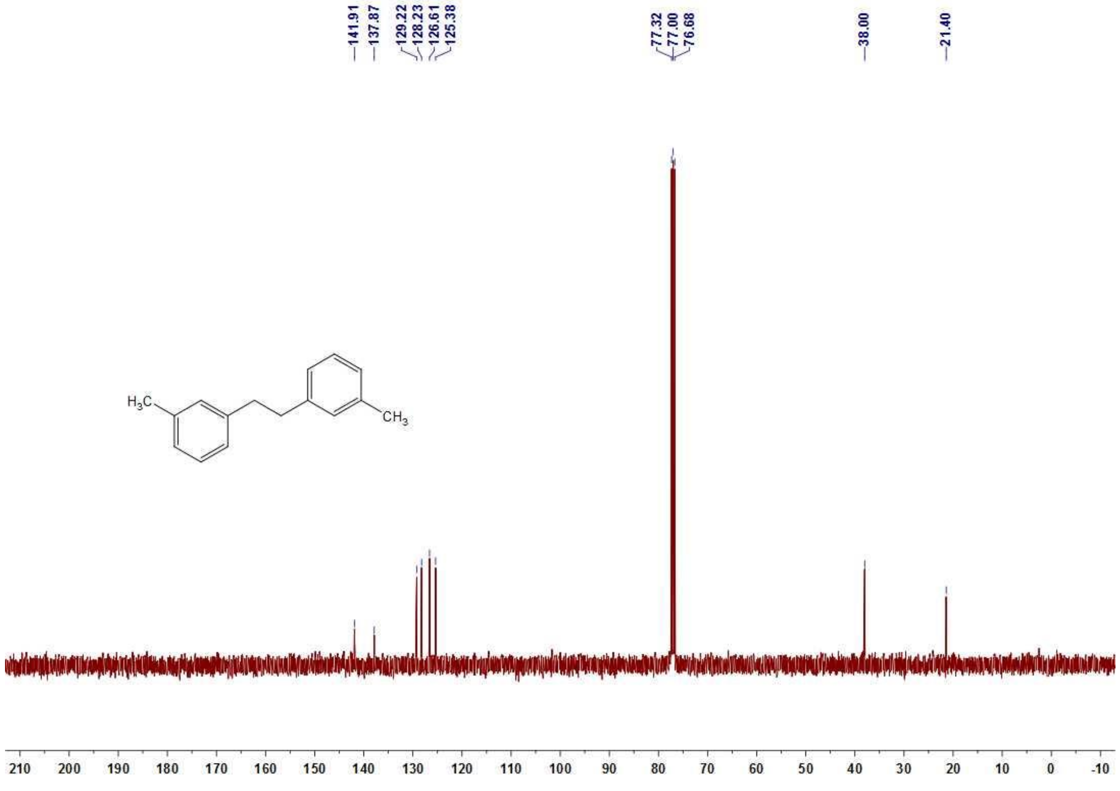

Supplementary Figure 13 ^{13}C NMR of **1,2-Di-m-tolylethane**

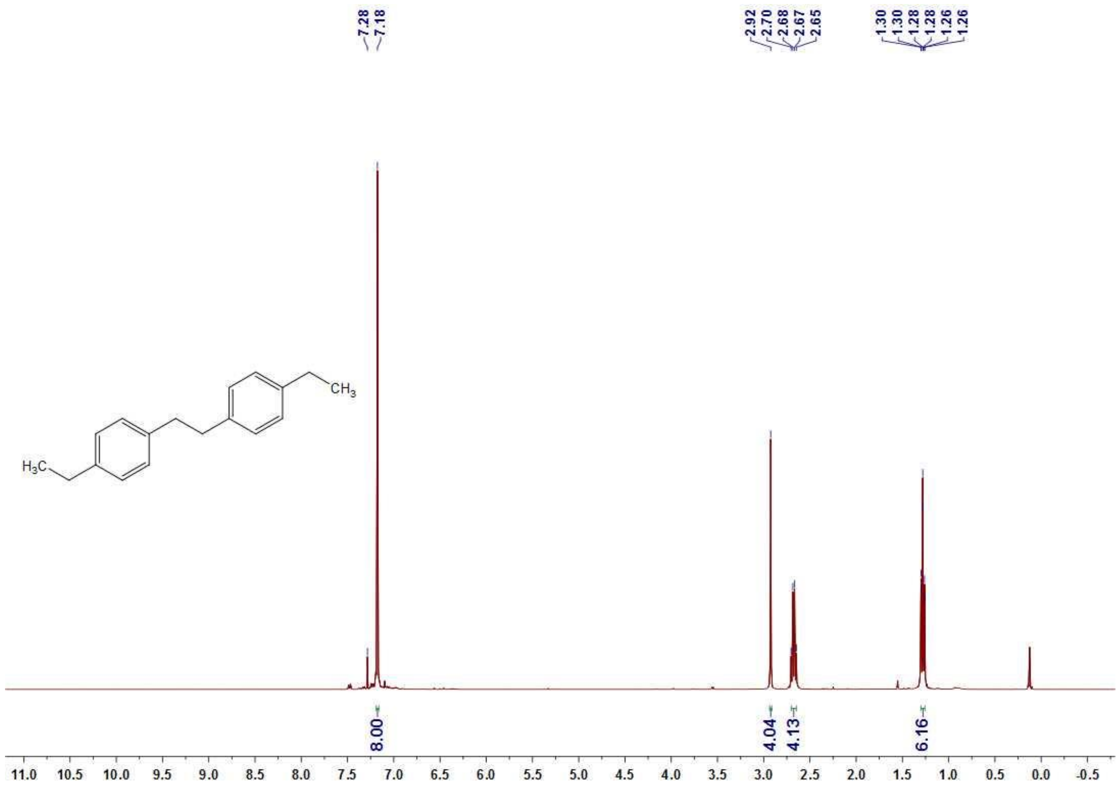

Supplementary Figure 14 ^{1}H NMR of **1,2-Bis(4-ethylphenyl)ethane**

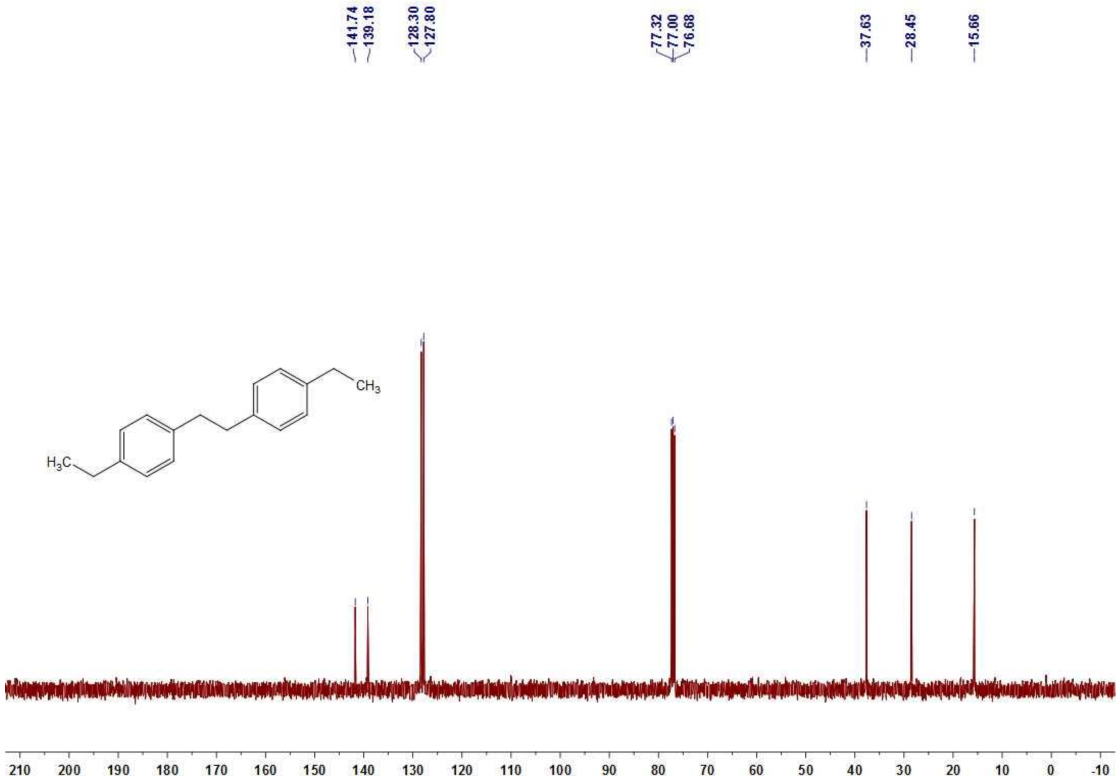

Supplementary Figure 15 ^{13}C NMR of **1,2-Bis(4-ethylphenyl)ethane**

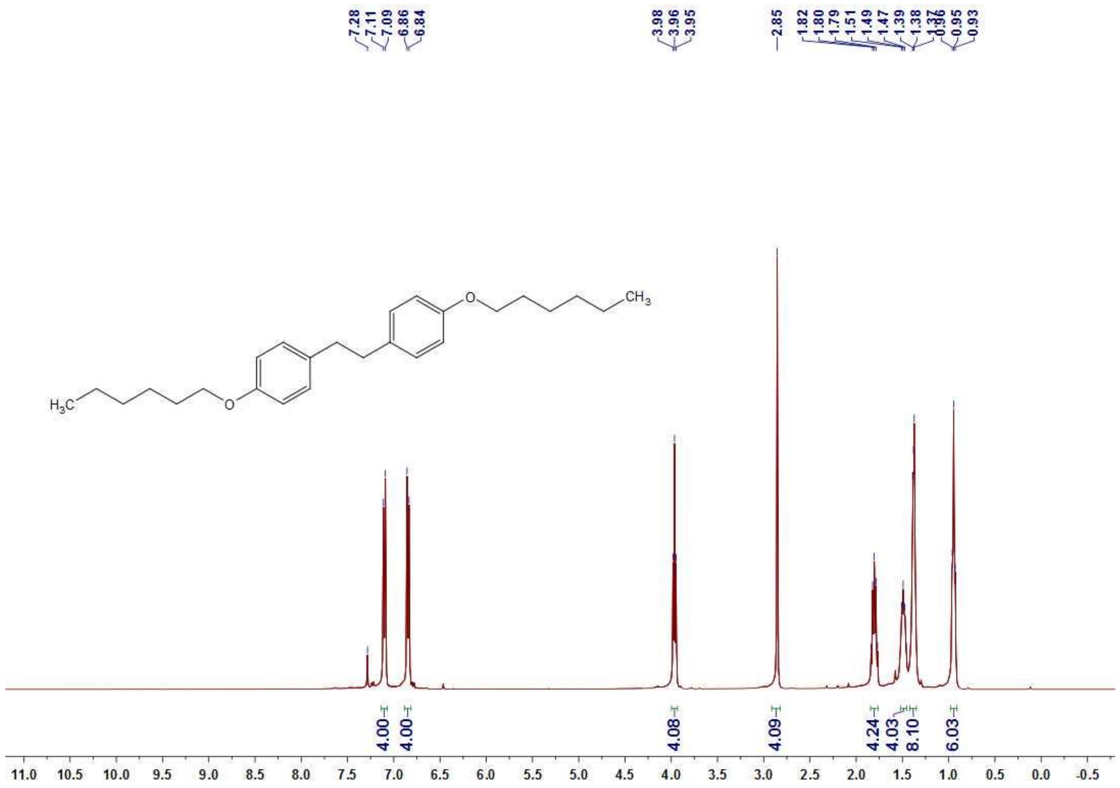

Supplementary Figure 16 ^{1}H NMR of **1,2-Bis(4-(hexyloxy)phenyl)ethane**

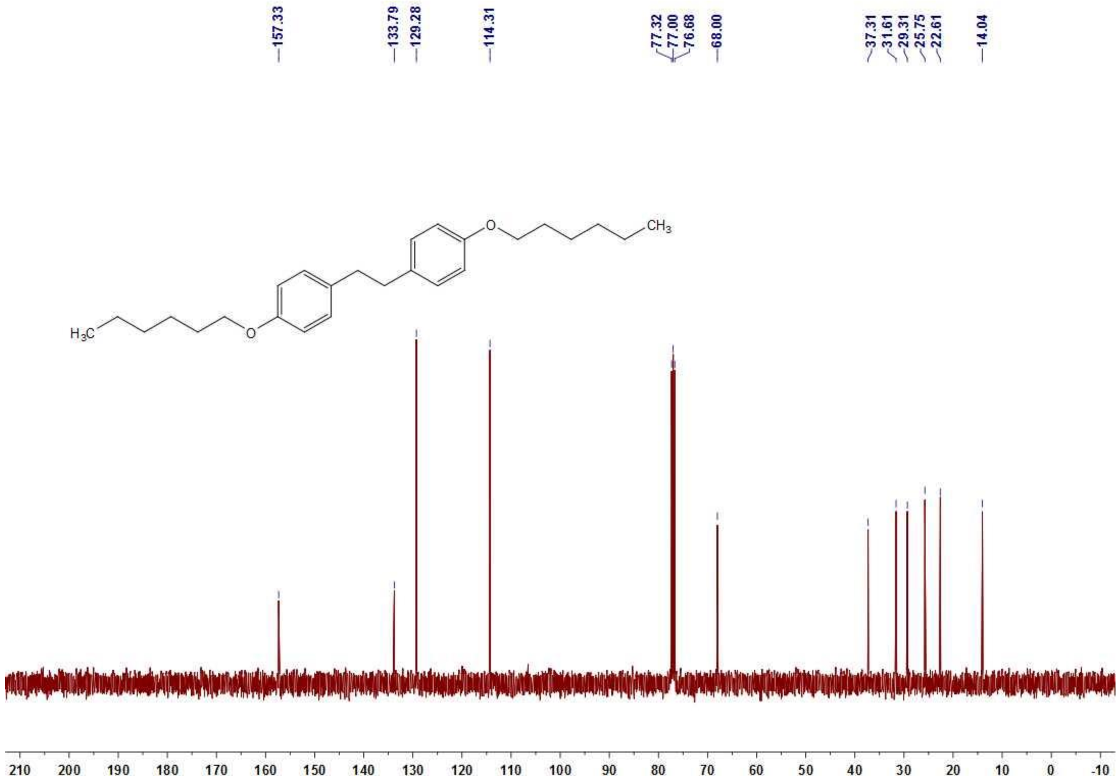

Supplementary Figure 17 ^{13}C NMR of **1,2-Bis(4-(hexyloxy)phenyl)ethane**

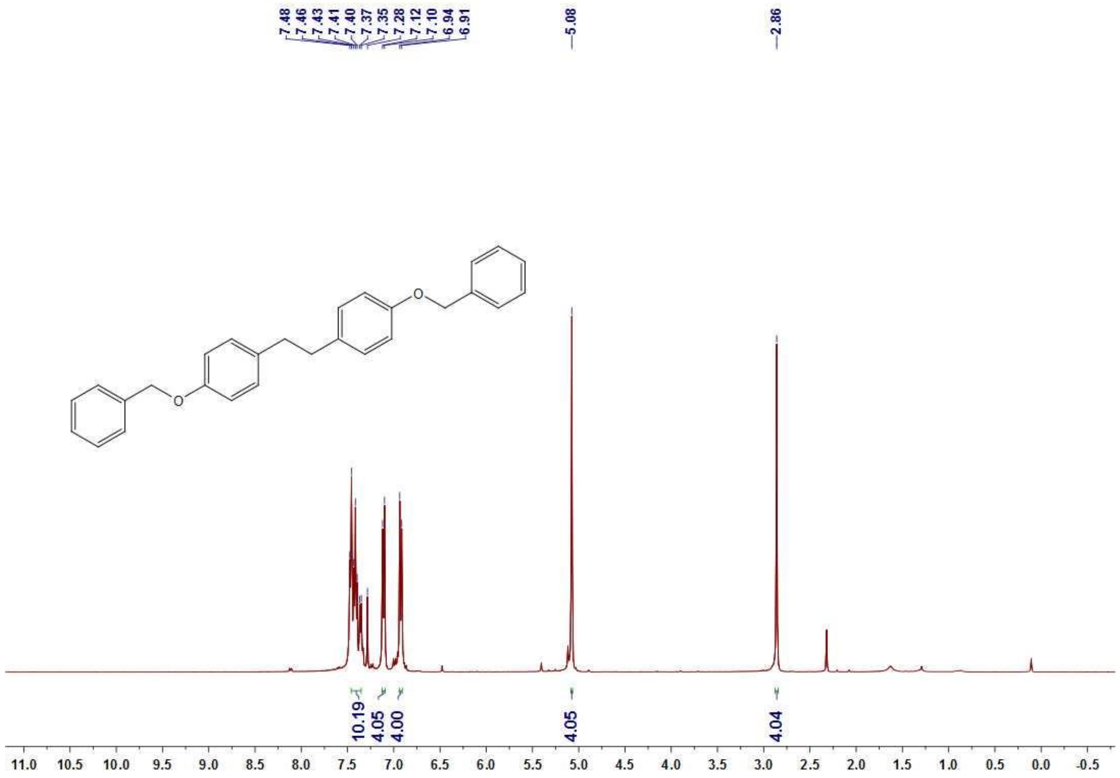

Supplementary Figure 18 ^{1}H NMR of **1,2-Bis(4-(benzyloxy)phenyl)ethane**

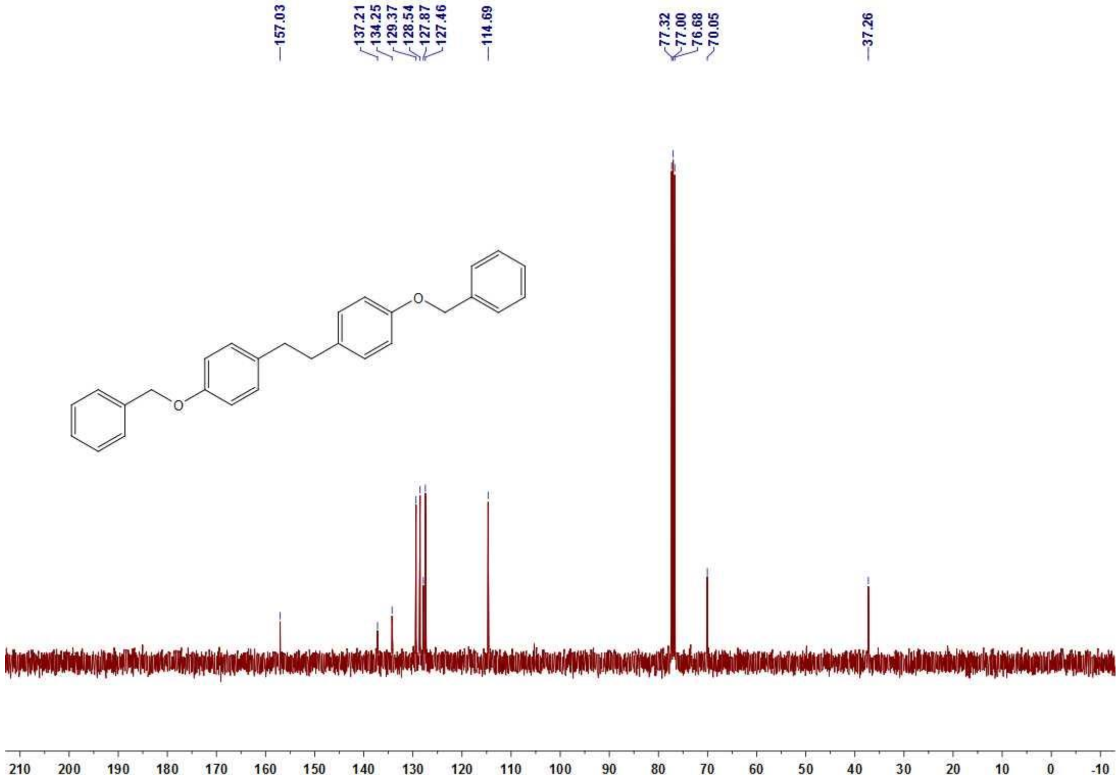

Supplementary Figure 19 ^{13}C NMR of **1,2-Bis(4-(benzyloxy)phenyl)ethane**

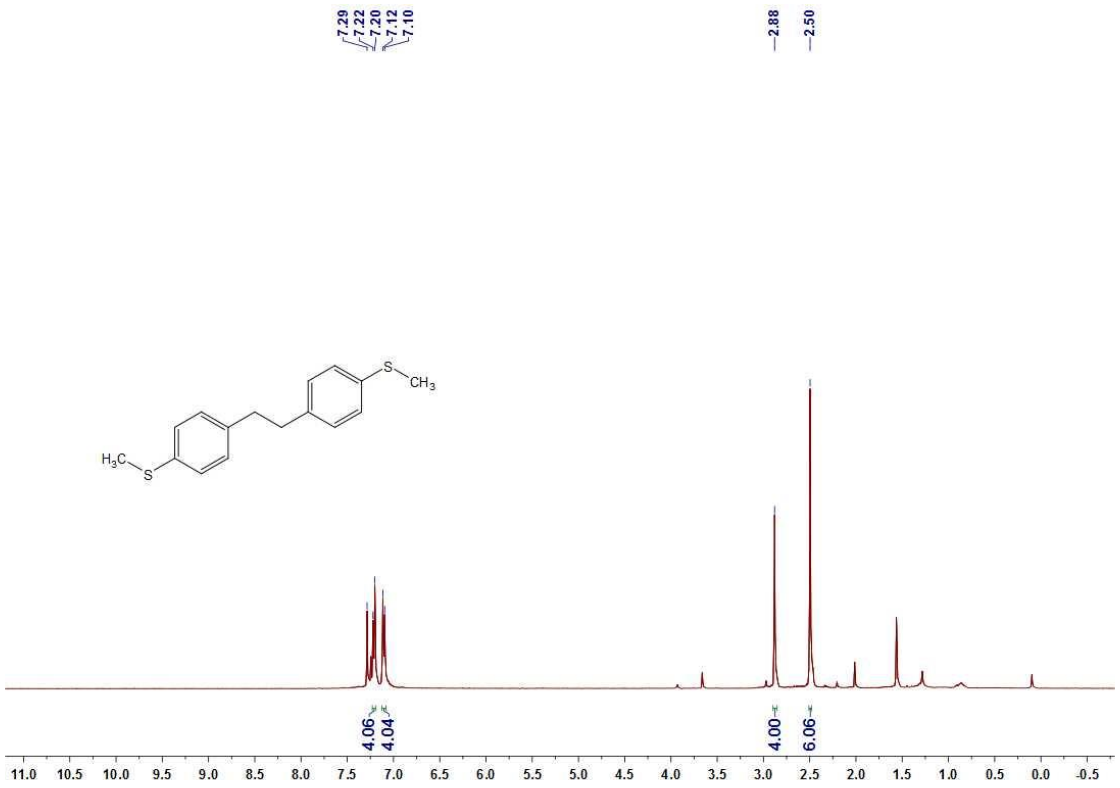

Supplementary Figure 20 ^{1}H NMR of **1,2-Bis(4-(methylthio)phenyl)ethane**

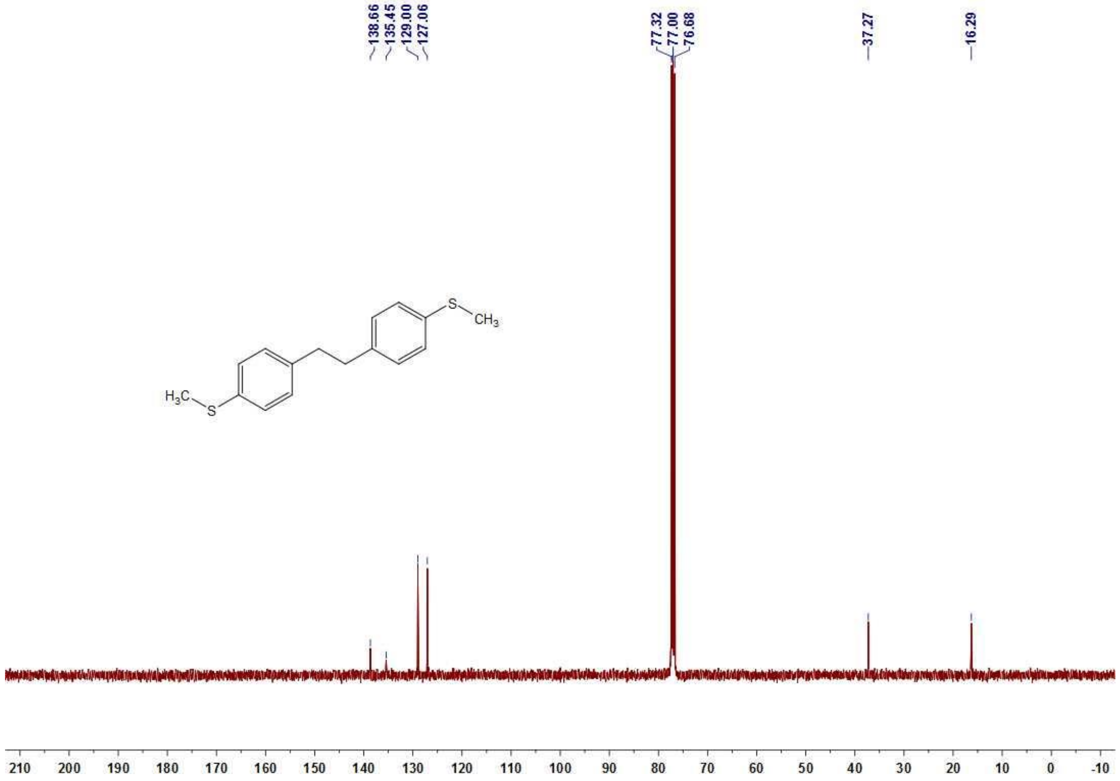

Supplementary Figure 21 ^{13}C NMR of **1,2-Bis(4-(methylthio)phenyl)ethane**

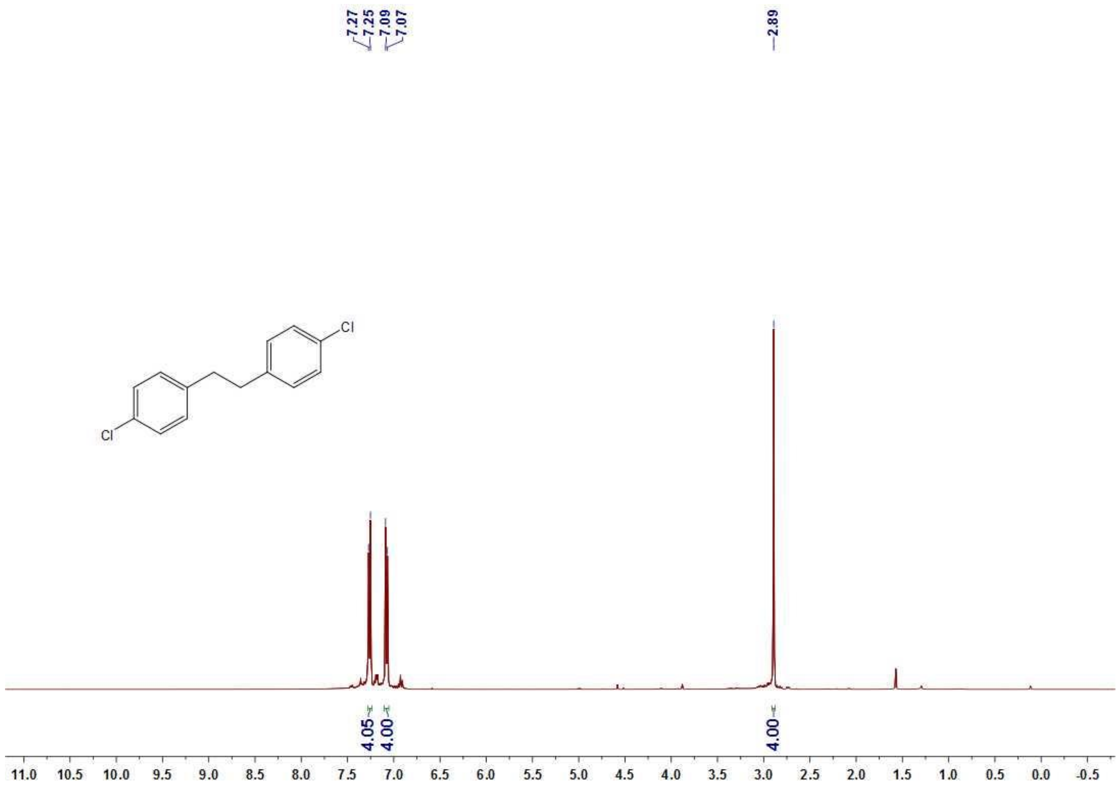

Supplementary Figure 22 ^{1}H NMR of **1,2-Bis(4-chlorophenyl)ethane**

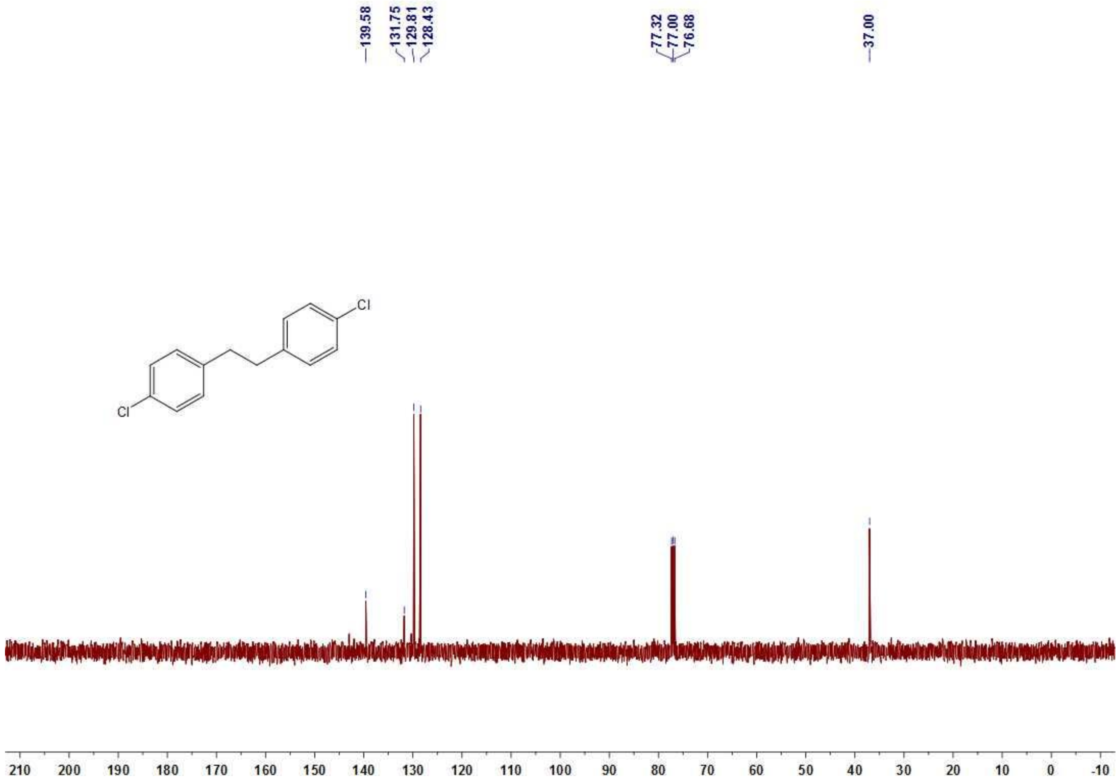

Supplementary Figure 23 ^{13}C NMR of **1,2-Bis(4-chlorophenyl)ethane**

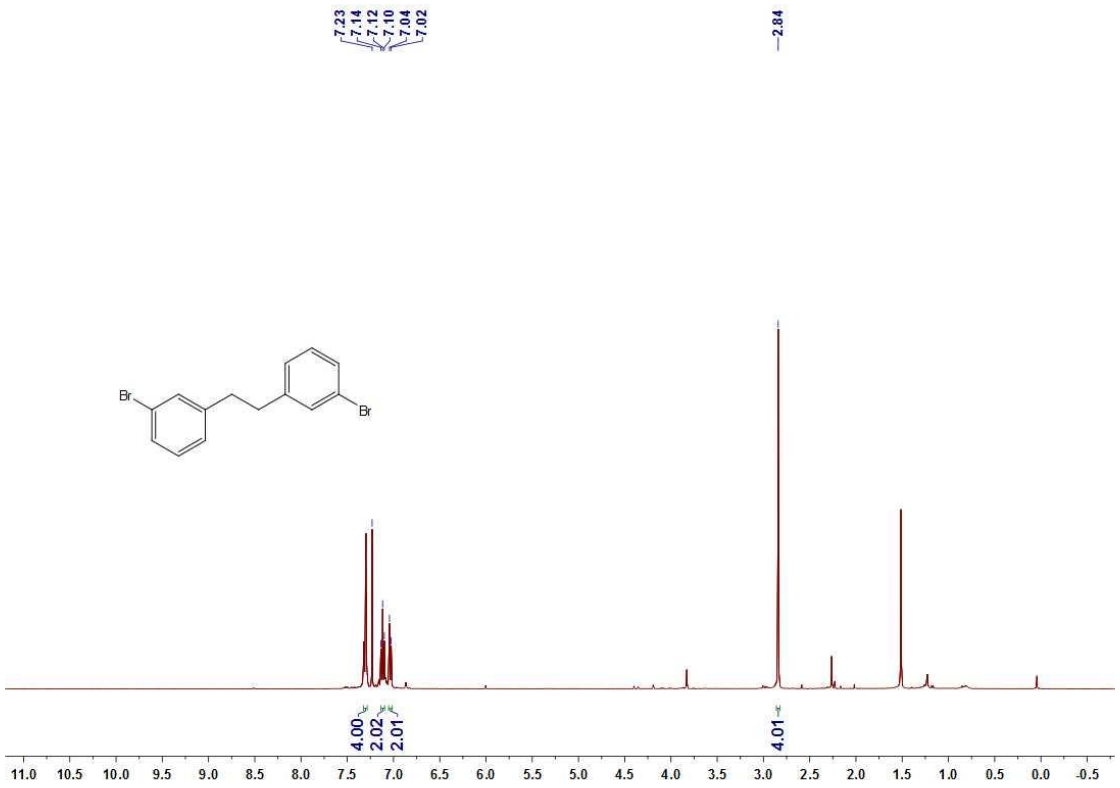

Supplementary Figure 24 ^{1}H NMR of **1,2-Bis(3-bromophenyl)ethane**

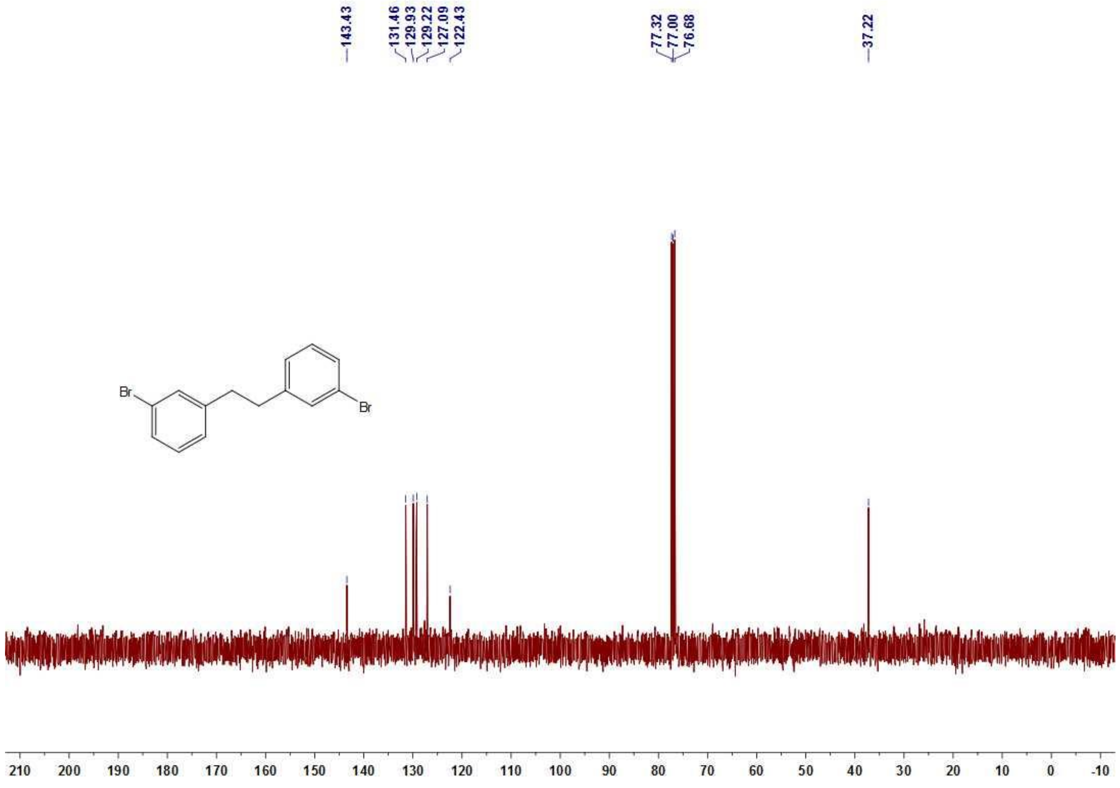

Supplementary Figure 25 ^{13}C NMR of **1,2-Bis(3-bromophenyl)ethane**

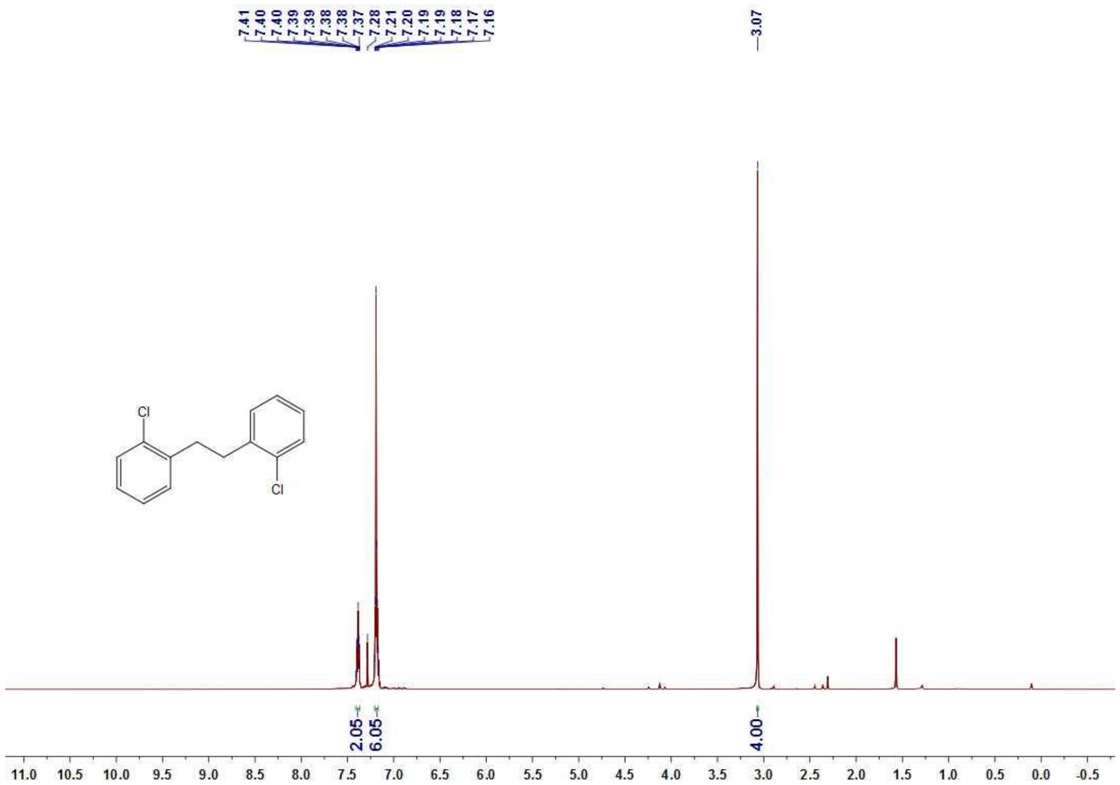

Supplementary Figure 26 ^{1}H NMR of **1,2-Bis(2-chlorophenyl)ethane**

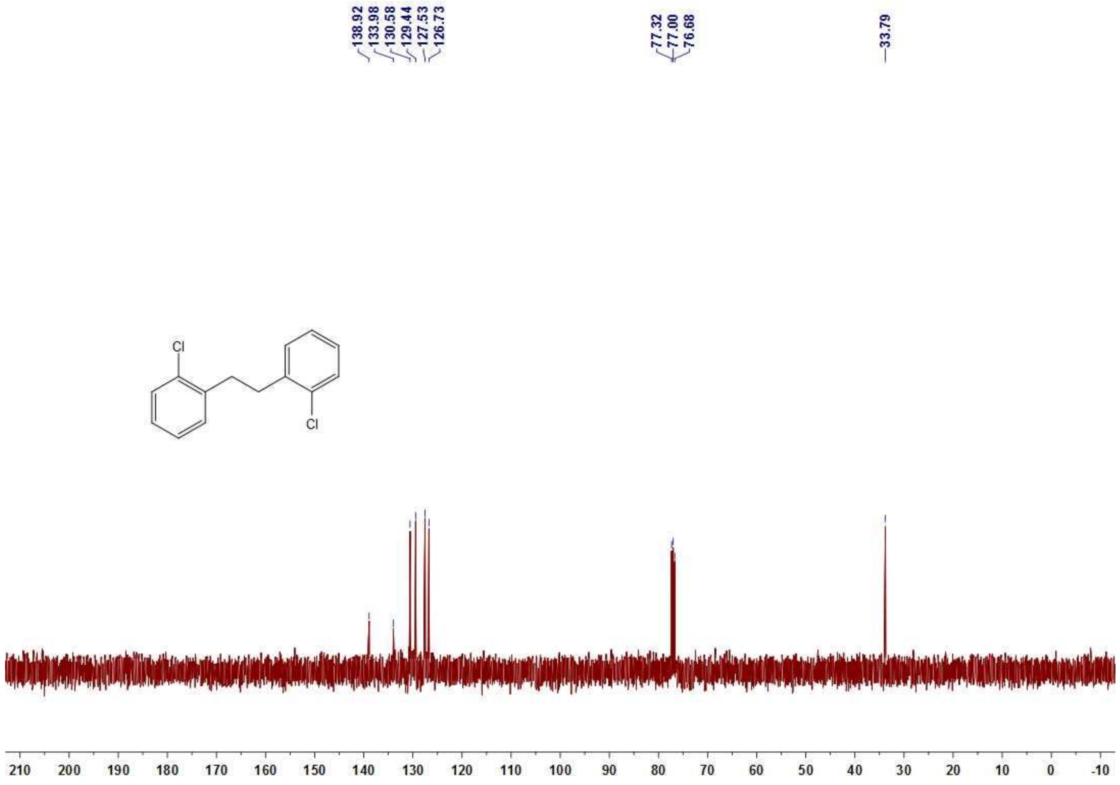

Supplementary Figure 27 ^{13}C NMR of **1,2-Bis(2-chlorophenyl)ethane**

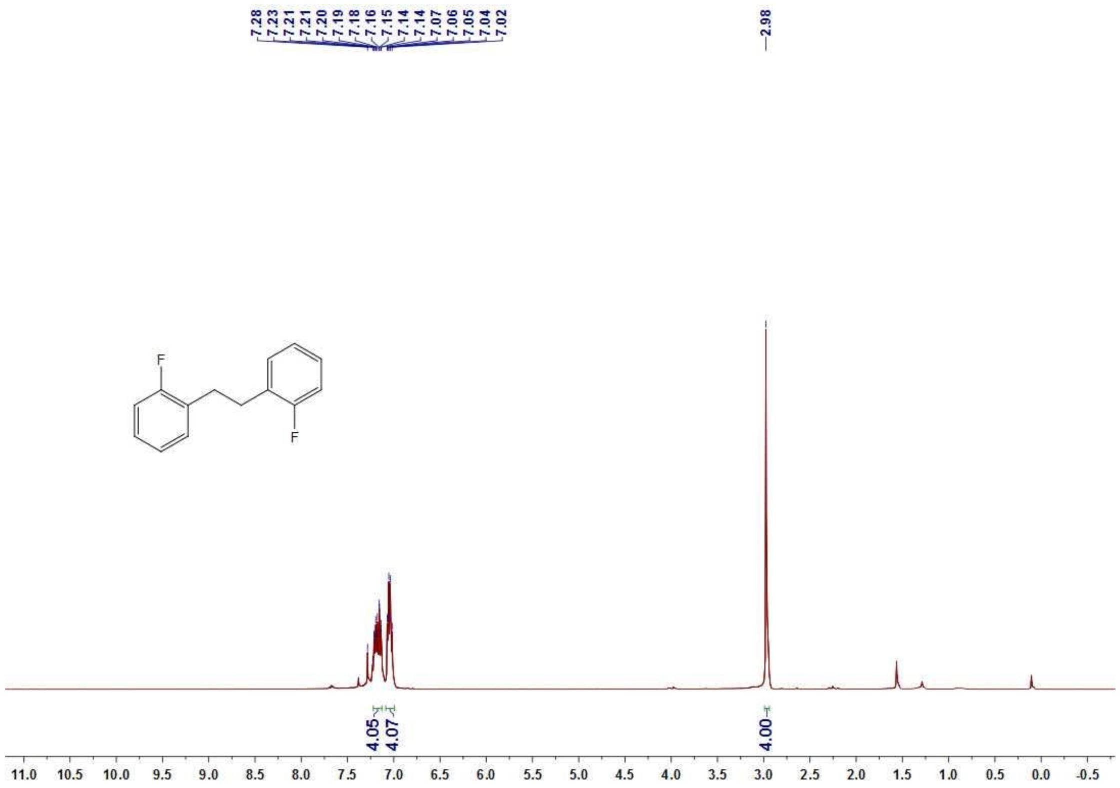

Supplementary Figure 28 ^{1}H NMR of **1,2-Bis(2-fluorophenyl)ethane**

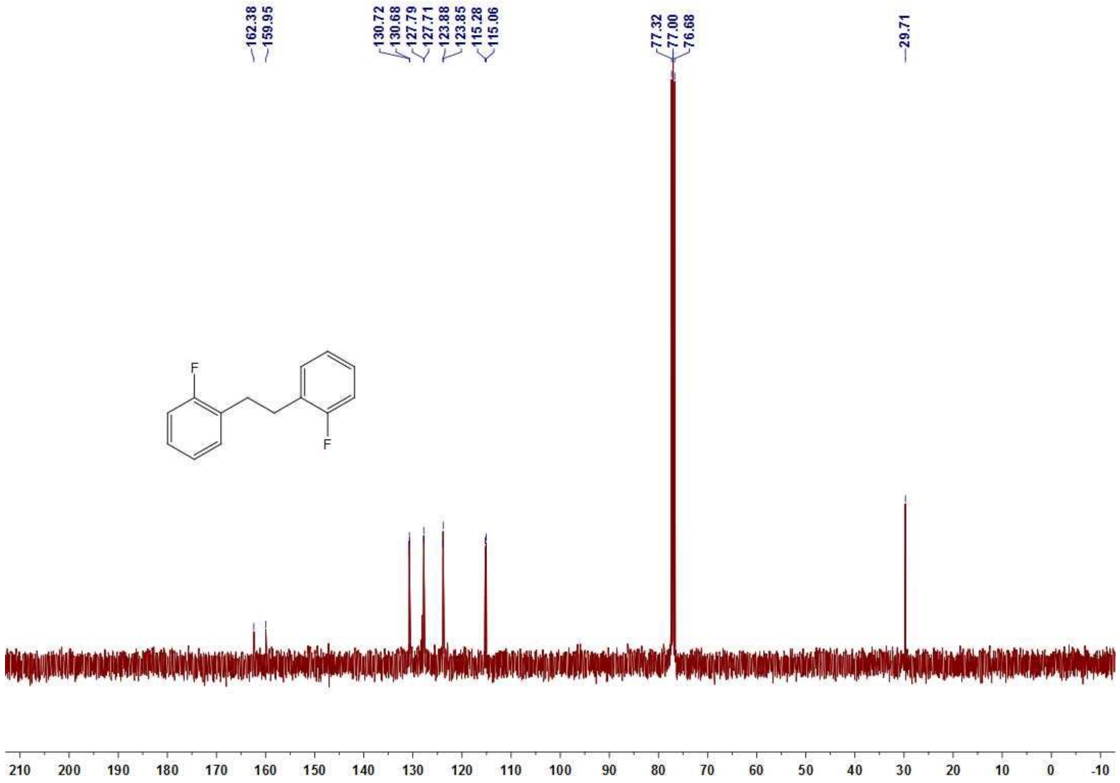

Supplementary Figure 29 ^{13}C NMR of **1,2-Bis(2-fluorophenyl)ethane**

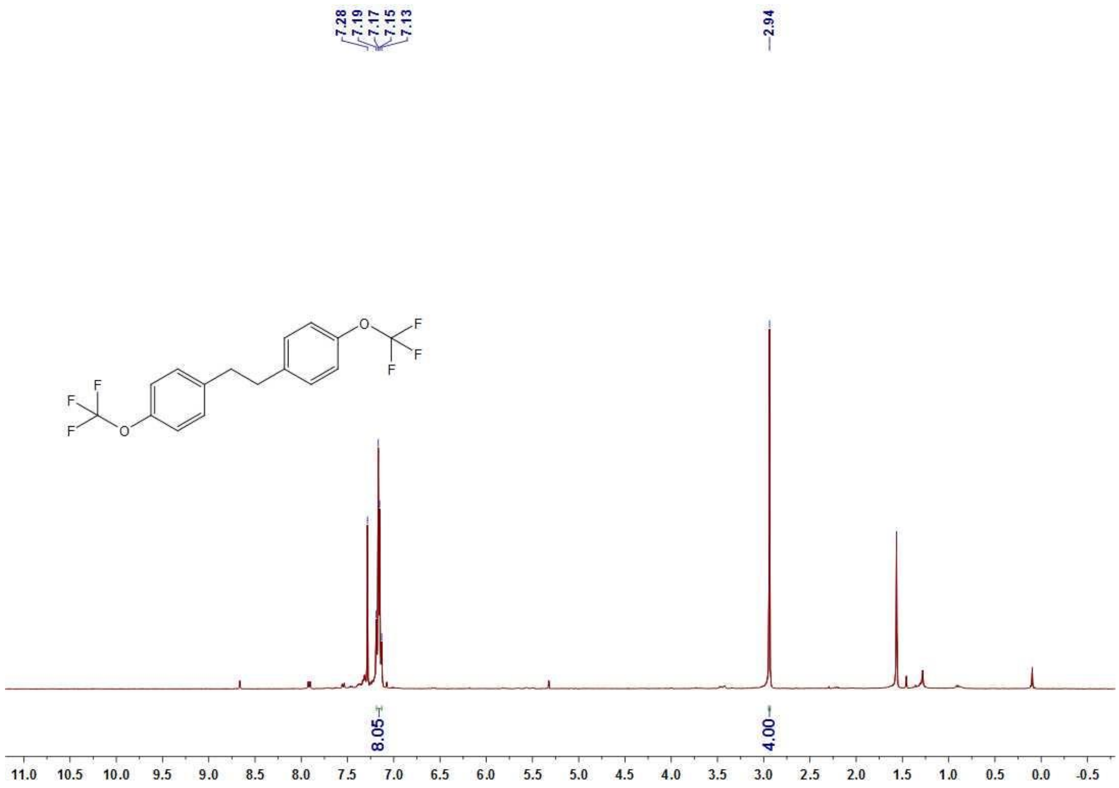

Supplementary Figure 30 ^{1}H NMR of **1,2-Bis(4-(trifluoromethoxy)phenyl)ethane**

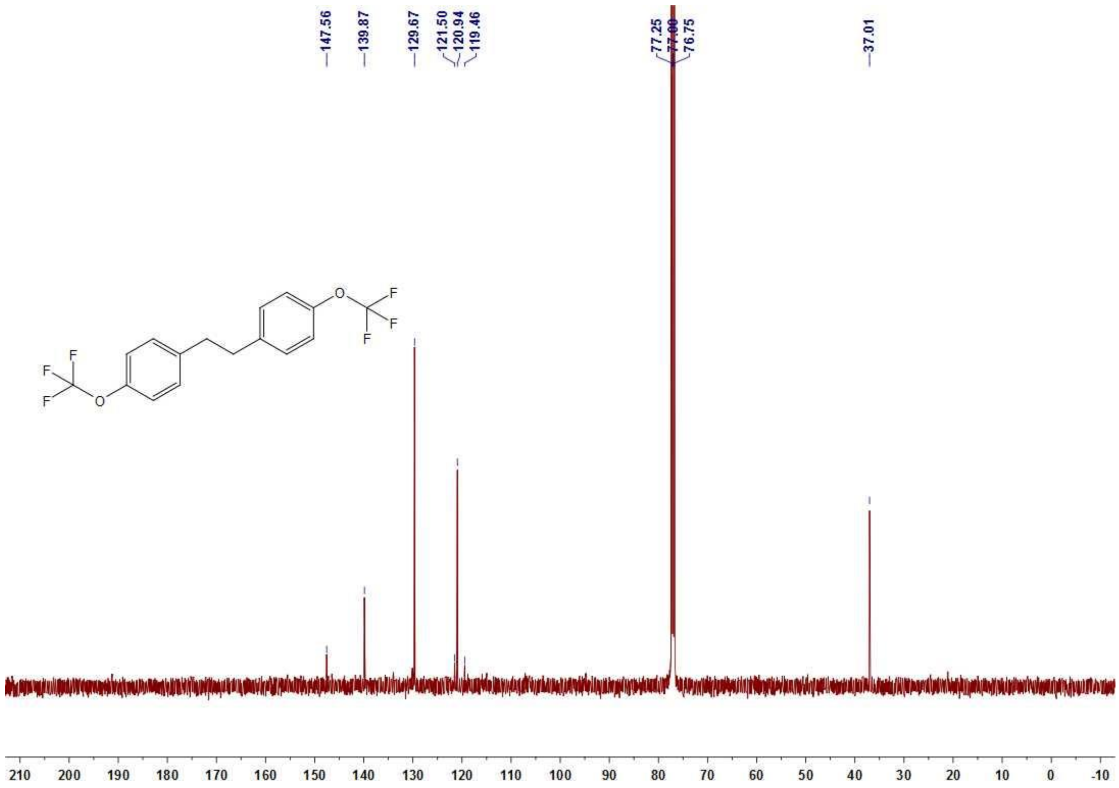

Supplementary Figure 31 ^{13}C NMR of **1,2-Bis(4-(trifluoromethoxy)phenyl)ethane**

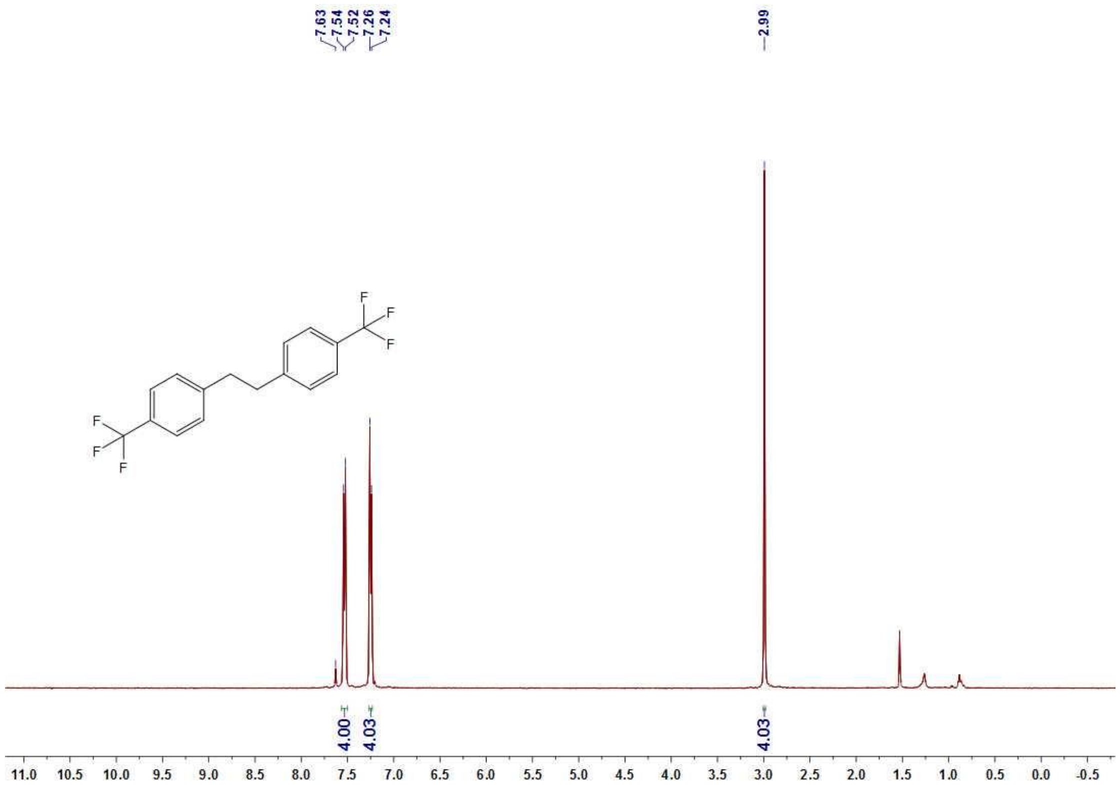

Supplementary Figure 32 ^{1}H NMR of **1,2-Bis(4-(trifluoromethyl)phenyl)ethane**

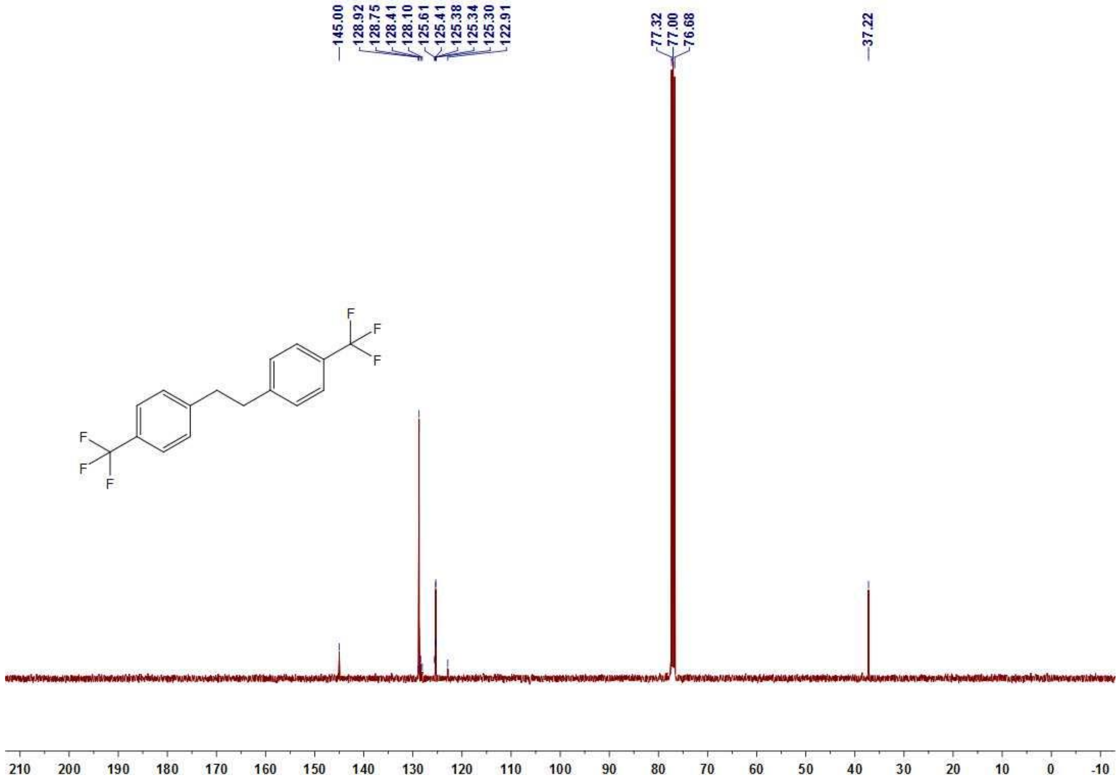

Supplementary Figure 33 ^{13}C NMR of **1,2-Bis(4-(trifluoromethyl)phenyl)ethane**

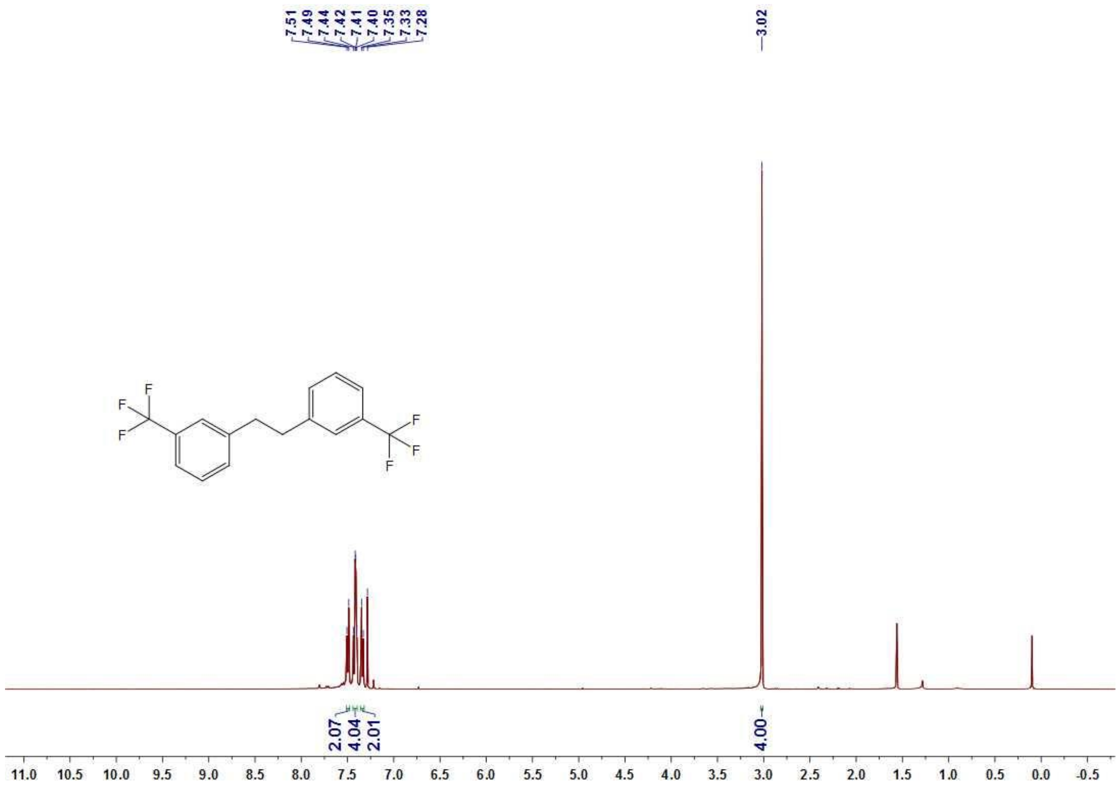

Supplementary Figure 34 ^{1}H NMR of **1,2-Bis(3-(trifluoromethyl)phenyl)ethane**

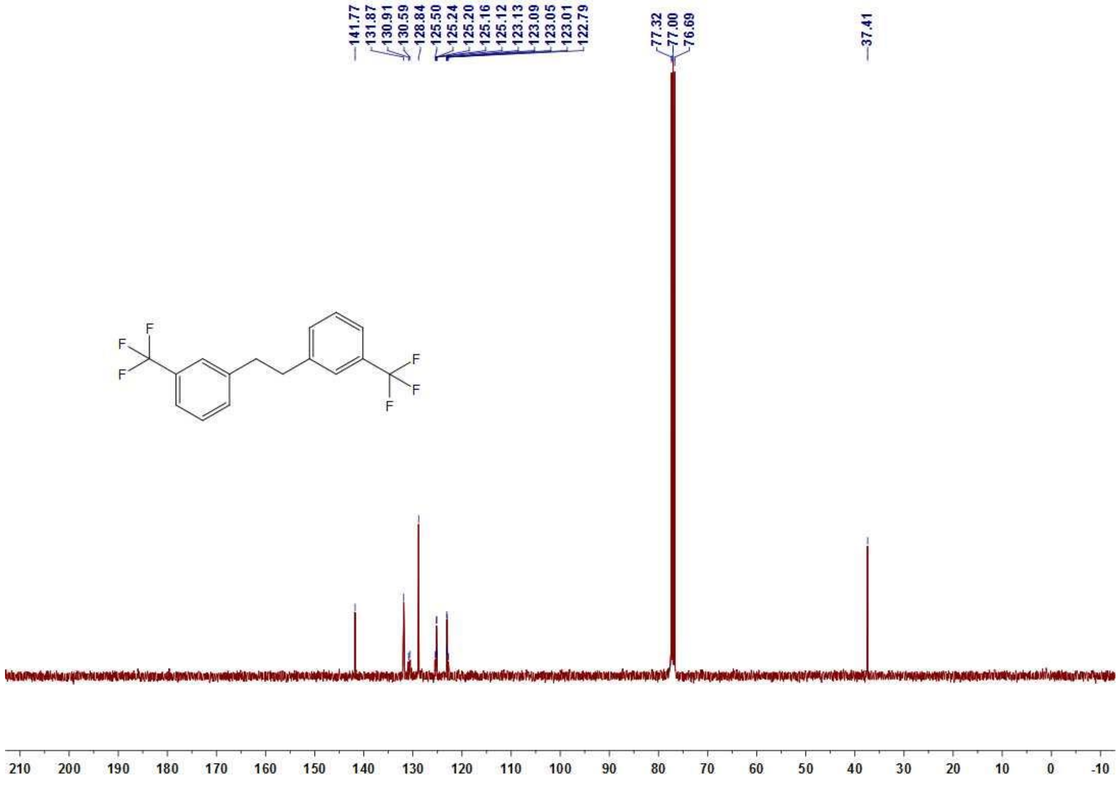

Supplementary Figure 35 ^{13}C NMR of **1,2-Bis(3-(trifluoromethyl)phenyl)ethane**

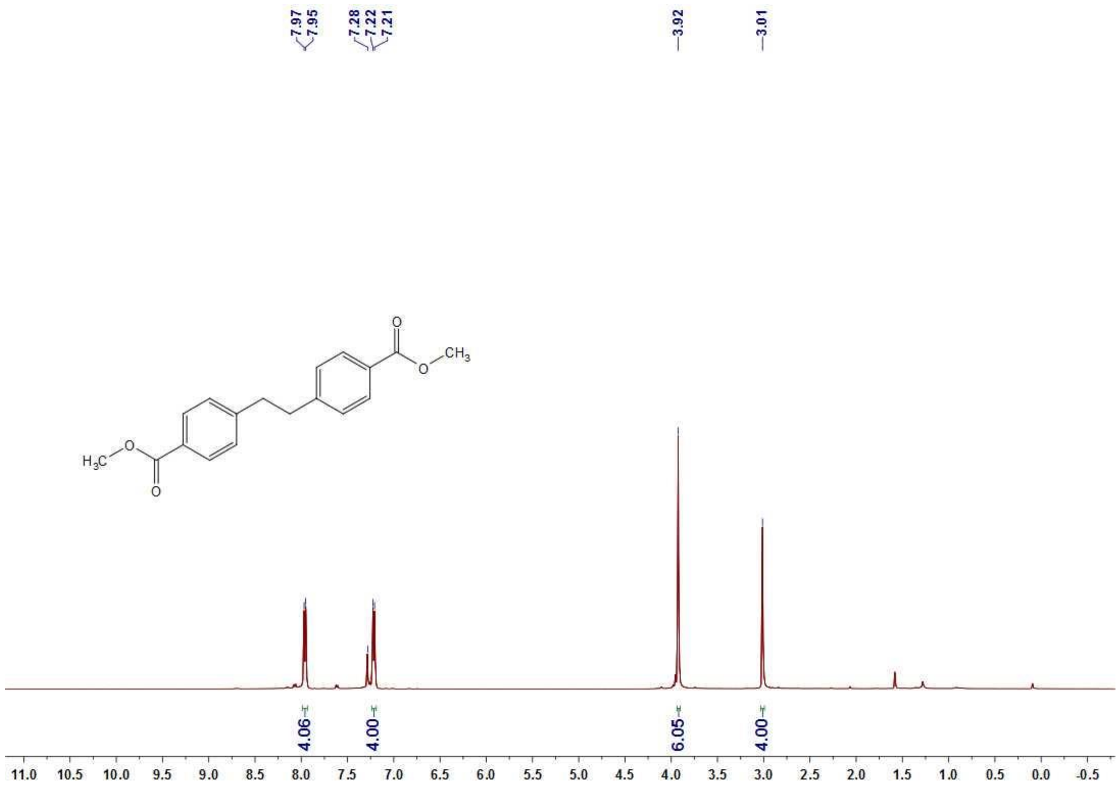

Supplementary Figure 36 ^{1}H NMR of **Dimethyl 4,4'-(ethane-1,2-diyl)dibenzoate**

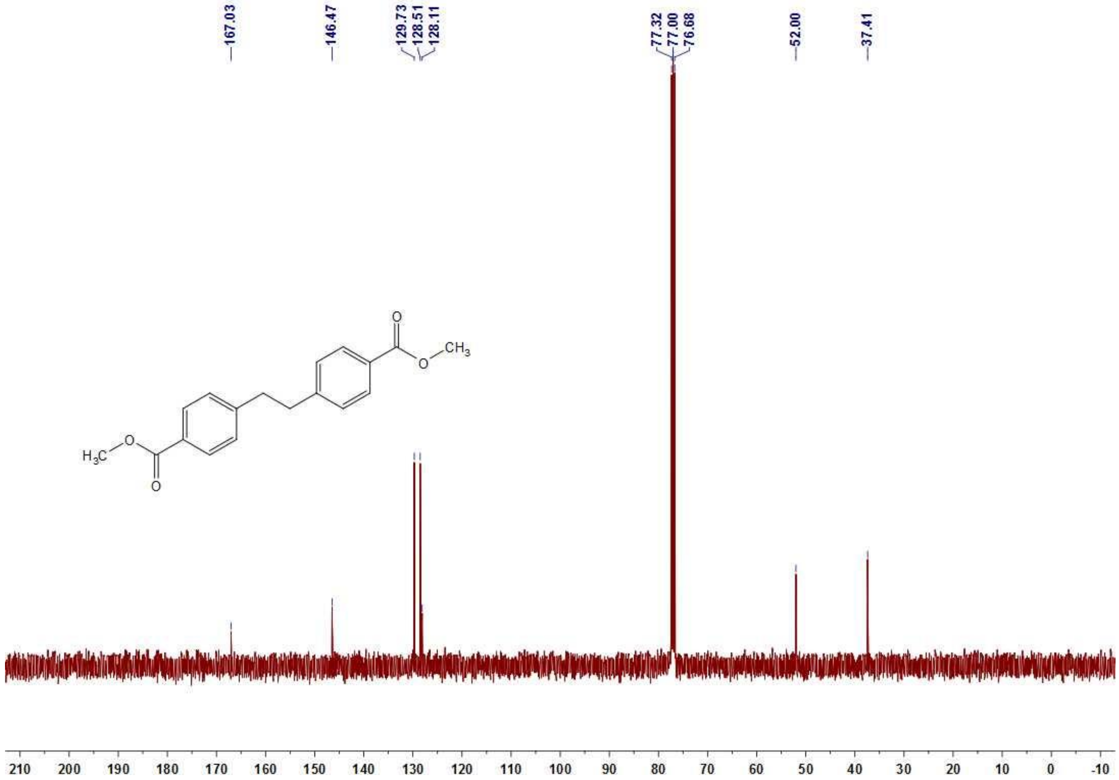

Supplementary Figure 37 ^{13}C NMR of **Dimethyl 4,4'-(ethane-1,2-diyl)dibenzoate**

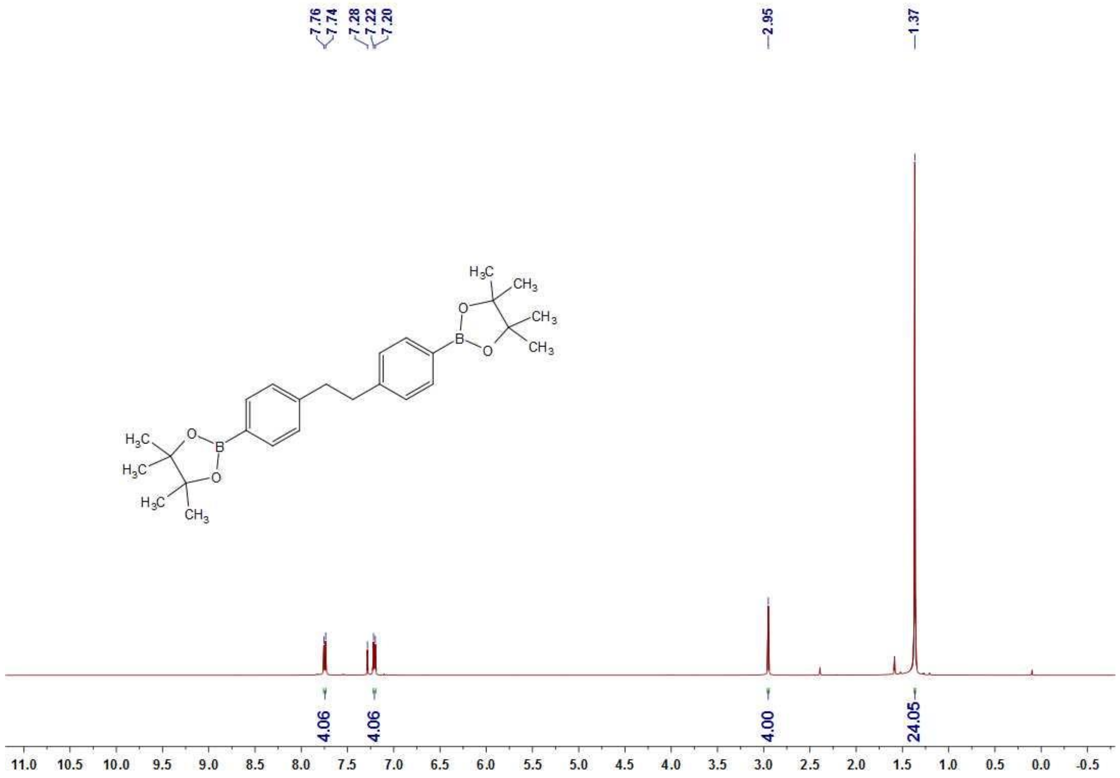

Supplementary Figure 38 ^{1}H NMR of

1,2-Bis(4-(4,4,5,5-tetramethyl-1,3,2-dioxaborolan-2-yl)phenyl)ethane

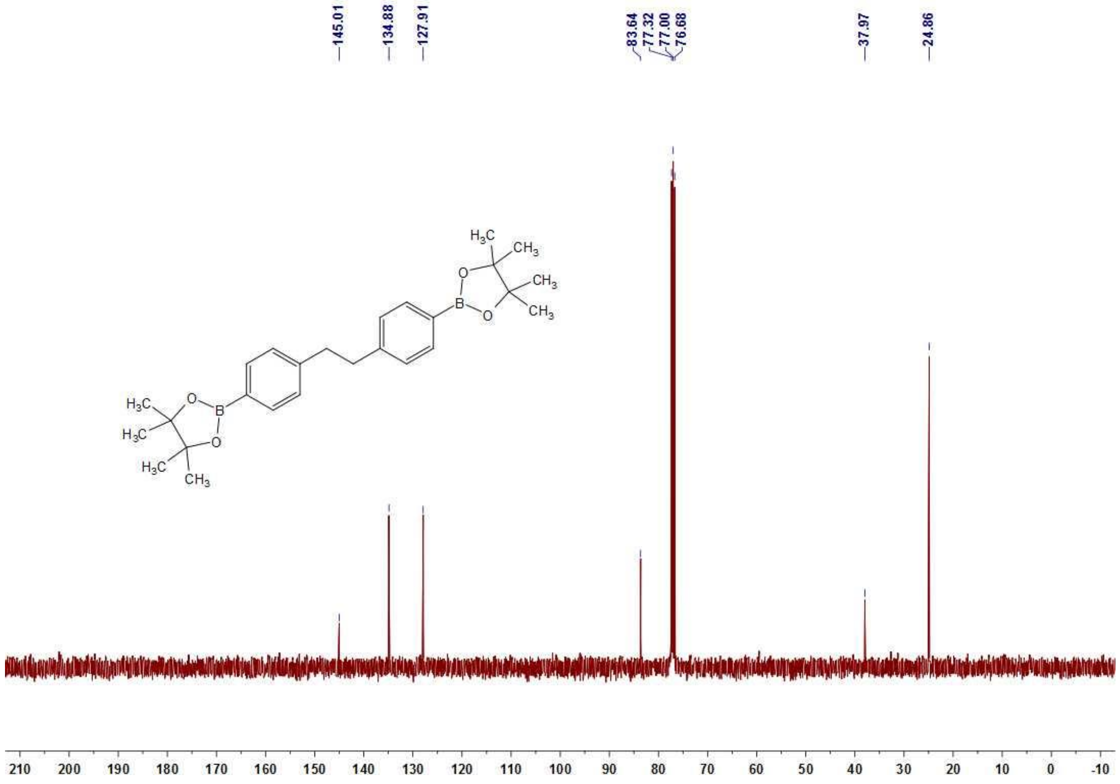

Supplementary Figure 39 ^{13}C NMR of

1,2-Bis(4-(4,4,5,5-tetramethyl-1,3,2-dioxaborolan-2-yl)phenyl)ethane

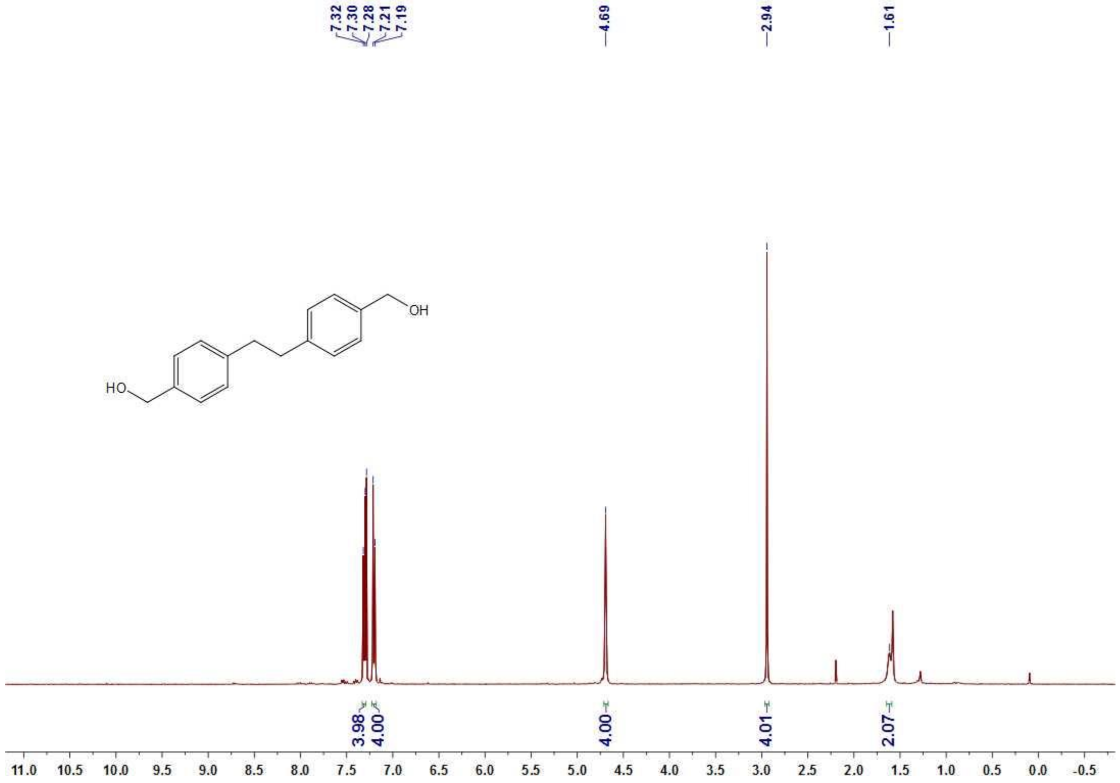

Supplementary Figure 40 ^{1}H NMR of **(Ethane-1,2-diylbis(4,1-phenylene))dimethanol**

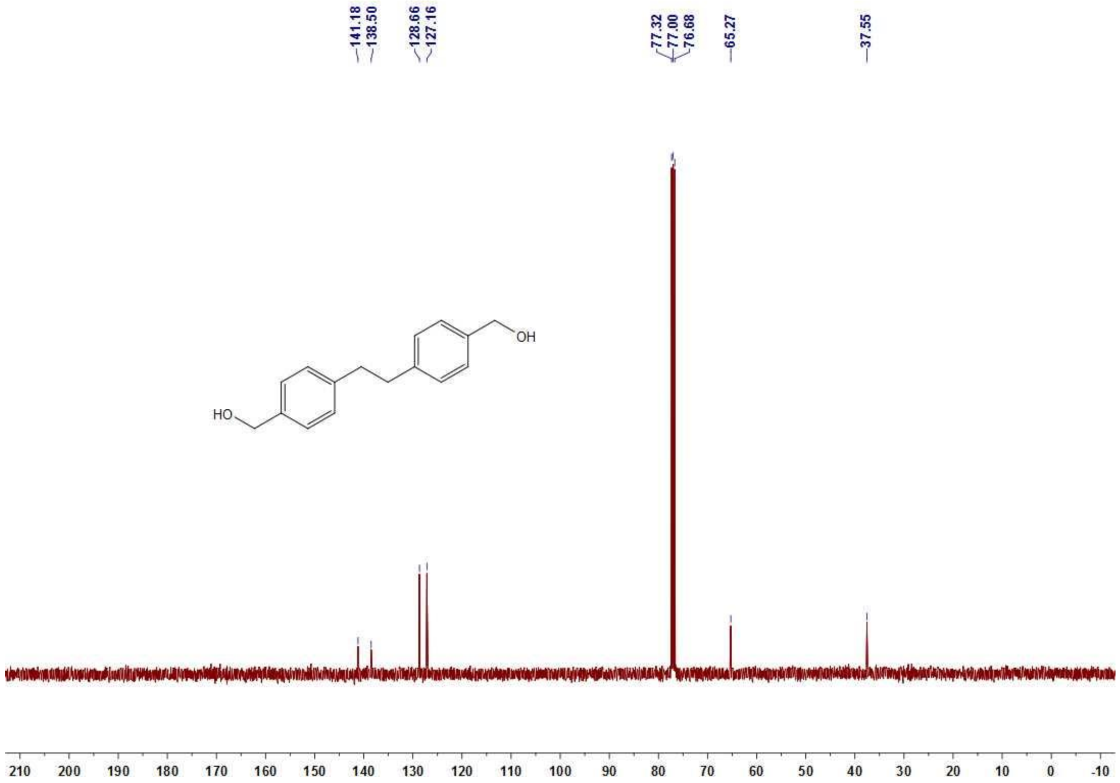

Supplementary Figure 41 ^{13}C NMR of **(Ethane-1,2-diylbis(4,1-phenylene))dimethanol**

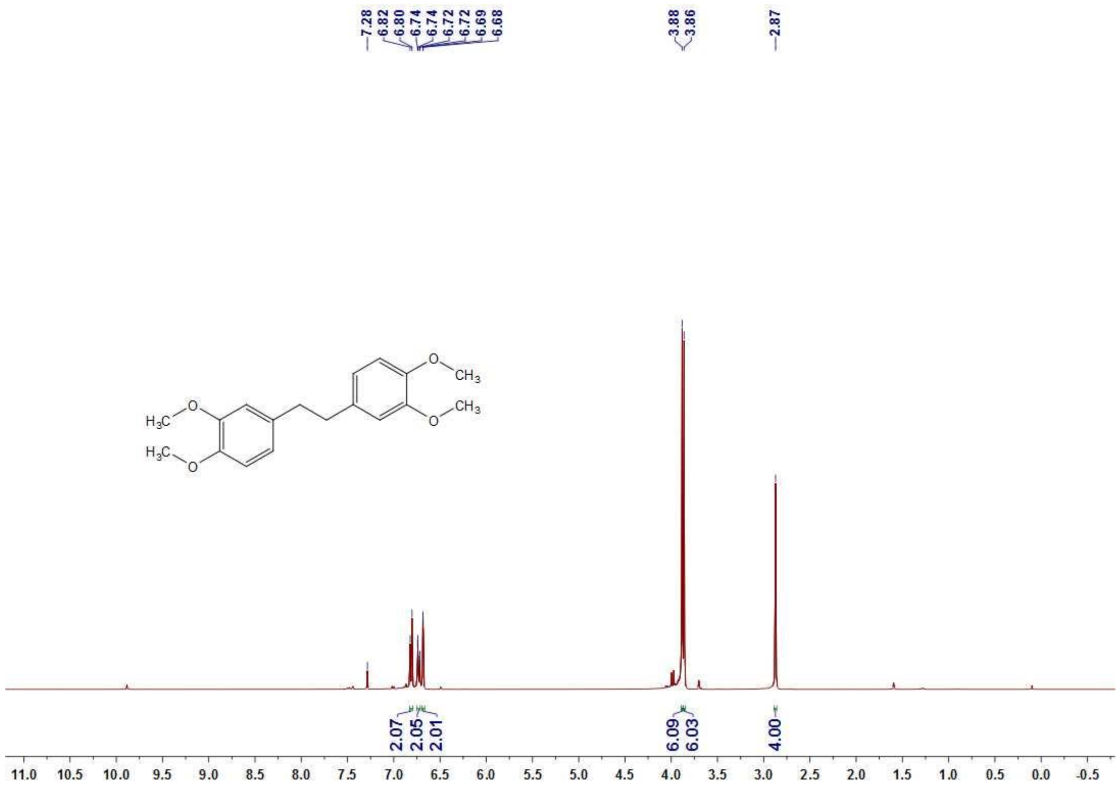

Supplementary Figure 42 ^{1}H NMR of **1,2-Bis(3,4-dimethoxyphenyl)ethane**

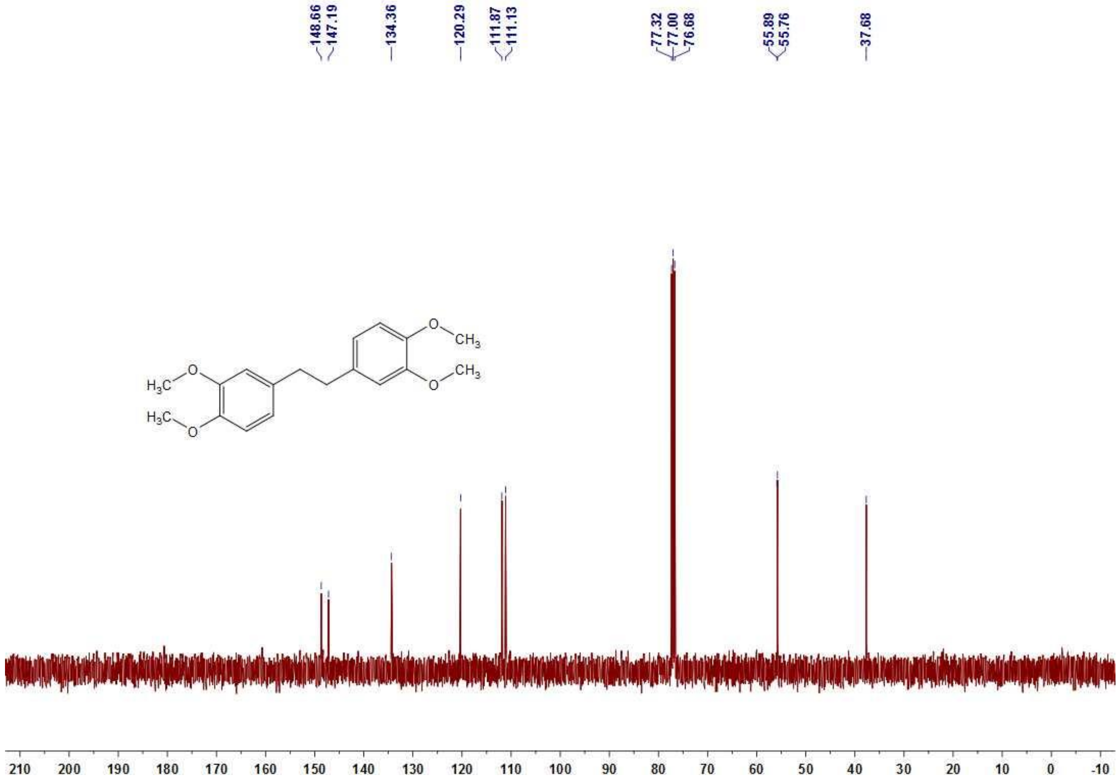

Supplementary Figure 43 ^{13}C NMR of **1,2-Bis(3,4-dimethoxyphenyl)ethane**

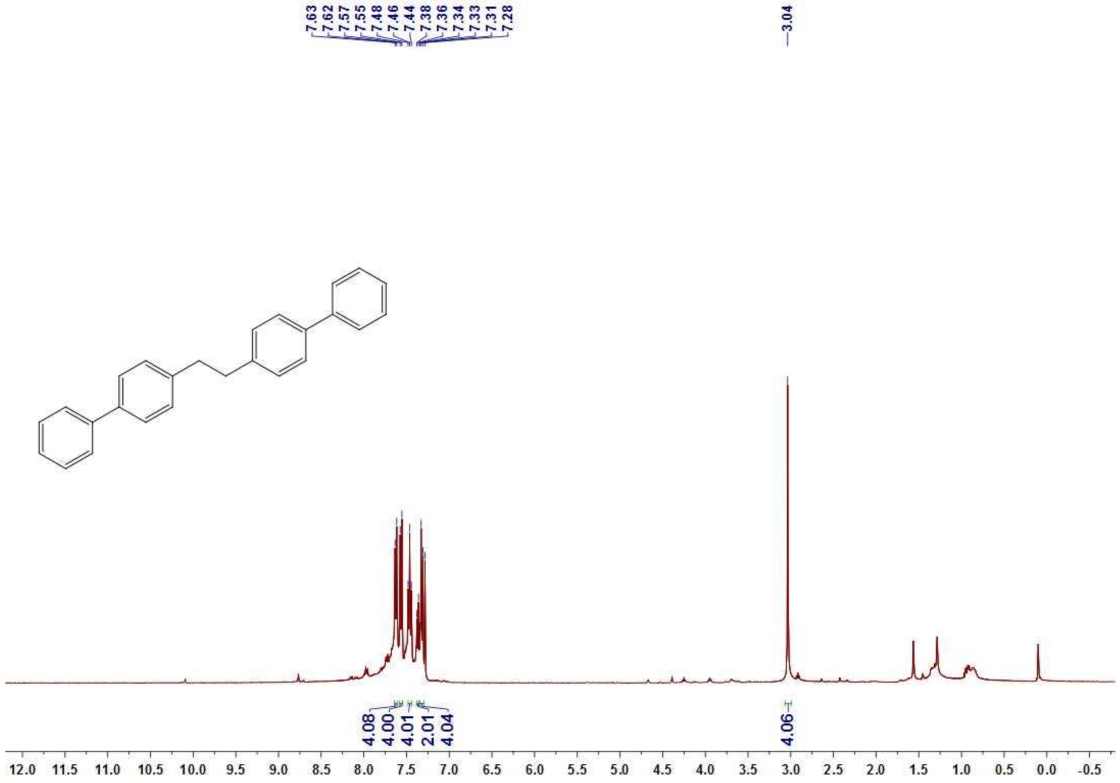

Supplementary Figure 44 ^{1}H NMR of **1,2-Di([1,1'-biphenyl]-4-yl)ethane**

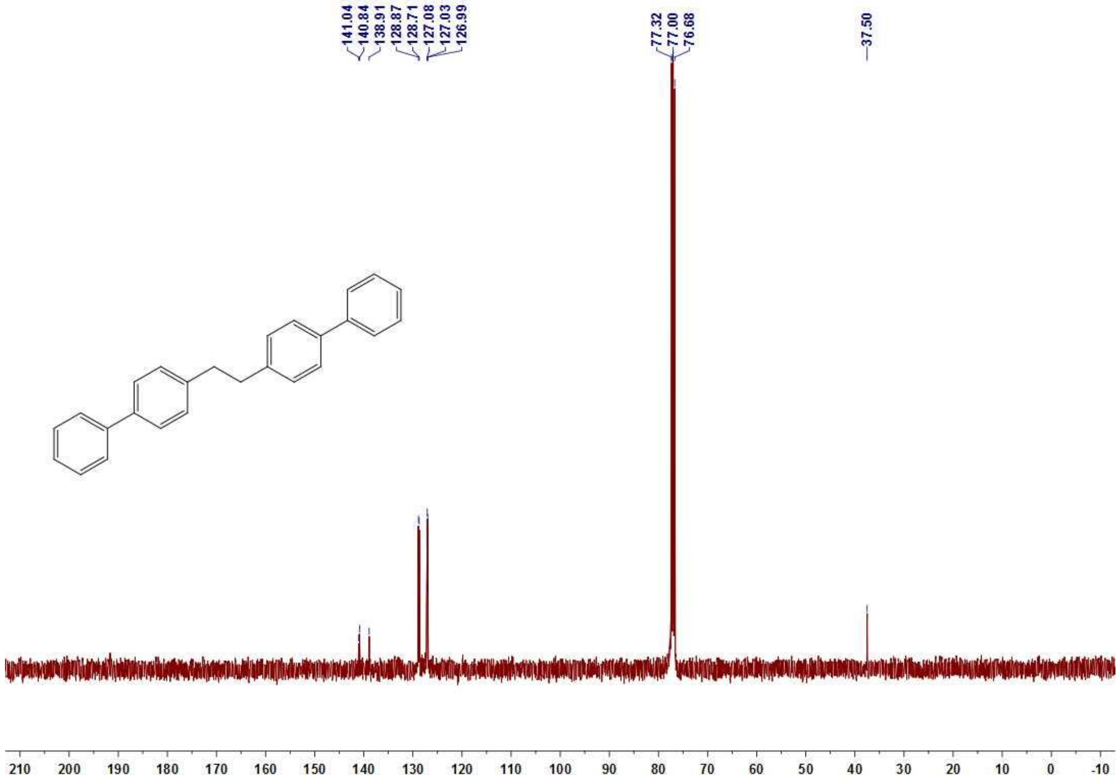

Supplementary Figure 45 ^{13}C NMR of **1,2-Di([1,1'-biphenyl]-4-yl)ethane**

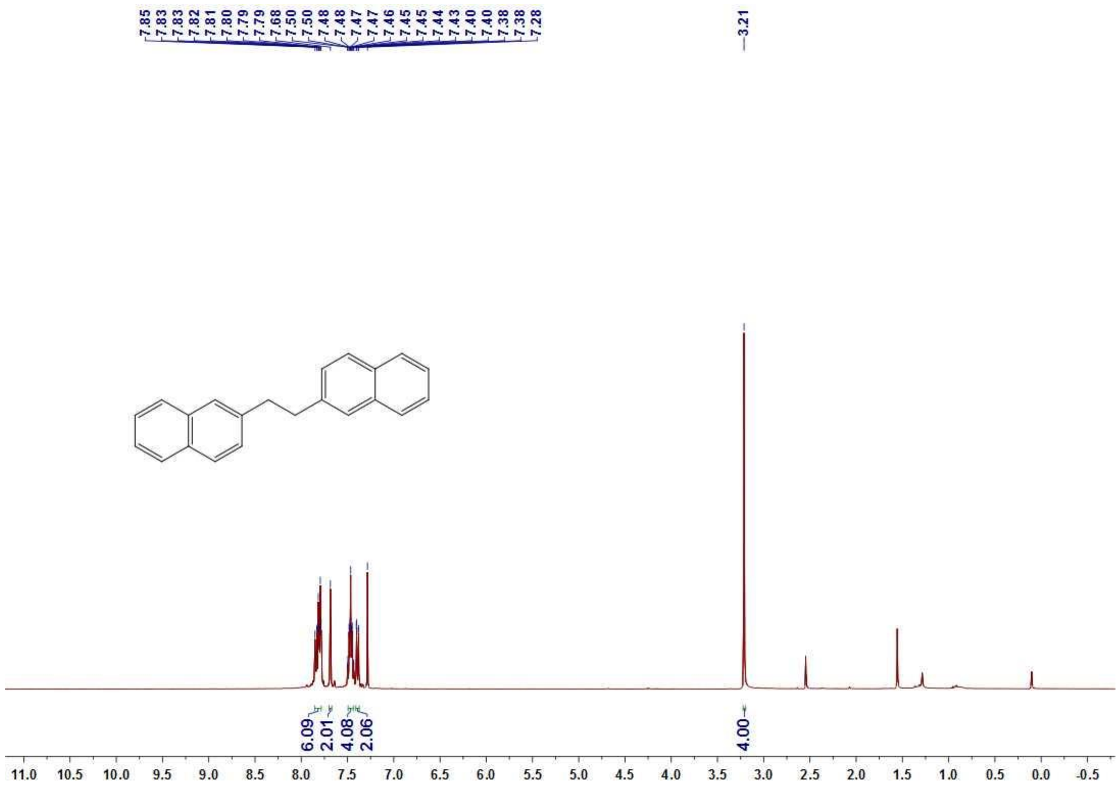

Supplementary Figure 46 ^{1}H NMR of **1,2-Di(naphthalen-2-yl)ethane**

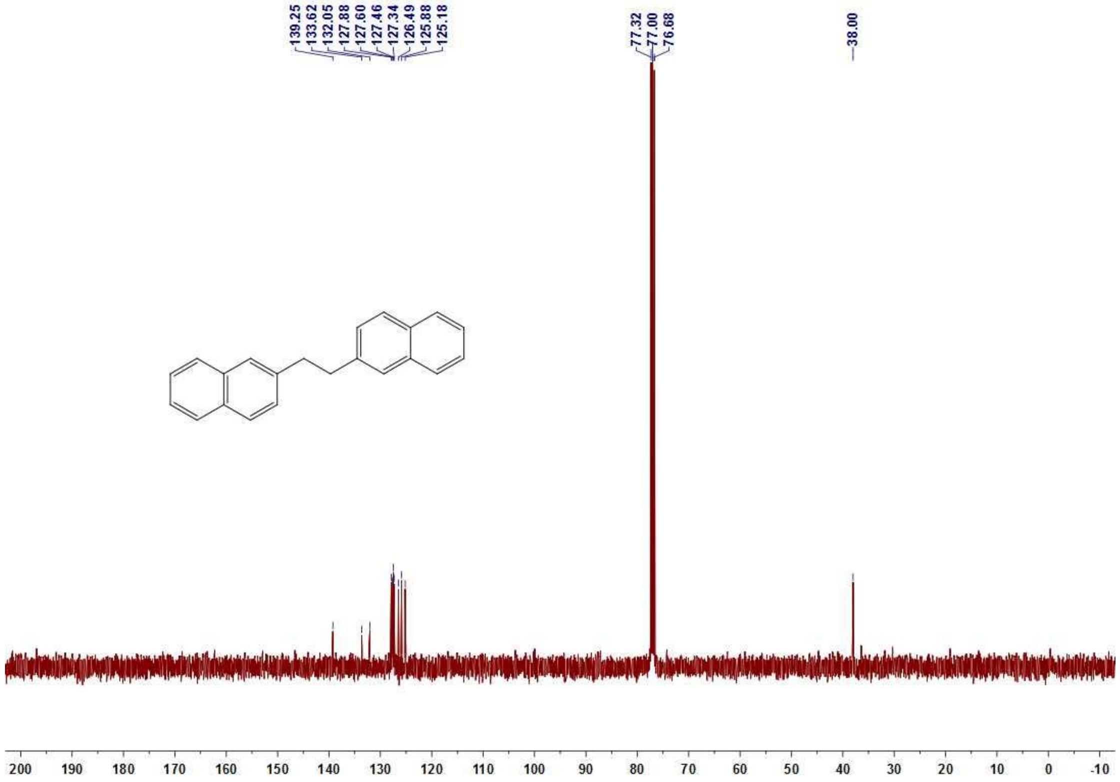

Supplementary Figure 47 ^{13}C NMR of **1,2-Di(naphthalen-2-yl)ethane**

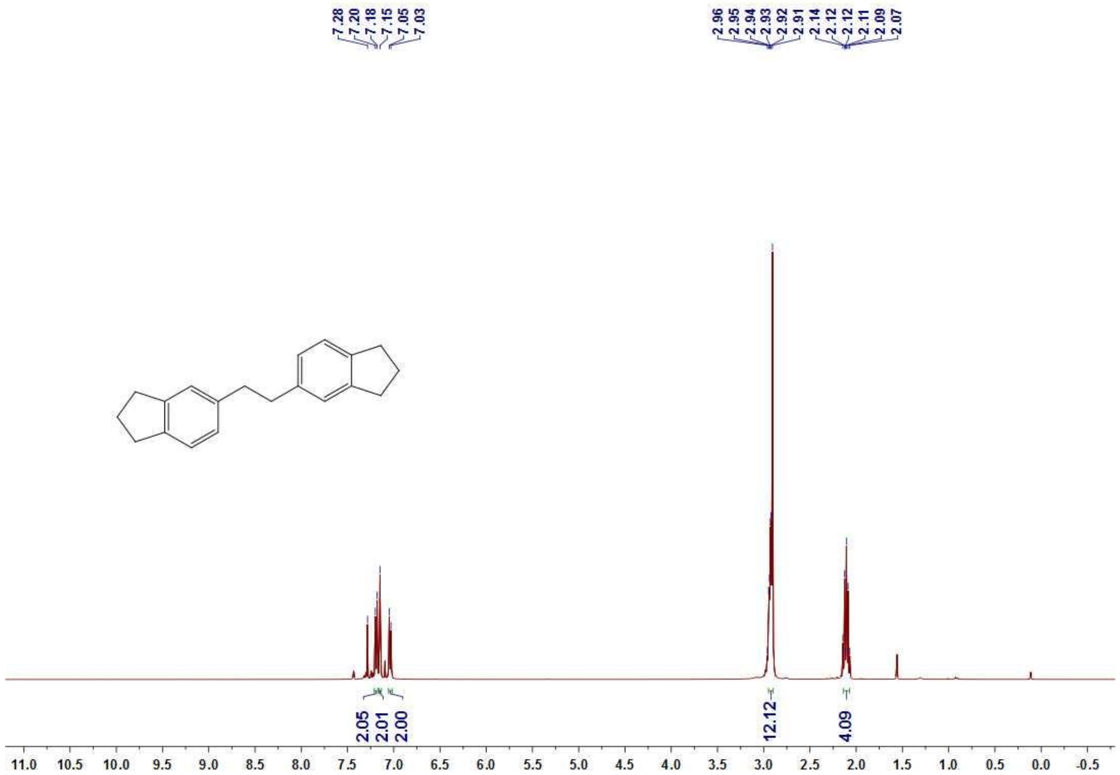

Supplementary Figure 48 ^{1}H NMR of **1,2-Bis(2,3-dihydro-1H-inden-5-yl)ethane**

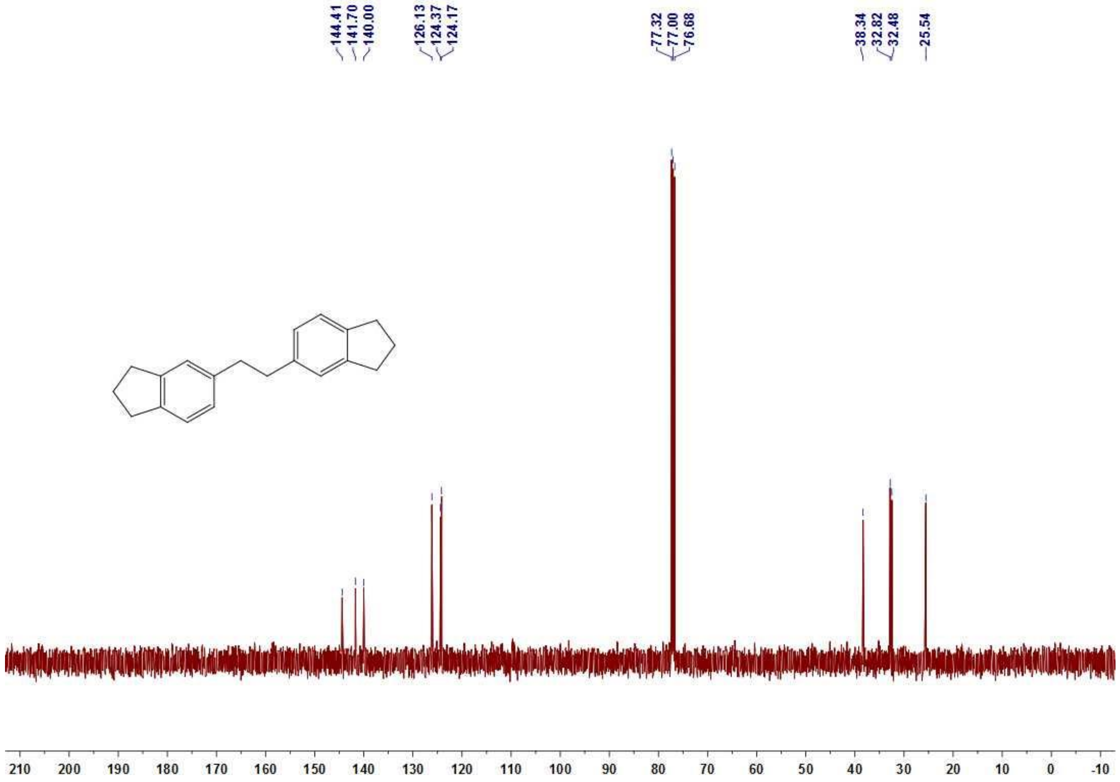

Supplementary Figure 49 ^{13}C NMR of **1,2-Bis(2,3-dihydro-1H-inden-5-yl)ethane**

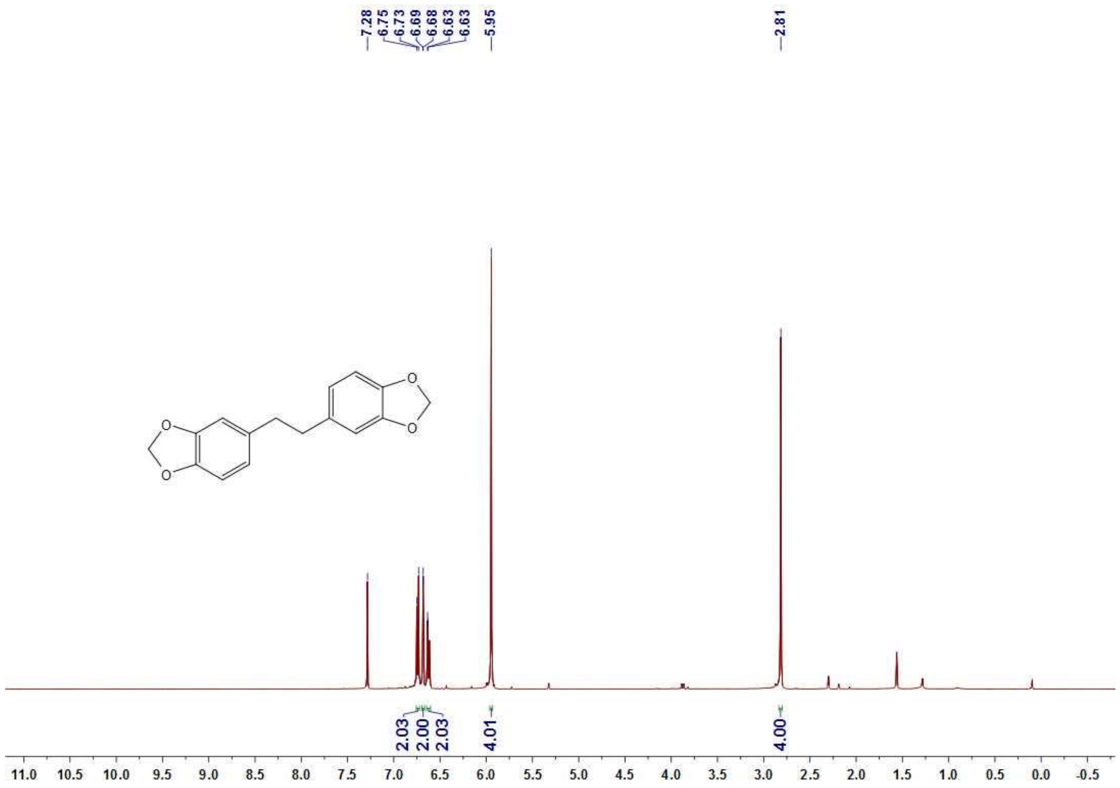

Supplementary Figure 50 ^{1}H NMR of **1,2-Bis(benzo[d][1,3]dioxol-5-yl)ethane**

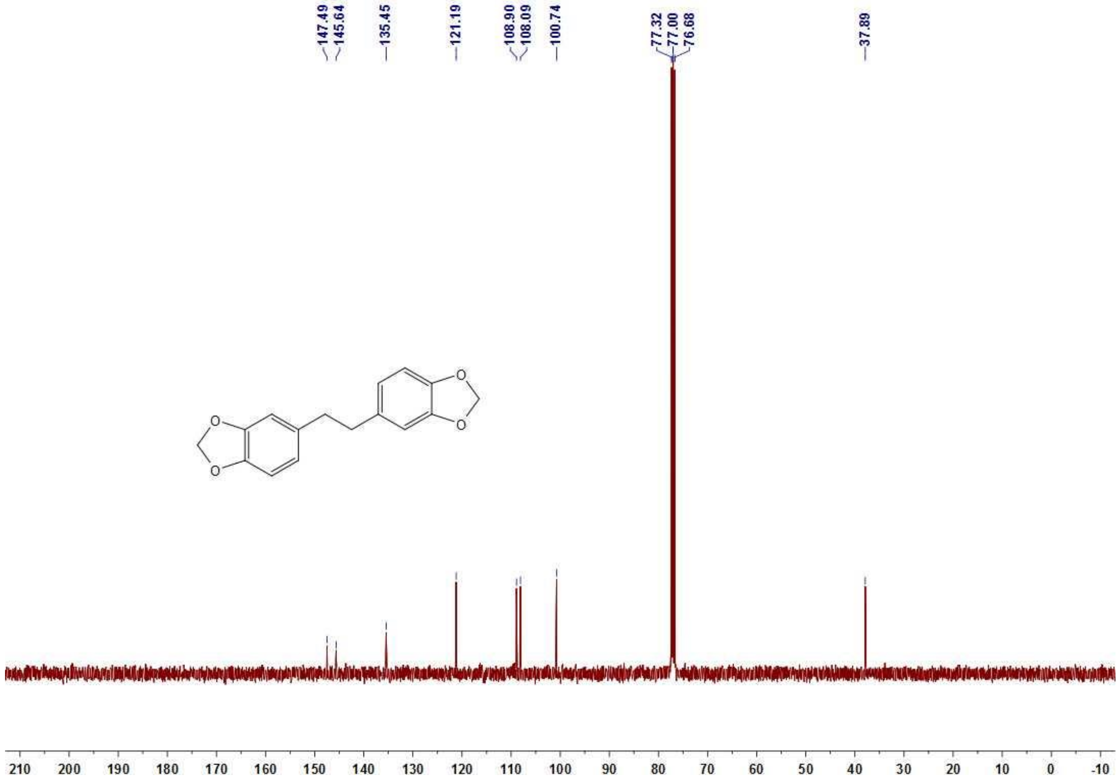

Supplementary Figure 51 ^{13}C NMR of **1,2-Bis(benzo[d][1,3]dioxol-5-yl)ethane**

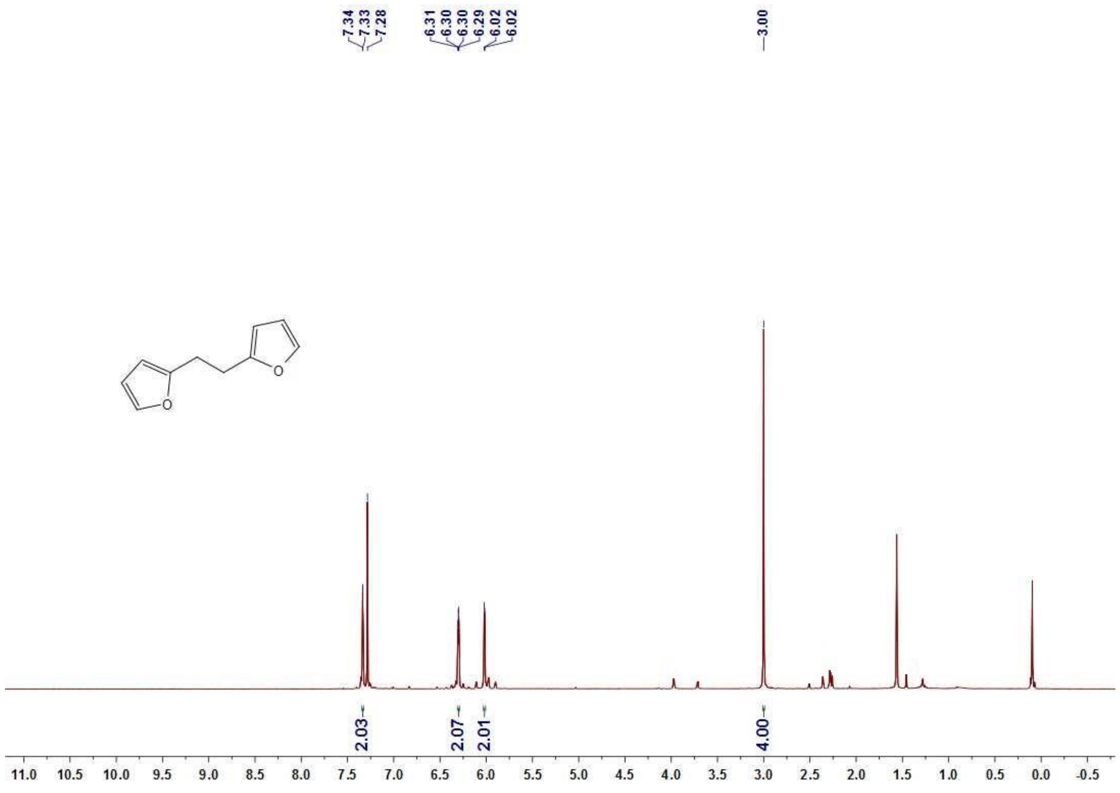

Supplementary Figure 52 ^{1}H NMR of **1,2-Di(furan-2-yl)ethane**

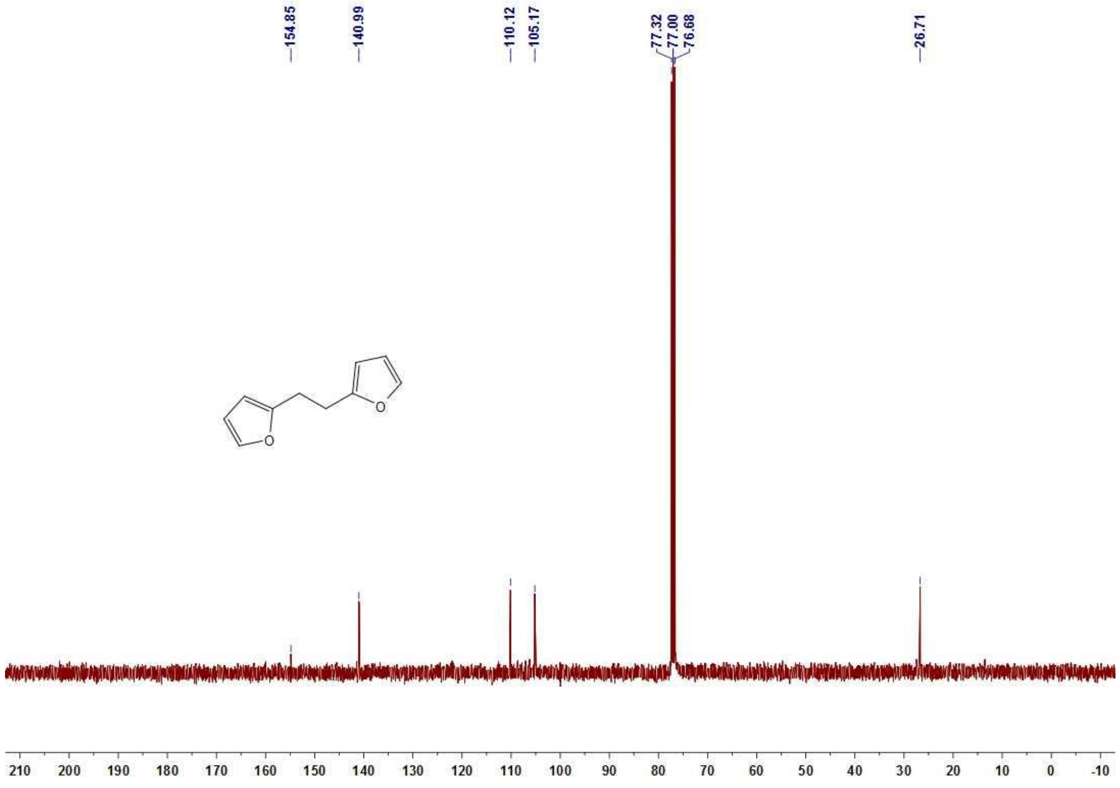

Supplementary Figure 53 ^{13}C NMR of **1,2-Di(furan-2-yl)ethane**

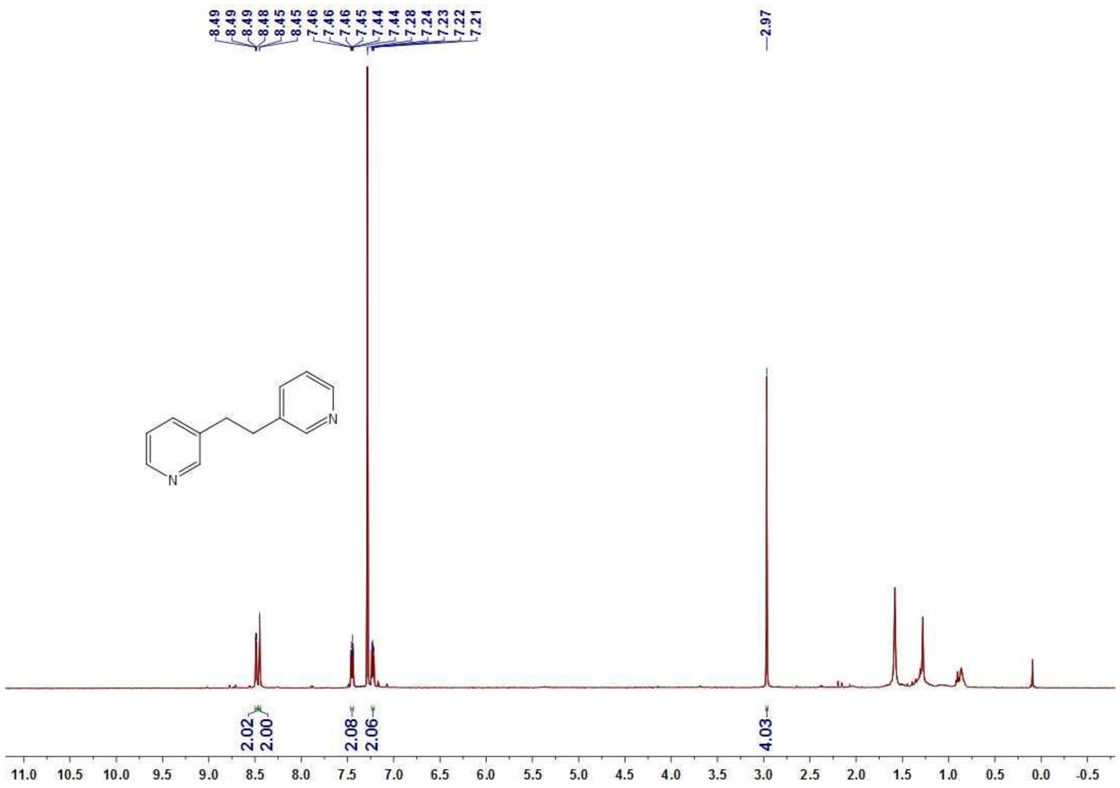

Supplementary Figure 54 ^{1}H NMR of **1,2-Di(pyridin-3-yl)ethane**

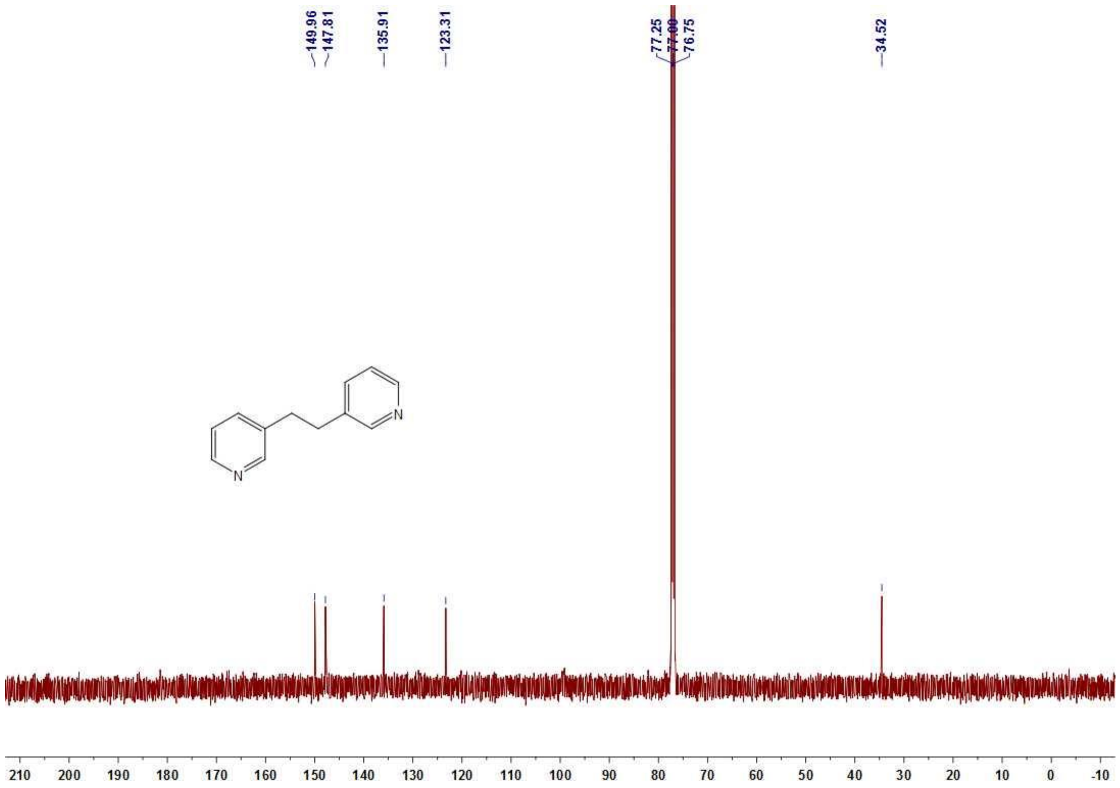

Supplementary Figure 55 ^{13}C NMR of **1,2-Di(pyridin-3-yl)ethane**

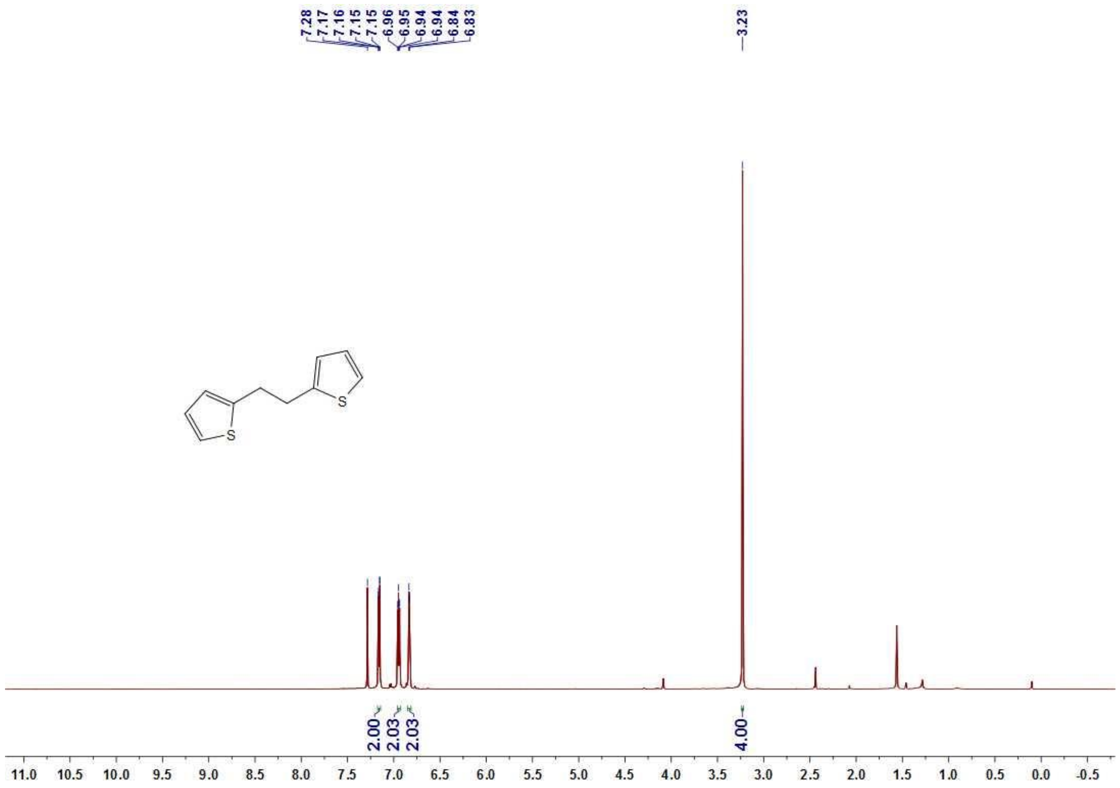

Supplementary Figure 56 ^{1}H NMR of **1,2-Di(thiophen-2-yl)ethane**

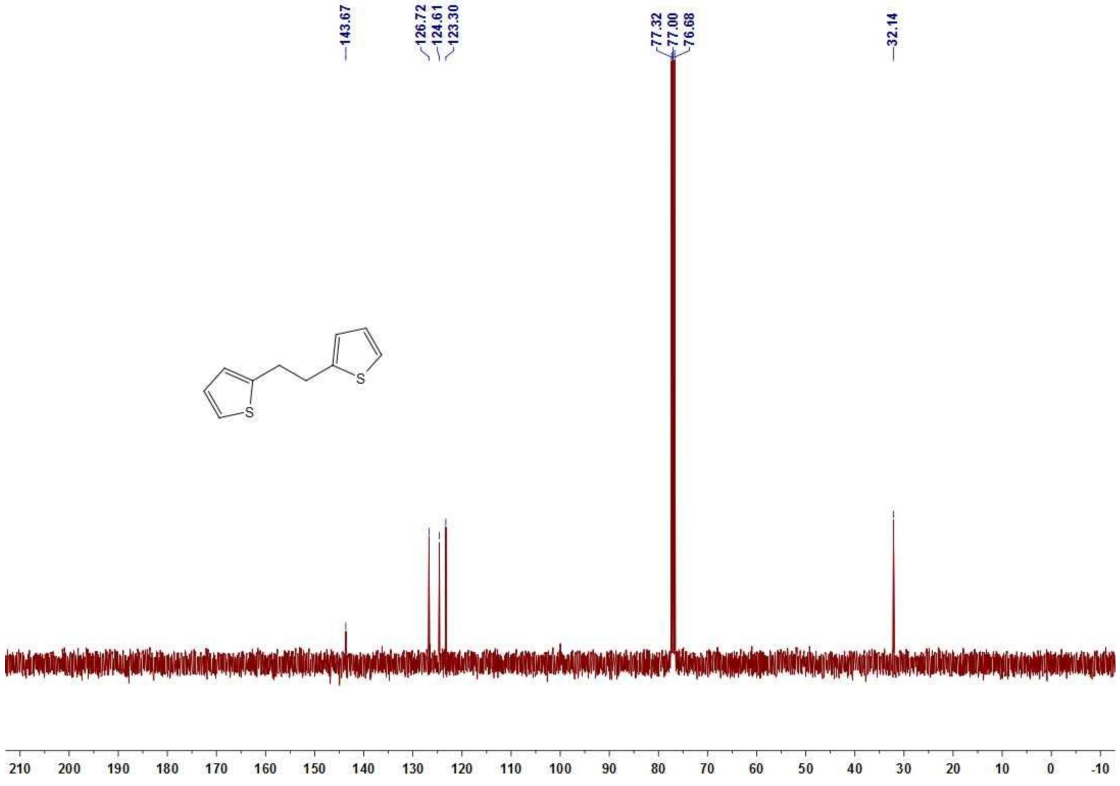

Supplementary Figure 57 ^{13}C NMR of **1,2-Di(thiophen-2-yl)ethane**

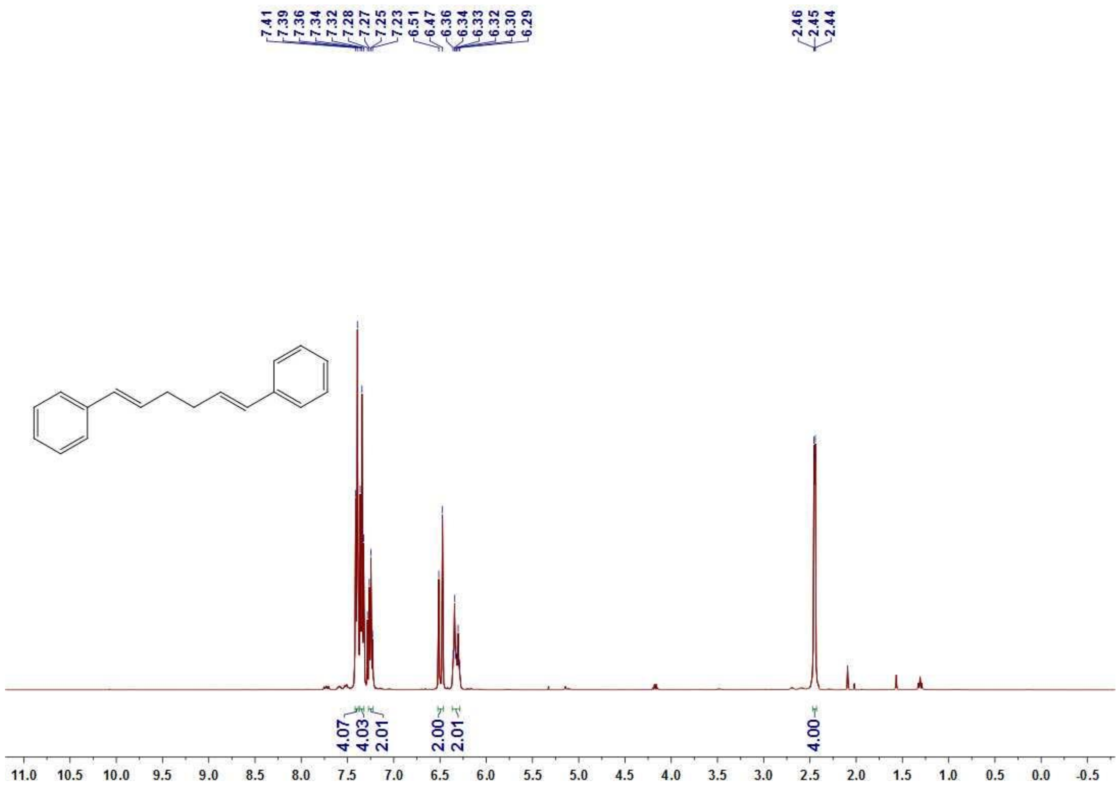

Supplementary Figure 58 ^{1}H NMR of **(1E,5E)-1,6-Diphenylhexa-1,5-diene**

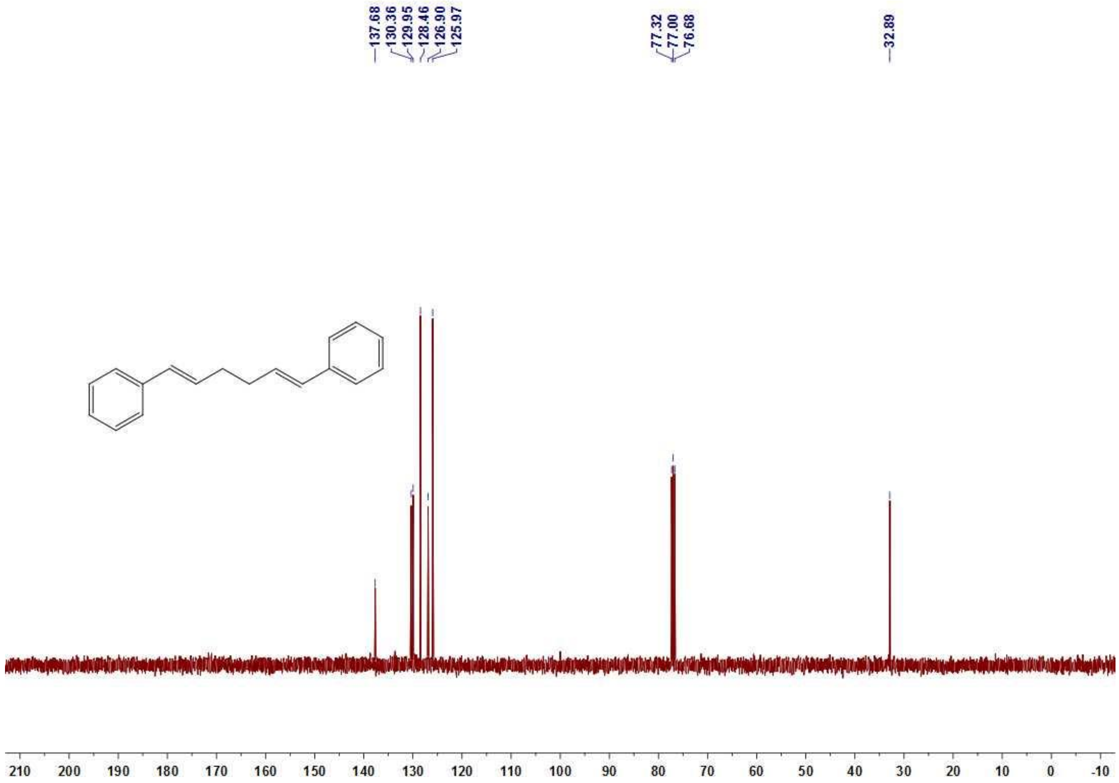

Supplementary Figure 59 ^{13}C NMR of **(1E,5E)-1,6-Diphenylhexa-1,5-diene**

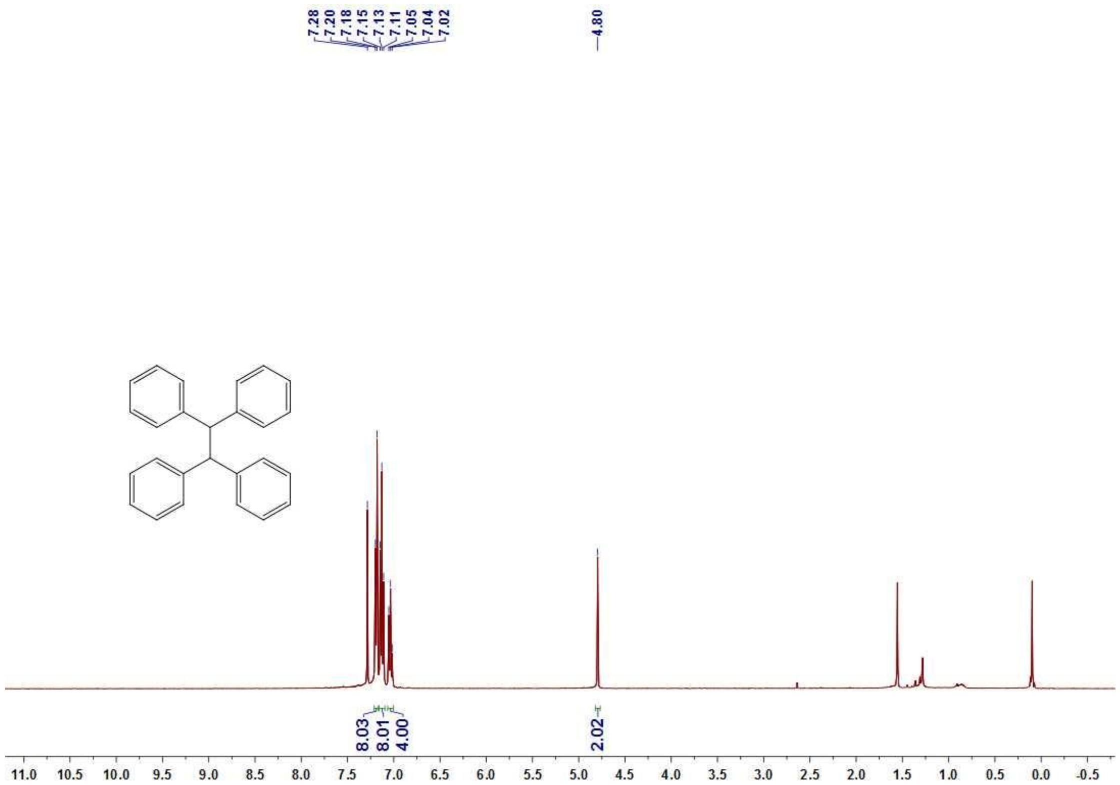

Supplementary Figure 60 ^{1}H NMR of **1,1,2,2-Tetraphenylethane**

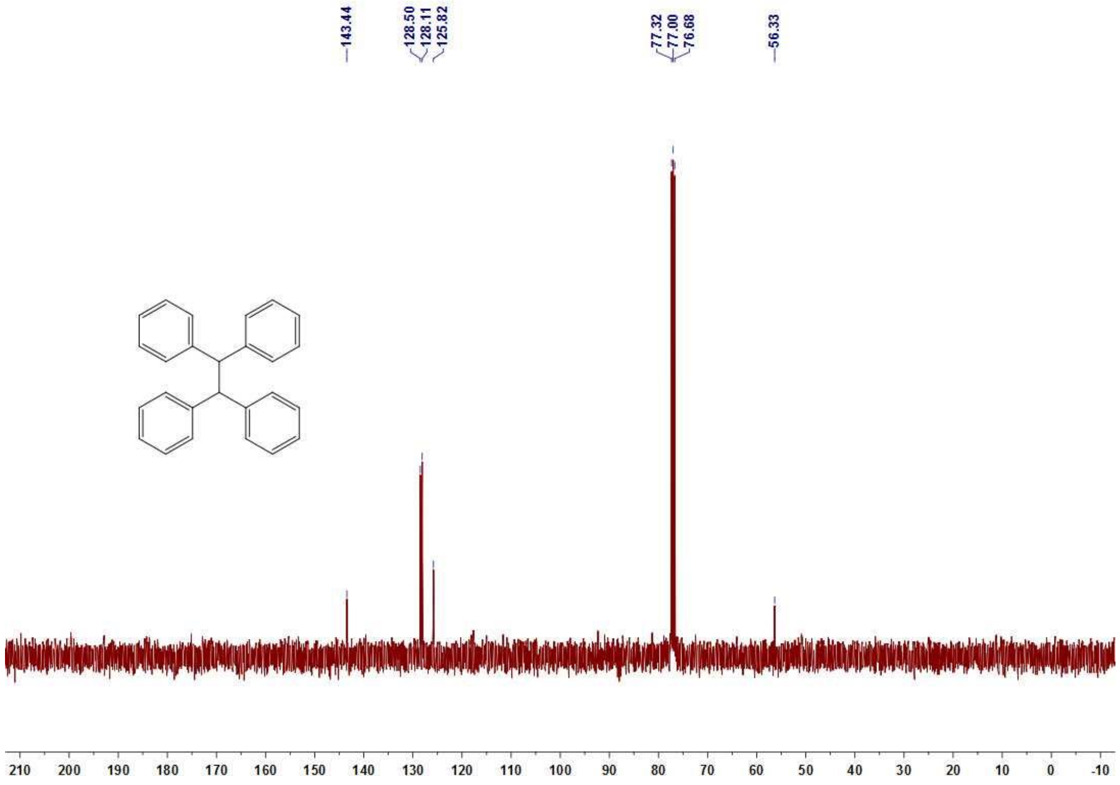

Supplementary Figure 61 ^{13}C NMR of **1,1,2,2-Tetraphenylethane**

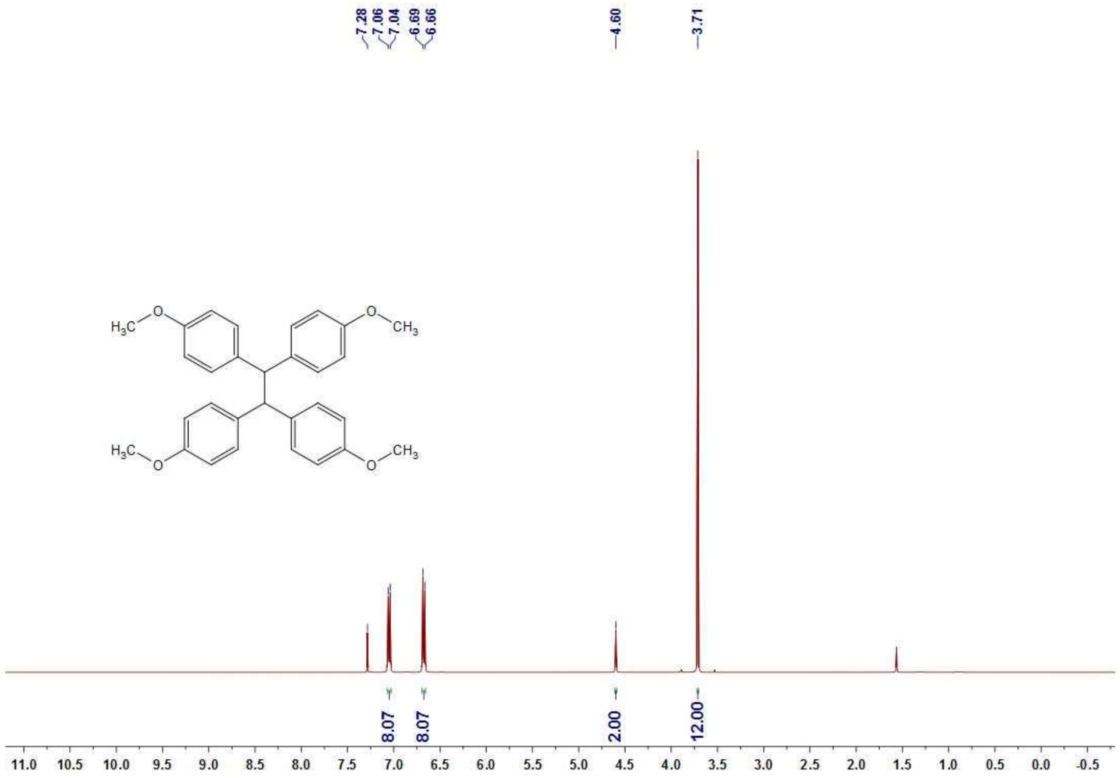

Supplementary Figure 62 ^{1}H NMR of **1,1,2,2-Tetrakis(4-methoxyphenyl)ethane**

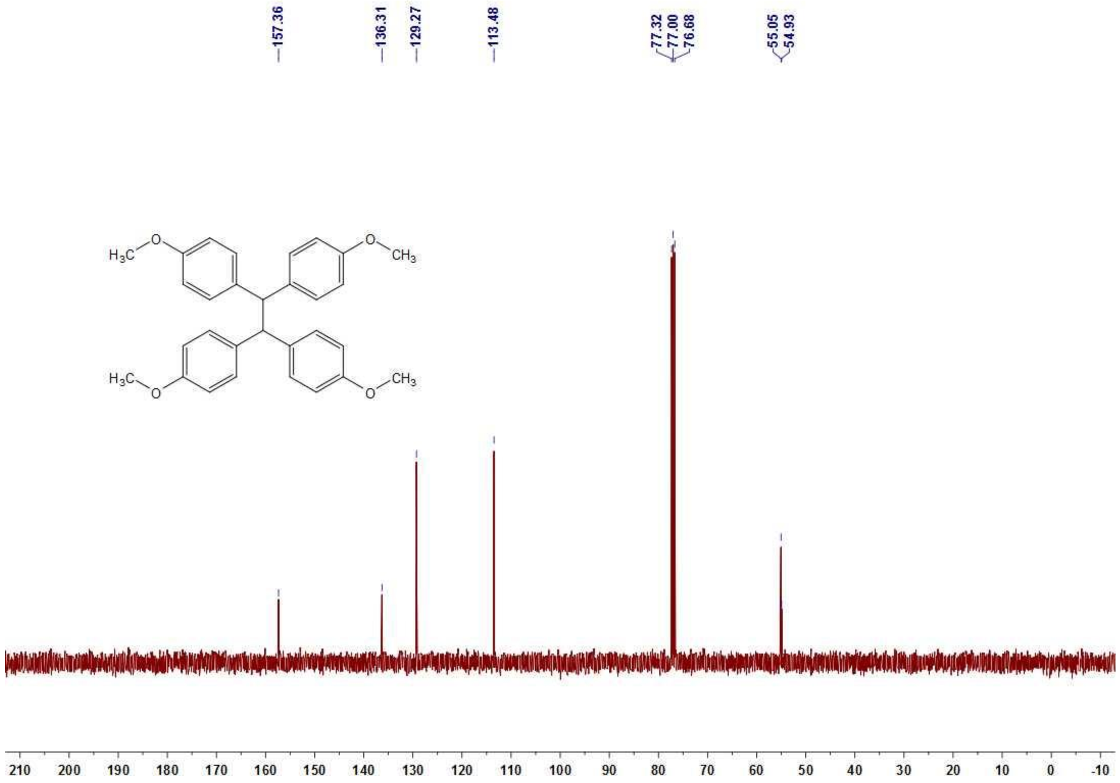

Supplementary Figure 63 ^{13}C NMR of **1,1,2,2-Tetrakis(4-methoxyphenyl)ethane**

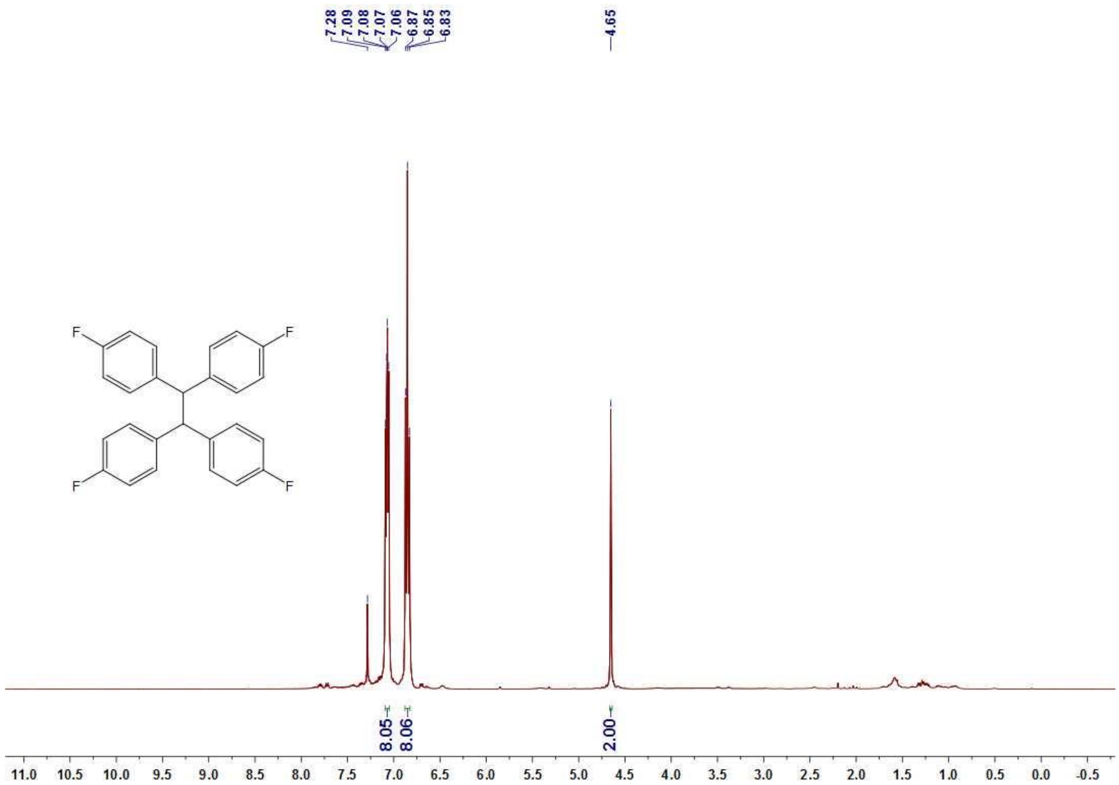

Supplementary Figure 64 ^{1}H NMR of **1,1,2,2-Tetrakis(4-fluorophenyl)ethane**

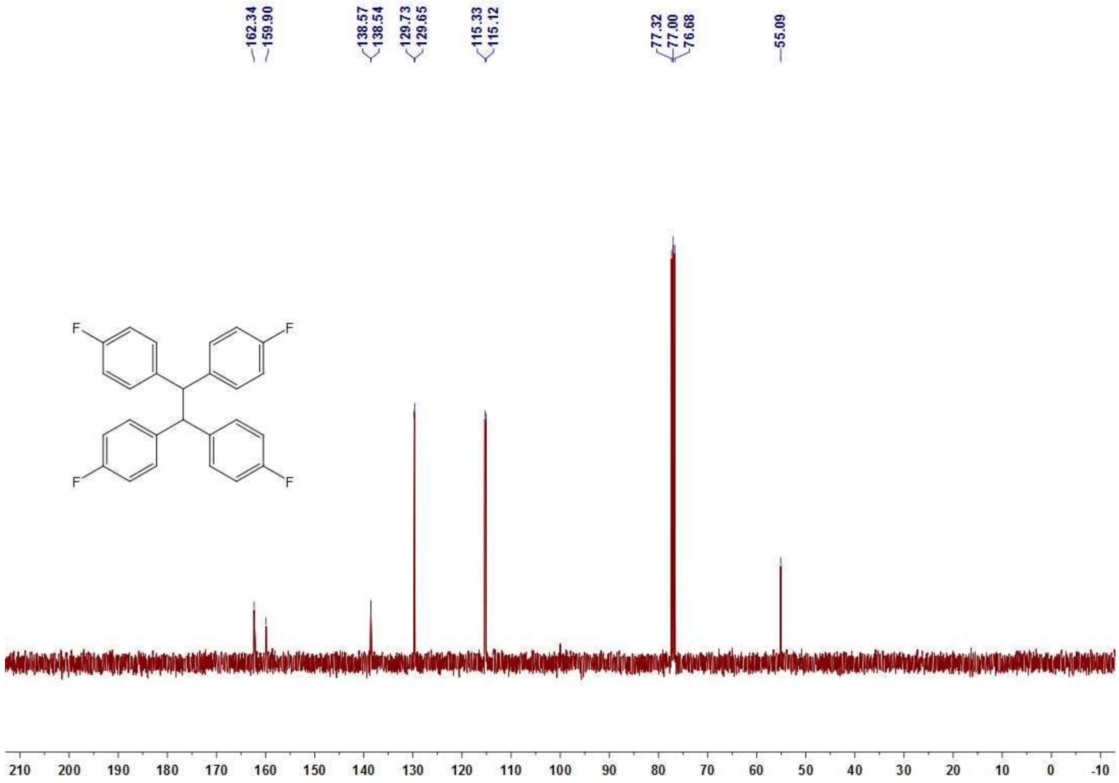

Supplementary Figure 65 ^{13}C NMR of **1,1,2,2-Tetrakis(4-fluorophenyl)ethane**

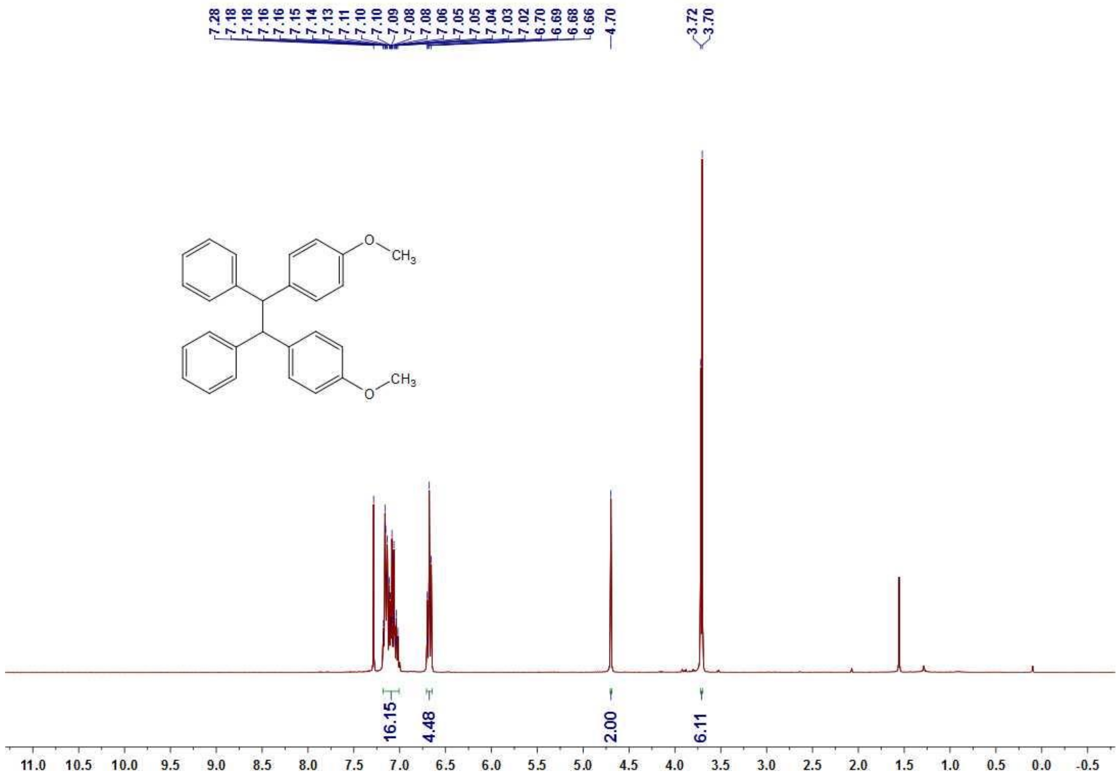

Supplementary Figure 66 ^{1}H NMR of **1,2-Bis(4-methoxyphenyl)-1,2-diphenylethane**

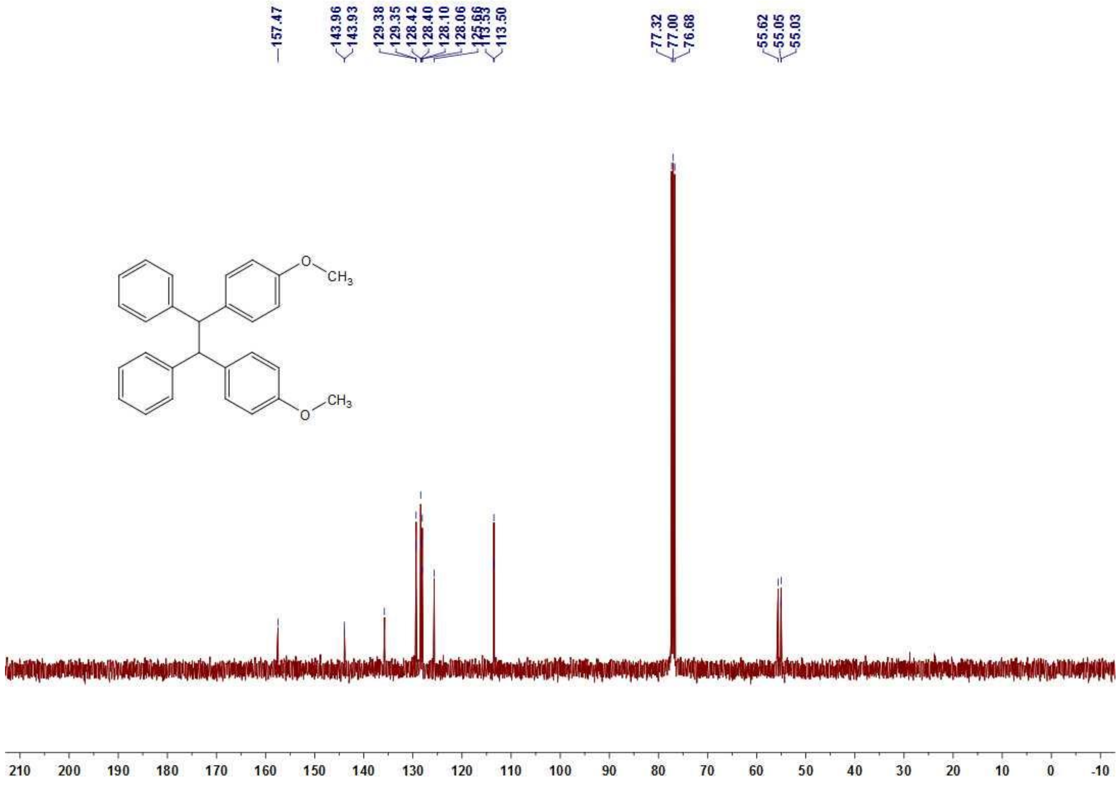

Supplementary Figure 67 ^{13}C NMR of **1,2-Bis(4-methoxyphenyl)-1,2-diphenylethane**

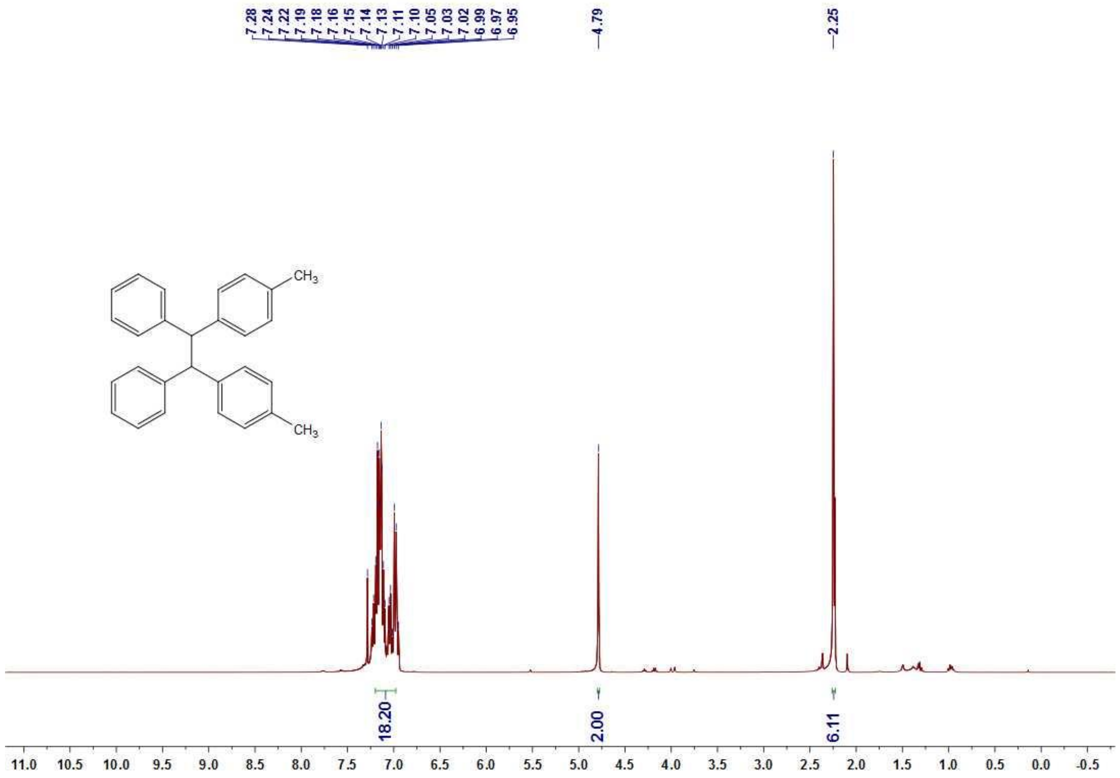

Supplementary Figure 68 ^{1}H NMR of **1,2-Diphenyl-1,2-di-p-tolylethane**

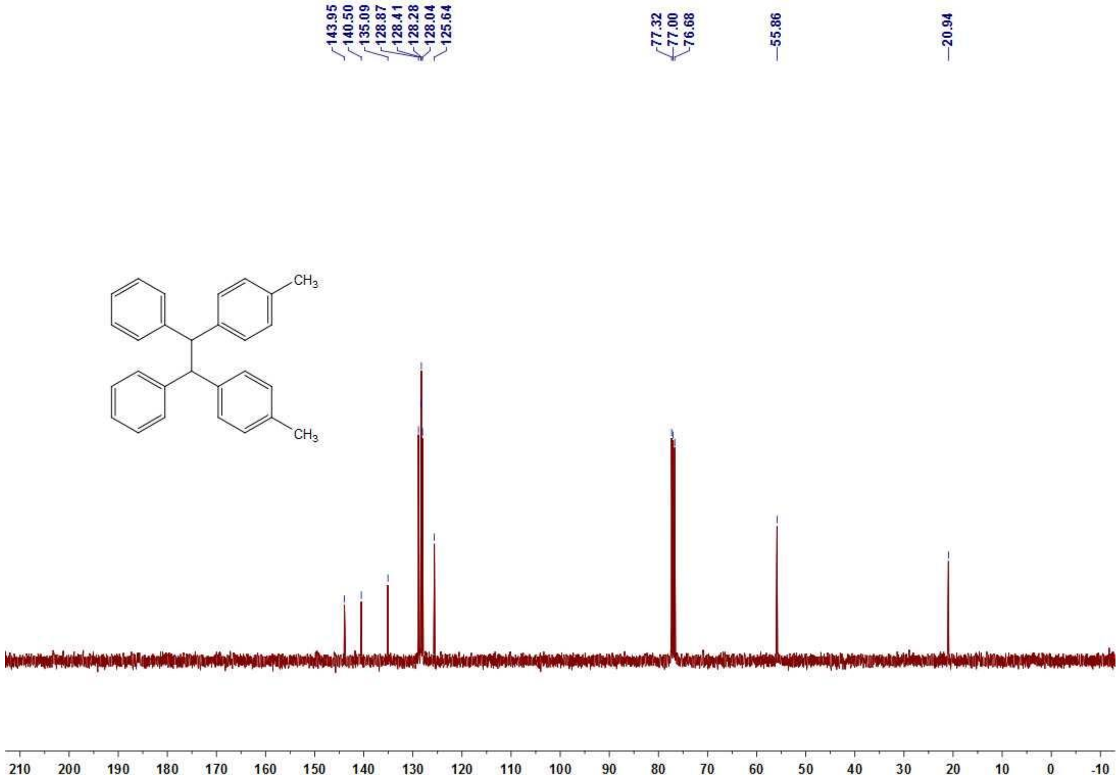

Supplementary Figure 69 ^{13}C NMR of **1,2-Diphenyl-1,2-di-p-tolylethane**

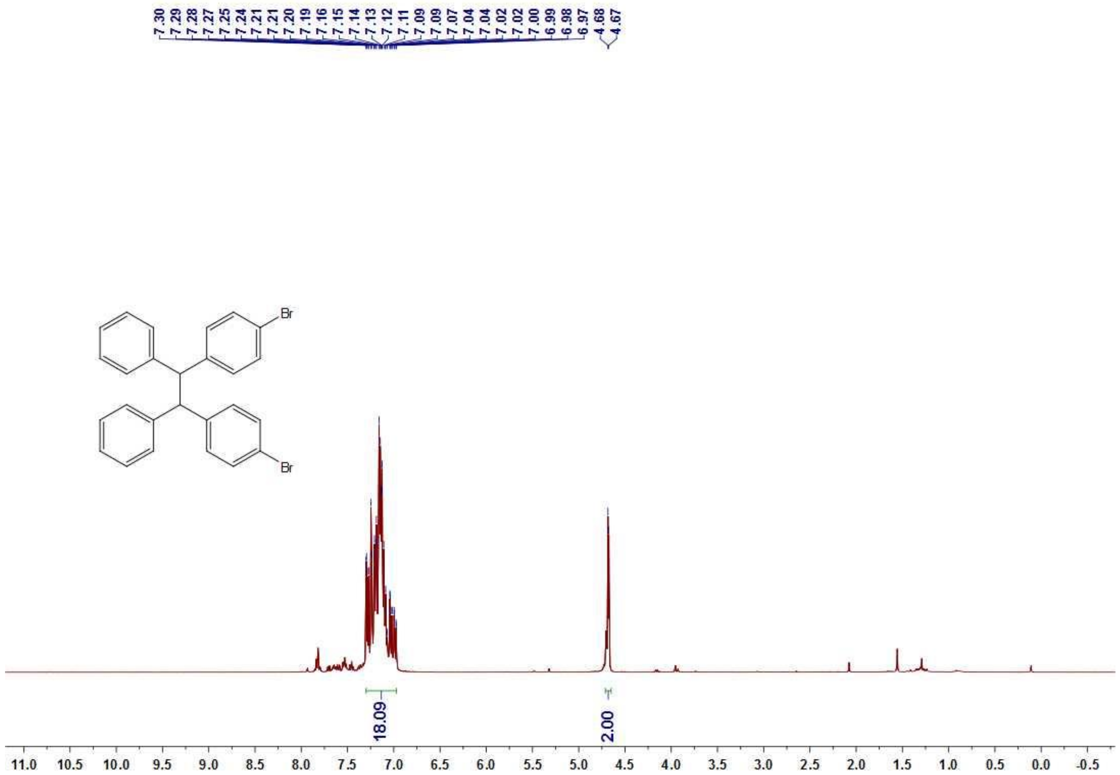

Supplementary Figure 70 ^{1}H NMR of **1,2-Bis(4-bromophenyl)-1,2-diphenylethane**

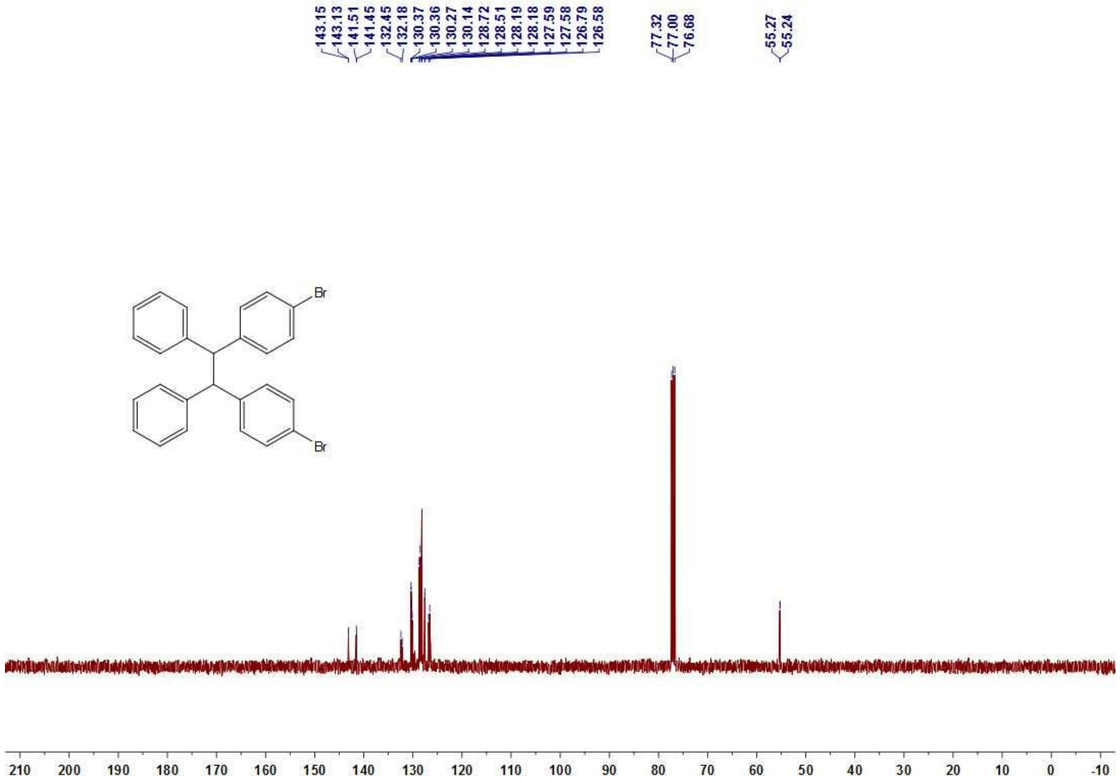

Supplementary Figure 71 ^{13}C NMR of **1,2-Bis(4-bromophenyl)-1,2-diphenylethane**

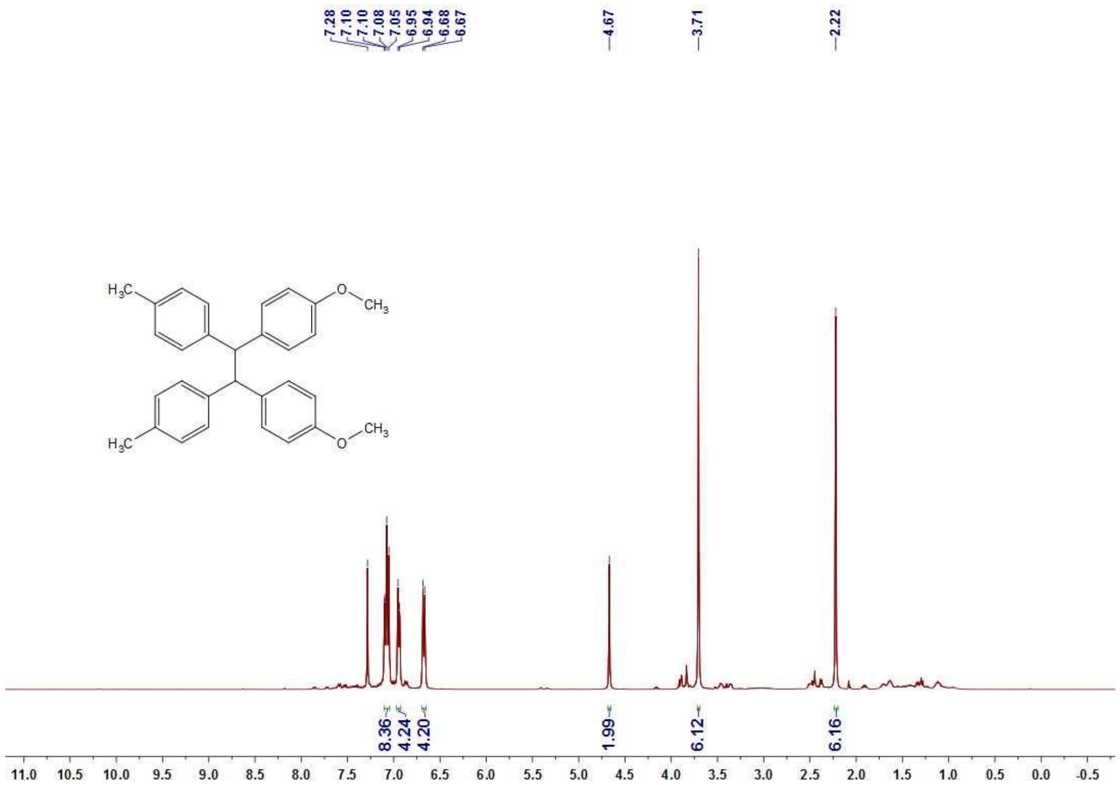

Supplementary Figure 72 ^{1}H NMR of **1,2-Bis(4-methoxyphenyl)-1,2-di-p-tolylethane**

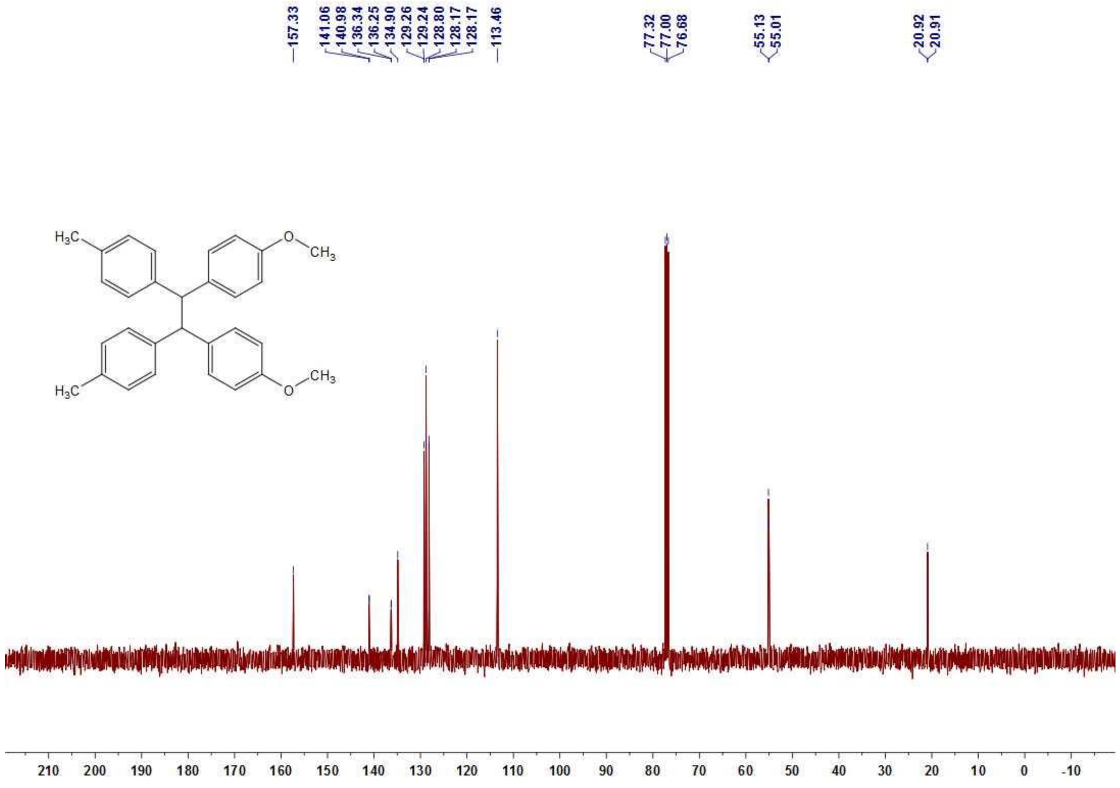

Supplementary Figure 73 ^{13}C NMR of **1,2-Bis(4-methoxyphenyl)-1,2-di-p-tolylethane**

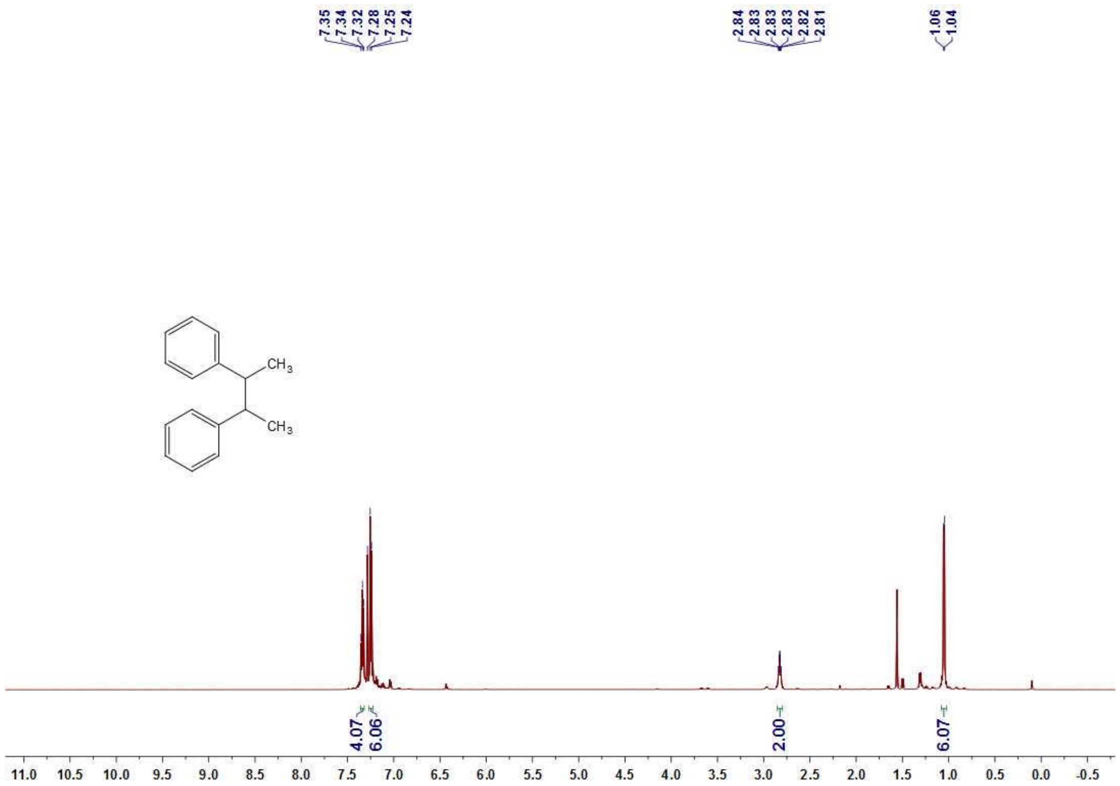

Supplementary Figure 74 ^{1}H NMR of **2,3-Diphenylbutane**

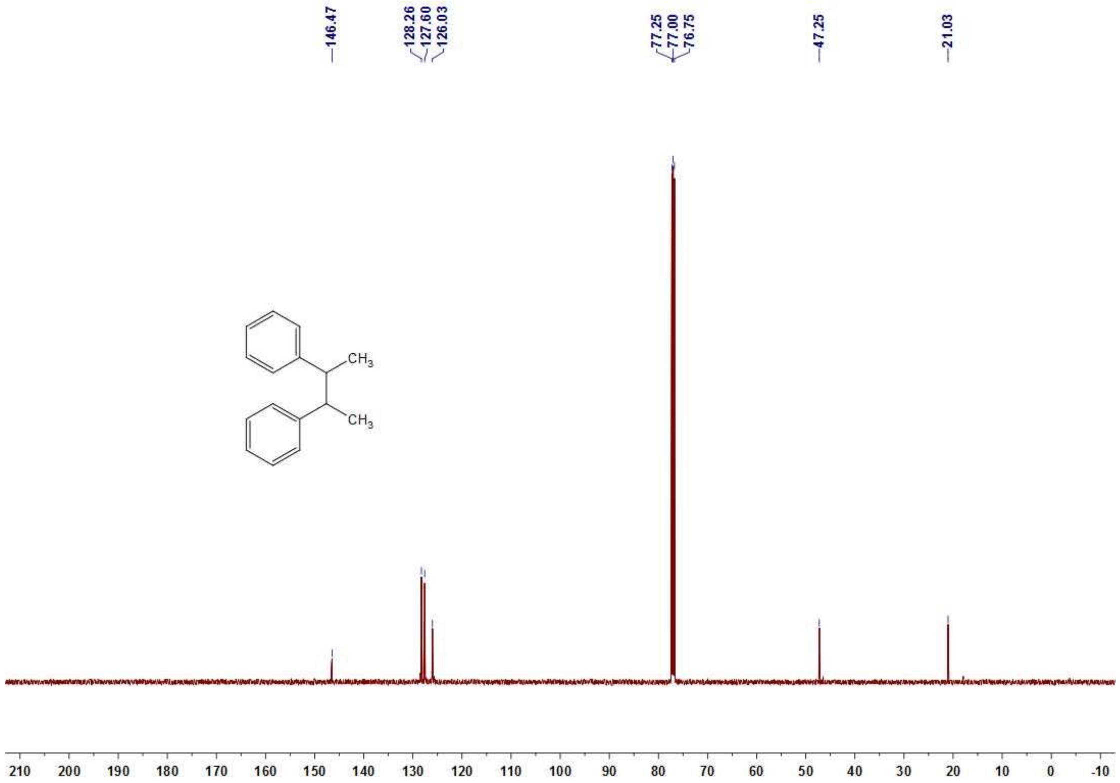

Supplementary Figure 75 ^{13}C NMR of **2,3-Diphenylbutane**

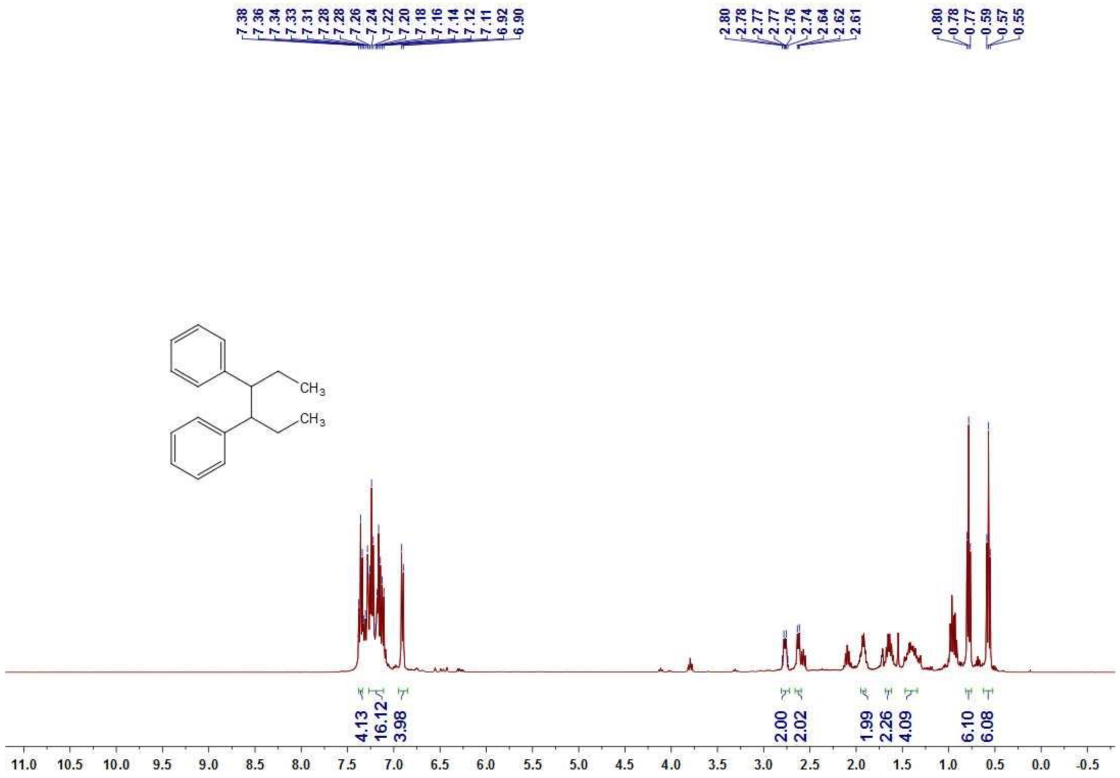

Supplementary Figure 76 ^{1}H NMR of **3,4-Diphenylhexane**

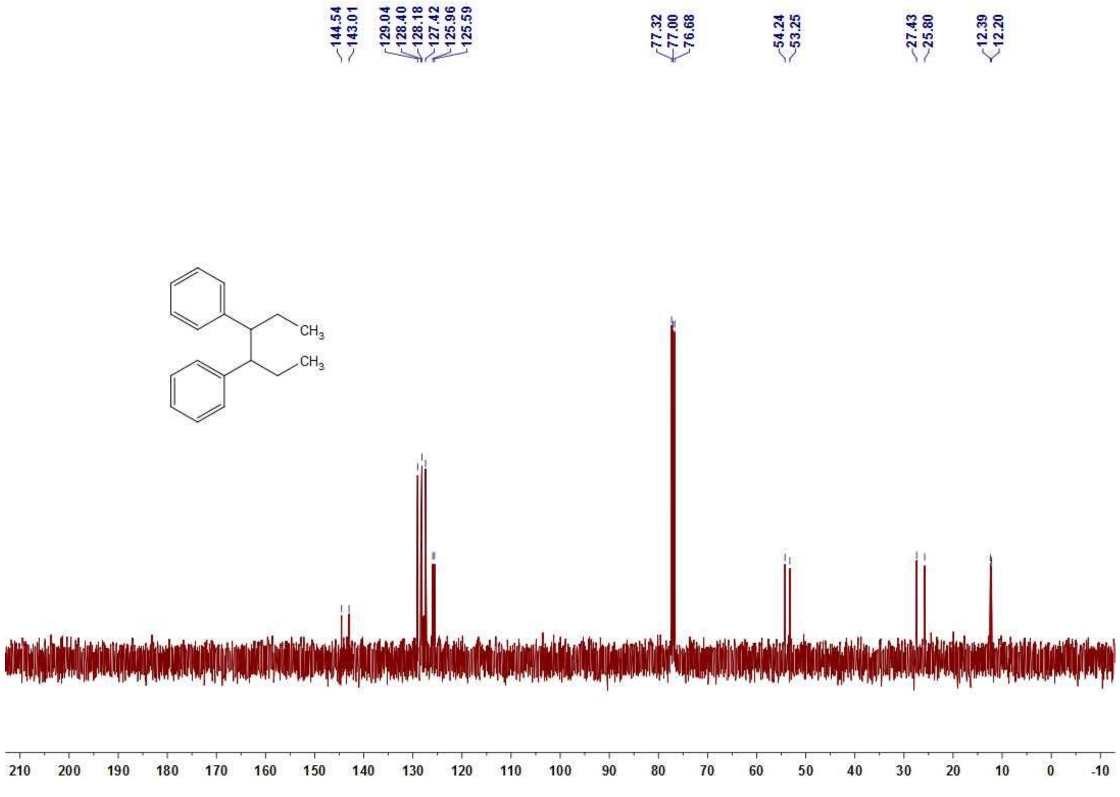

Supplementary Figure 77 ^{13}C NMR of **3,4-Diphenylhexane**

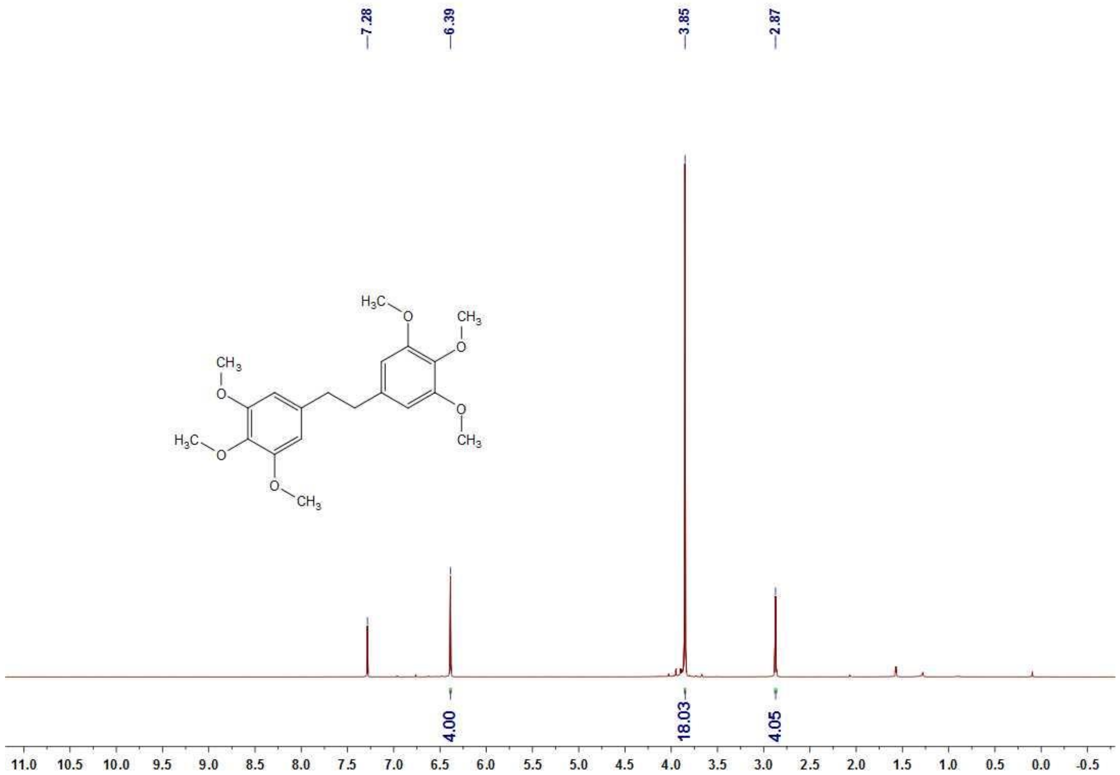

Supplementary Figure 78 ^{1}H NMR of **1,2-Bis(3,4,5-trimethoxyphenyl)ethane**

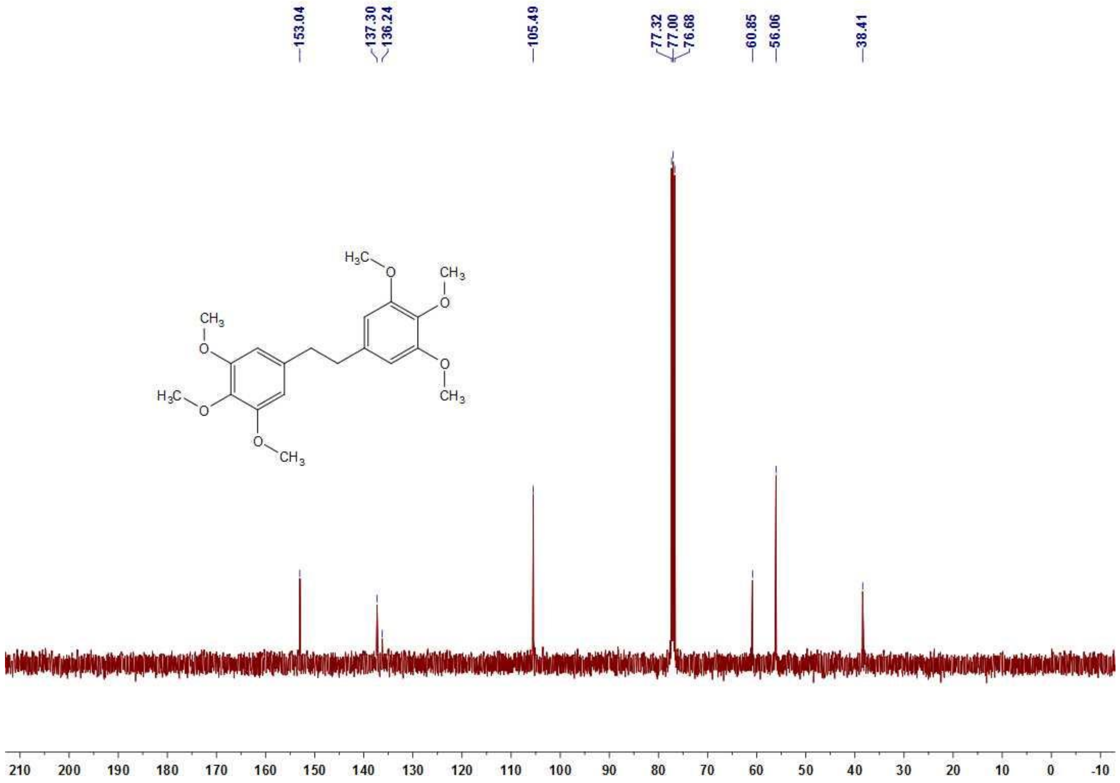

Supplementary Figure 79 ^{13}C NMR of **1,2-Bis(3,4,5-trimethoxyphenyl)ethane**

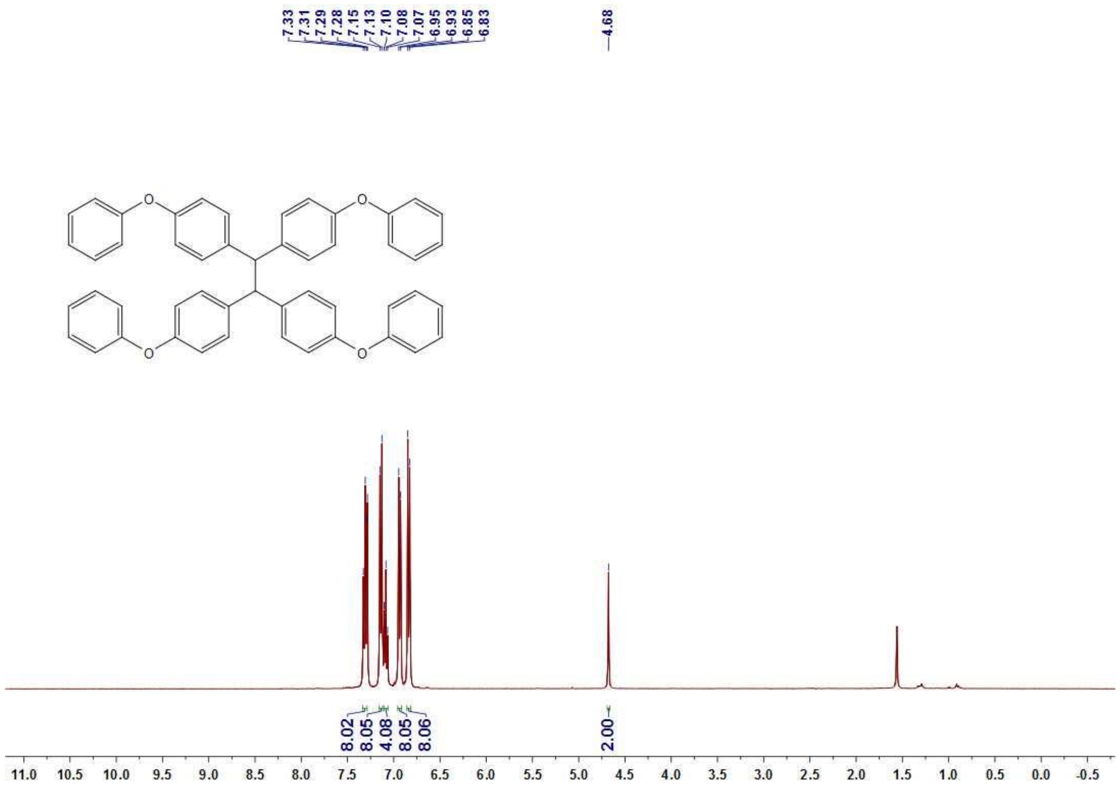

Supplementary Figure 80 ^{1}H NMR of **1,1,2,2-Tetrakis(4-phenoxyphenyl)ethane**

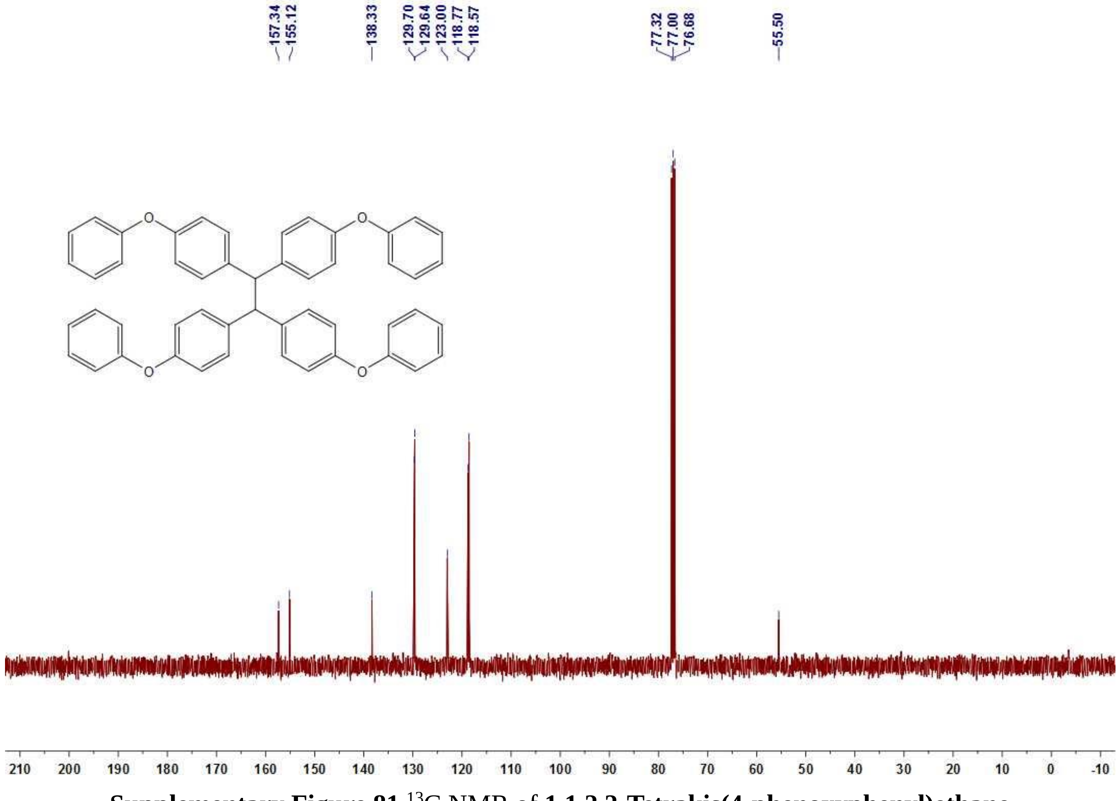

Supplementary Figure 81 ^{13}C NMR of **1,1,2,2-Tetrakis(4-phenoxyphenyl)ethane**

Ⅴ. Supplementary References

1. Nwachukwu, C. I.; McFadden, T. P.; Roberts, A. G., Ni-Catalyzed Iterative Alkyl Transfer from Nitrogen Enabled by the In Situ Methylation of Tertiary Amines. *The Journal of Organic Chemistry* **2020,** *85* (15), 9979-9992.
2. Bates, R. B.; Ogle, C. A., Arenes disubstituted with primary alkyl groups from xylylene dianions. *The Journal of Organic Chemistry* **1982,** *47* (20), 3949-3952.
3. Ueng, S.-H.; Makhlouf Brahmi, M.; Derat, É.; Fensterbank, L.; Lacôte, E.; Malacria, M.; Curran, D. P., Complexes of Borane and N-Heterocyclic Carbenes: A New Class of Radical Hydrogen Atom Donor. *Journal of the American Chemical Society* **2008,** *130* (31), 10082-10083.
4. Shakya, A.; Al-Hiari, Y.; Sweileh, B.; Abu Sheikha, G.; Almuhtaseb, S., Synthesis of 1-benzyl-1,2,3,4-tetrahydroisoquinoline, Part I: Grignard synthesis of 1-(substitutedbenzyl)-1,2,3,4-tetrahydroisoquinoline models with potential antibacterial activity. *Jordan Journal of Pharmaceutical Sciences* **2009,** *2*, 1-21.
5. Park, G.; Yi, S. Y.; Jung, J.; Cho, E. J.; You, Y., Mechanism and Applications of the Photoredox Catalytic Coupling of Benzyl Bromides. *Chemistry – A European Journal* **2016,** *22* (49), 17790-17799.
6. Gassman, P. G.; Guggenheim, T. L., The 3-fluoromethylphenyl group. a useful moiety for the study of solvolysis reactions with large negative rho-values. *The Journal of Organic Chemistry* **1982,** *47* (20), 4002-4004.
7. Liu, Y.; Zhang, D.; Xiao, S.; Qi, Y.; Liu, S., Copper-Catalyzed Homocoupling of Alkyl Halides in the Presence of Samarium. *Asian Journal of Organic Chemistry* **2019,** *8* (6), 858-862.
8. Chen, T.; Yang, L.; Li, L.; Huang, K.-W., Homocoupling of benzyl halides catalyzed by POCOP–nickel pincer complexes. *Tetrahedron* **2012,** *68* (31), 6152-6157.
9. Carter, T. S.; Guiet, L.; Frank, D. J.; West, J.; Thomas, S. P., Iron-Catalysed Reduction of Olefins using a Borohydride Reagent. *Advanced Synthesis & Catalysis* **2013,** *355* (5), 880-884.
10. Fallon, B. J.; Corcé, V.; Amatore, M.; Aubert, C.; Chemla, F.; Ferreira, F.; Perez-Luna, A.; Petit, M., A well-defined low-valent cobalt catalyst Co(PMe3)4 with dimethylzinc: a simple catalytic approach for the reductive dimerization of benzyl halides. *New Journal of Chemistry* **2016,** *40* (12), 9912-9916.
11. Maiuri, M.; Garavelli, M.; Cerullo, G., Ultrafast Spectroscopy: State of the Art and Open Challenges. *Journal of the American Chemical Society* **2020,** *142* (1), 3-15.
12. Yang, W.; Tat To, C.; Chan, K. S., Iodine-catalysed transfer hydrogenation of a carbon–carbon σ-bond with water. Organic & Biomolecular Chemistry **2019**,*17*(28), 6757-6761.
13. Yu, D.; To, W.-P.; Tong, G. S. M.; Wu, L.-L.; Chan, K.-T.; Du, L.; Phillips, D. L.; Liu, Y.; Che, C.-M., Luminescent tungsten(vi) complexes as photocatalysts for light-driven C–C and C–B bond formation reactions. *Chemical Science* **2020,** *11* (25), 6370-6382.
14. Mboyi, C. D.; Gaillard, S.; Mabaye, M. D.; Pannetier, N.; Renaud, J.-L., Straightforward synthesis of substituted dibenzyl derivatives. *Tetrahedron* **2013,** *69* (24), 4875-4882.
15. Fristad, W. E.; Klang, J. A., Silver(I)/persulfate oxidative decarboxylation of carboxylic acids. Arylacetic acid dimerization. *Tetrahedron Letters* **1983,** *24* (22), 2219-2222.
16. Alder, R. W.; Hyland, N. P.; Jeffery, J. C.; Riis-Johannessen, T.; Riley, D. J., Poly(1,1-bis(dialkylamino)propan-1,3-diyl)s; conformationally-controlled oligomers bearing electroactive groups. *Organic & Biomolecular Chemistry* **2009,** *7* (13), 2704-2715.
17. Manley, D. W.; Walton, J. C., A Clean and Selective Radical Homocoupling Employing Carboxylic Acids with Titania Photoredox Catalysis. *Organic Letters* **2014,** *16* (20), 5394-5397.

18. Gan, Y.; Hu, H.; Liu, Y., Nickel-Catalyzed Homo- and Cross-Coupling of Allyl Alcohols via Allyl Boronates. *Organic Letters* **2020,** *22* (11), 4418-4423.
19. Li, Y.; Kijima, T.; Izumi, T., Ruthenium-catalyzed carbon–carbon formation to synthesize tetraarylethanes and tetraarylxylylene through dechlorinative dimeric reaction. *Journal of Organometallic Chemistry* **2003,** *687* (1), 12-15.
20. Okajima, M.; Soga, K.; Nokami, T.; Suga, S.; Yoshida, J.-i., Oxidative Generation of Diarylcarbenium Ion Pools. *Organic Letters* **2006,** *8* (22), 5005-5007.
21. Greene, M. A.; Yonova, I. M.; Williams, F. J.; Jarvo, E. R., Traceless Directing Group for Stereospecific Nickel-Catalyzed Alkyl−Alkyl Cross-Coupling Reactions. *Organic Letters* **2012,** *14* (16), 4293-4296.
22.Navale, T. S.; Thakur, K.; Rathore, R., Sequential Oxidative Transformation of Tetraarylethylenes to 9,10-Diarylphenanthrenes and Dibenzo[g,p]chrysenes using DDQ as an Oxidant. *Organic Letters* **2011,** *13* (7), 1634-1637.
23. Ross, H., Front Cover: Chemistry—A European Journal Turns 25 (Chem. Eur. J. 1/2020). *Chemistry – A European Journal* **2020,** *26* (1), 1-1.
24. Lv, L. Y.; Zhu, D. H.; Qiu, Z. H.; Li, J. B.; Li, C.-J., Nickel-Catalyzed Regioselective Hydrobenzylation of 1,3-Dienes with Hydrazones. *ACS Catalysis* 2019, *9*(10), 9199–9205.
